# Flankophile: a bioinformatic pipeline for prokaryotic genomic synteny analysis

**DOI:** 10.1128/spectrum.02413-23

**Published:** 2023-12-12

**Authors:** Alix Vincent Thorn, Frank M. Aarestrup, Patrick Munk

**Affiliations:** 1 Research Group for Genomic Epidemiology, National Food Institute, Technical University of Denmark, Kgs. Lyngby, Denmark; University at Albany, Albany, New York, USA

**Keywords:** bioinformatics, antibiotics resistance, veterinary microbiology, software, phylogenetic analysis, epidemiology, sequence analysis, genomics, metagenomics

## Abstract

Analysis of flanking region sequences can be useful when comparing mobile prokaryotic sequences from different bacterial isolates or metagenomes. Here, we report a new bioinformatics tool, a pipeline called Flankophile, which can analyze flanking regions and sequence variants. The main feature of Flankophile is that it visualizes flanking region synteny and sequence variants in publication-ready plots with distance trees, gene annotations, and metadata. Antimicrobial resistance is a significant threat to human health, and surveillance of the spread of antimicrobial resistance is essential. The study of acquired antimicrobial resistance genes (ARGs) is an obvious use case for Flankophile due to the typically diverse genetic context of the genes. Flanking sequences offer a stronger epidemiological signal than the gene sequence alone. To demonstrate Flankophile, we applied it to 2,057 sequenced bacterial isolates and 273 metagenomes from humans and pigs in Denmark and compared the ARGs found in each host reservoir. Gene variant results showed that just ca. 4% of all unique ARG variants found (*N* = 1,052) were detected in samples from both humans and pigs. Flanking region analysis of ARG variants frequently detected in this study showed multiple examples of genes where the entire 3,000-base pair flanking region was identical in samples from both hosts but also cases where no overlap was observed, and the clustering showed a complete separation of the samples from pigs and the samples from humans.

**IMPORTANCE:**

The Flankophile pipeline enables the analysis and visualization of flanking regions of prokaryotic sequences of interest on large data sets in one step and in a consistent manner. A specific tool for flanking region analysis with automated visualization has not been developed before, and Flankophile will make flanking region analysis easier and accessible to more people. Flankophile will be especially useful in the field of genomic epidemiology of acquired antimicrobial resistance genes. Here, information from flanking region sequences can be instrumental in rejecting or supporting the possibility of a recent common source of the same resistance gene found in different samples.

## INTRODUCTION

Antimicrobial resistance (AMR) is a serious and increasing threat to human health globally (1). While it is generally accepted that antimicrobial use will select for AMR, the transmission of AMR bacteria and antimicrobial resistance genes (ARGs) is also important for the occurrence and abundance of AMR (2, 3). Thus, surveying the spread of ARGs across geographical borders, as well as different animal hosts and the environment, is important for designing interventions against the spread of AMR (4). However, while several studies have documented the transmission of specific AMR clones globally and between hosts (5), it has been more difficult to determine transmission of specific ARGs (4, 6, 7). The epidemiological importance of ARGs found in non-pathogenic bacterial species from environmental samples and livestock for human health has been an area of debate (4, 8).

The ResFinder Database (9, 10) contains more than 3,000 acquired ARGs that confer resistance to various classes of antimicrobials. Some of the ARGs are alleles of the same gene and vary only by one single-nucleotide polymorphism. Regardless of the database used, there may be sequence variants that are common in nature but not in the database. When performing analysis on bacterial genomes or metagenomes, it is therefore useful to include not only the closest match in the database but also the identified unique variant sequences to maximize the phylogenetic signal and resolution.

When analyzing the transmission of ARGs, the starting point is often to establish how variants of ARGs are dispersed geographically and in which bacterial species. Since many ARGs have been mobilized multiple times and are widespread, the mere presence of the same ARG in different reservoirs is not sufficient to prove any recent common transmission or shared source. Since the sequences of ARGs are evolutionary conserved, there is often too little phylogenetic signal for epidemiology on short time scales in the gene sequence alone. Combining analyses of ARG variants with analysis of flanking regions can provide important additional information when analyzing acquired ARGs (3, 11). The flanking regions of the same ARG can be very diverse since they often become captured by mobile genetic elements like conjugative transposons (12). Even if the genetic environment is homologous, differences in the flanking region sequences arising from mutations increase the epidemiological signal, thus increasing our likelihood to identify the transfer of ARGs between different reservoirs. Flanking region analysis also provides new information on risks of co-selection through genetically linked ARGs and the hosting organism.

A bioinformatics tool Flanker (11) has previously been published and can perform some of the steps needed for flank analysis. Flanker searches the input sequences for reference genes of interest selected from one of the built-in reference databases. Then, it extracts the flanking region sequences around an annotated gene of interest, and it can also cluster the flanking regions (11). To meet the need for automated analysis and visualization of flanking regions and variants of sequences such as ARGs, we here present a new tool called Flankophile that has several advantages over Flanker. The Snakemake implementation allows for easier distributed searching over many computers, and we allow the user to choose among several distance algorithms. Importantly, it also automates the entire process, including annotation of flanks and production of high-quality figures instead of relying on the user to carry out and integrate different analysis results. Flankophile automatically creates publication-ready gene-annotated distance tree plots with gene variants and outputs tabulated results for the user. Flankophile is flexible when it comes to the choice of reference database since you can use any FASTA-formatted file with DNA sequences as your own personal reference database.

To demonstrate the general features of Flankophile and its usefulness for studies in, e.g., ARG epidemiology, we applied the tool to a wide collection of 2,006 clinical isolates collected from hospitals in a single day in Denmark. Just five species together made up 66% of the data: *E. coli*, 707; *S. aureus*, 344; *K. pneumoniae*, 94; *E. faecalis*, 92; *S. agalactiae*, 86). From Danish, healthy pigs, we also included 51 *E. coli* (the most common clinical species) genomes and 273 metagenomes. While we here apply Flankophile to ARGs, the tool can be used to study the flanking regions of any sequence of interest.

## RESULTS

**Fig 1:**
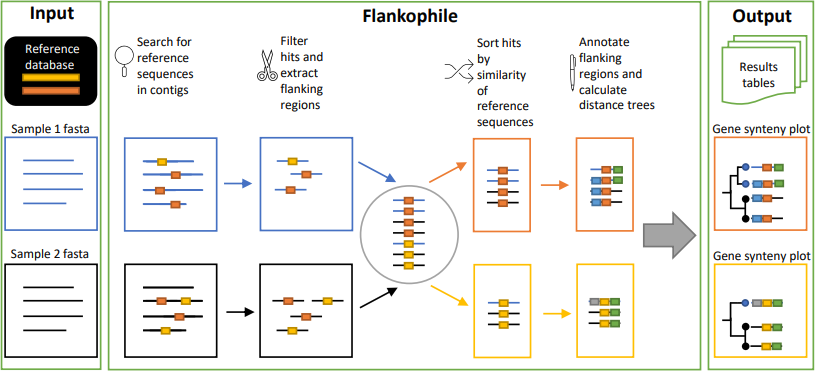
Diagram overview of the Flankophile pipeline. As input data, the pipeline expects DNA contigs, e.g., from assembled genomes or metagenomes. Any collection of user-supplied sequences can be used as a reference database. Both the input data and reference database should be (multi-)FASTA files. Flankophile searches the input sequences for matches to the reference database. Hits with flanking regions of the required length for flank analysis are selected, and their flanking region sequences are extracted. Hits that matched to similar reference sequences are clustered into groups. Genetic features in the flanking regions are annotated, and three distance matrices are calculated on the sequences in each group—one based on the flanking region, one based on the target region, and one based on a combination. Distance trees are made from the distance matrices using hierarchical clustering and plotted along with annotation arrows, gene variant information, and metadata. Output includes plots in PDF format and results tables.

The Flankophile pipeline can be downloaded from Bitbucket (https://bitbucket.org/genomicepidemiology/flankophile), and its functionality is highlighted in Fig. 1.

### Antimicrobial resistance genes detected by Flankophile

**Fig 2:**
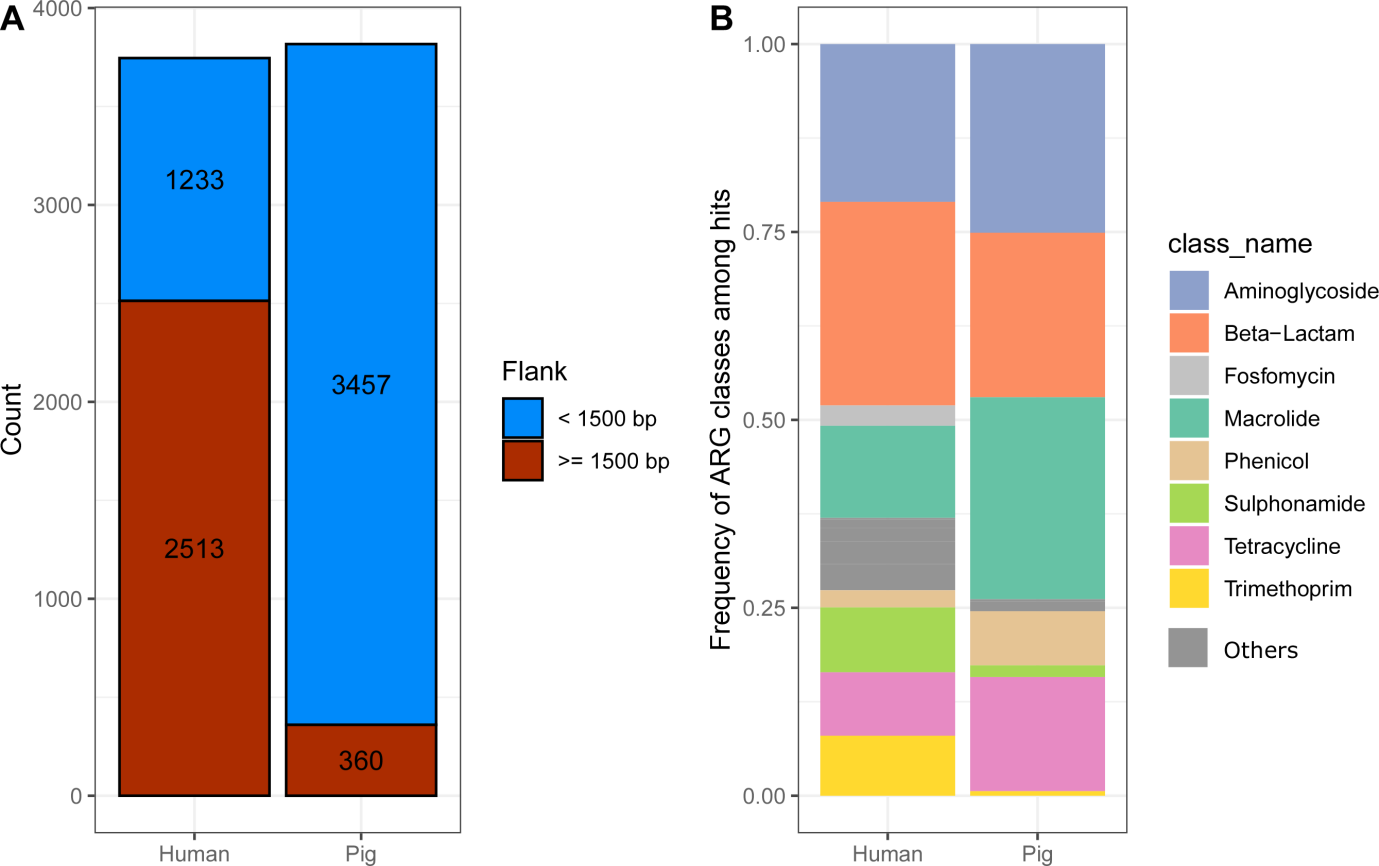
ARGs detected in samples from Danish humans and pigs. (**A**) The distribution of ARGs detected in bacteria of human or porcine origin included in synteny visualizations; those with at least 1,500 bp up- and downstream of target genes. (**B**) Frequency of AMR classes related to hits found in humans and pigs, respectively. The most frequent AMR classes are shown, while the remaining classes are combined in the dark gray.

Using the ResFinder database (10) as reference database, we applied Flankophile to a collection of Illumina shotgun-sequenced data sets from 2,006 Danish human clinical isolates as well as from *E. coli* isolates and 273 metagenomes from Danish pigs (Table 1). Flankophile identified a total of 7,563 occurrences of ARGs; 3,817 in the pig metagenomic data sets and 3,746 in the human data sets (Fig. 2A). Two-thirds of the hits from the human data set had the required upstream and downstream 1,500 base pairs in their flanking region sequences for the subsequent flank visualization. That was only the case for around 1/10 of pig hits (Fig. 2B). This is likely because the majority of the pig samples and ARG hits came from metagenome data sets, while all the human data sets were from clinical isolates. Assemblies of metagenomes typically result in shorter contigs due to shared sequences among strains in the microbiome.

**TABLE 1:** Evaluation data set^*a*^

	Data set name	European Nucleotide Archive project number	Number of assembled data sets	Sample type	Host	Collection years
1	One Day in Denmark	PRJEB37711	2,006	Human clinical isolate genomes of many different species	Human	2018
2	EFFORT Isolates	PRJEB41365	51	*E. coli* Isolates—pig feces	Pig	2015
3	EFFORT Metagenomes	PRJEB22062	21	Metagenome—pig feces	Pig	2015
4	Danish VETII project	PRJEB26961	210	Metagenome—pig feces	Pig	2014–2016
5	Danmap 2	PRJEB50613	42	Metagenome—pig feces	Pig	2015–2018

^*a*^ Overview of the data used as input for Flankophile. Detailed input list of data set with public accession codes can be found in Table S1. The first column contains the data set prefix in parentheses that is used for each observation in the Flankophile figures.

The identified ARGs most frequently encoded resistance to sulfonamides, aminoglycosides, beta-lactams, macrolides, and tetracycline. Both beta-lactam and aminoglycoside ARGs were among the three most frequent AMR classes in both hosts. Sulfonamide ARGs were around five times as frequent in humans as in pigs. Trimethoprim resistance had a much higher prevalence in human feces, whereas tetracycline and macrolide were more dominant phenotypes in the pig resistomes (Fig. 2B).

### Variant analysis shows that few unique ARG variants are shared in the human and porcine resistome

**Fig 3:**
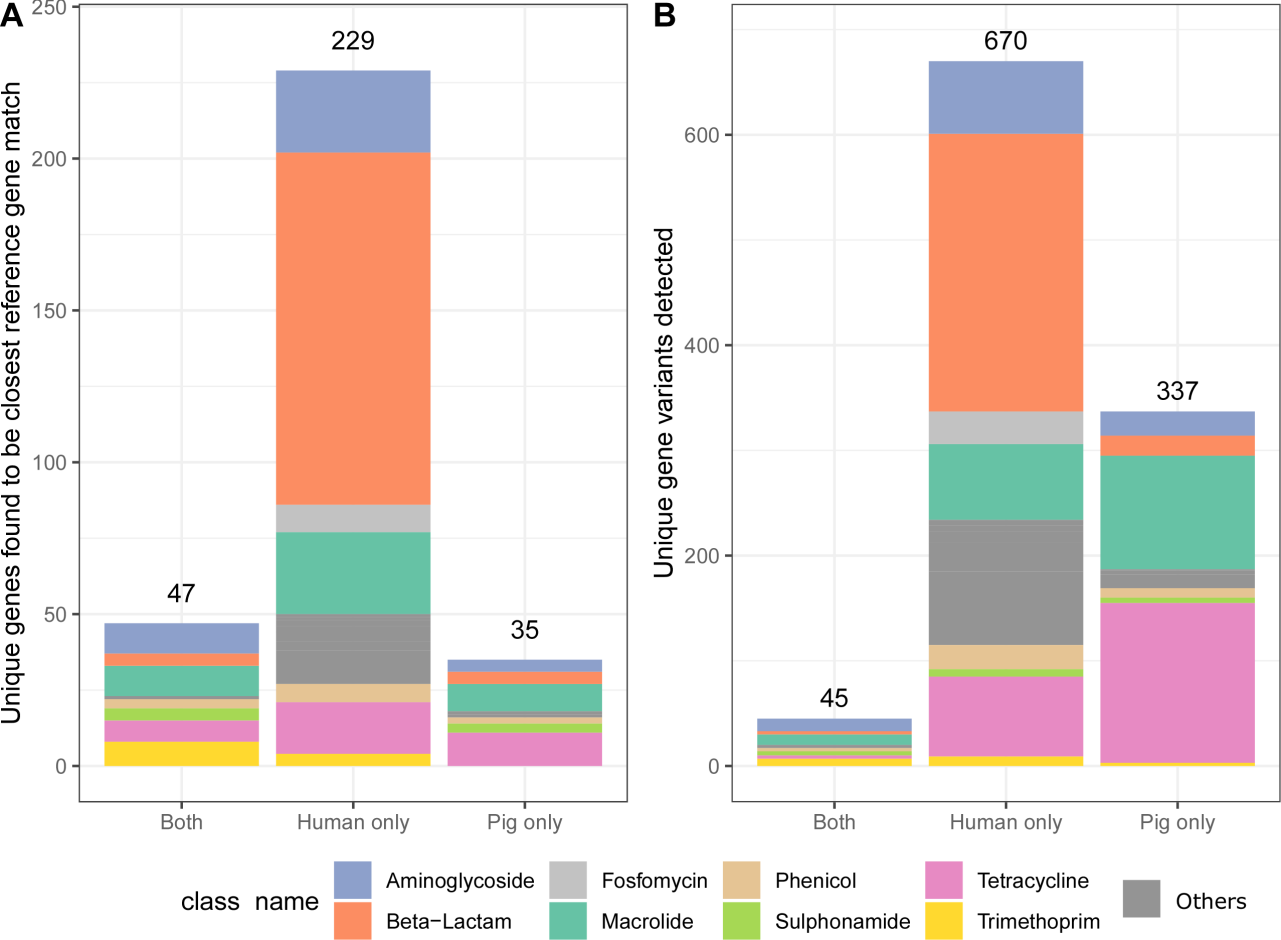
The host spectrum of ARGs identified across the genomic assemblies by Flankophile. (**A**) Number of unique ARG reference sequences detected as the closest match to one or more ARG hits from the study. (**B**) Number of unique ARG variants detected in one or more hits from the study.

**Fig 4:**
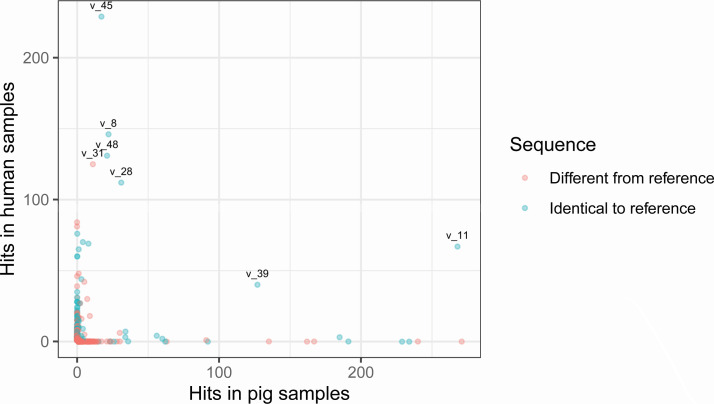
Scatterplot of the number of observations per host for 1,052 unique ARG sequences found in the study. The 7 ARG sequences that were observed at least 10 times in samples of human and of pig origin are marked with the name given to their unique sequence in the study. The dots are transparent, and darker dots represent multiple dots that overlay.

Of the 311 distinct ARG reference sequences that were found to be the best match to one or more ARG hits in the study, 11% were found only in the pig data sets, 74% found only in the human data sets, and 15% were found in both human and pig data sets (Fig. 3A). When considering the unique gene variants and not just the closest match in the reference database, Flankophile identified a total of 1,052 unique ARG sequence variants in input data. Just 4% of the unique ARG variants were seen at least once in both pigs and humans (Fig. 3B), and most ARGs were found exclusively or primarily in one host (Fig. 4). Also, 10% of ARG variants were detected at least 10 times in pig or in humans, but just 0.66% (7 ARG variants) were detected at least 10 times in both (Fig. 4). When studying the subset of reference genes that were found to be the closest match to any hits in the database, it looks like there were not any sulfonamide ARGs that were found only in samples from humans (Fig. 3A). In contrast, the variant results show that there are actually seven unique sulfonamide resistance-encoding gene variants that are uniquely found in the human data set (Fig. 3B). Also, 45% of unique ARG variants found only in pigs encode resistance toward tetracycline, and 39% of unique ARG variants found only in humans encode resistance toward beta-lactams (Fig. 3B).

### Most unique ARG gene variants detected differed from the reference sequences

Of the 1,052 unique ARG variants found in the study, 17% were 100% identical to reference sequences in the ResFinder database (9), while 83% had minor differences in the sequence. Notably, there are many examples of ARG variants different from the reference sequence that have a high number of observations (Fig. 4).

### Flanking region analysis

**Fig 5:**
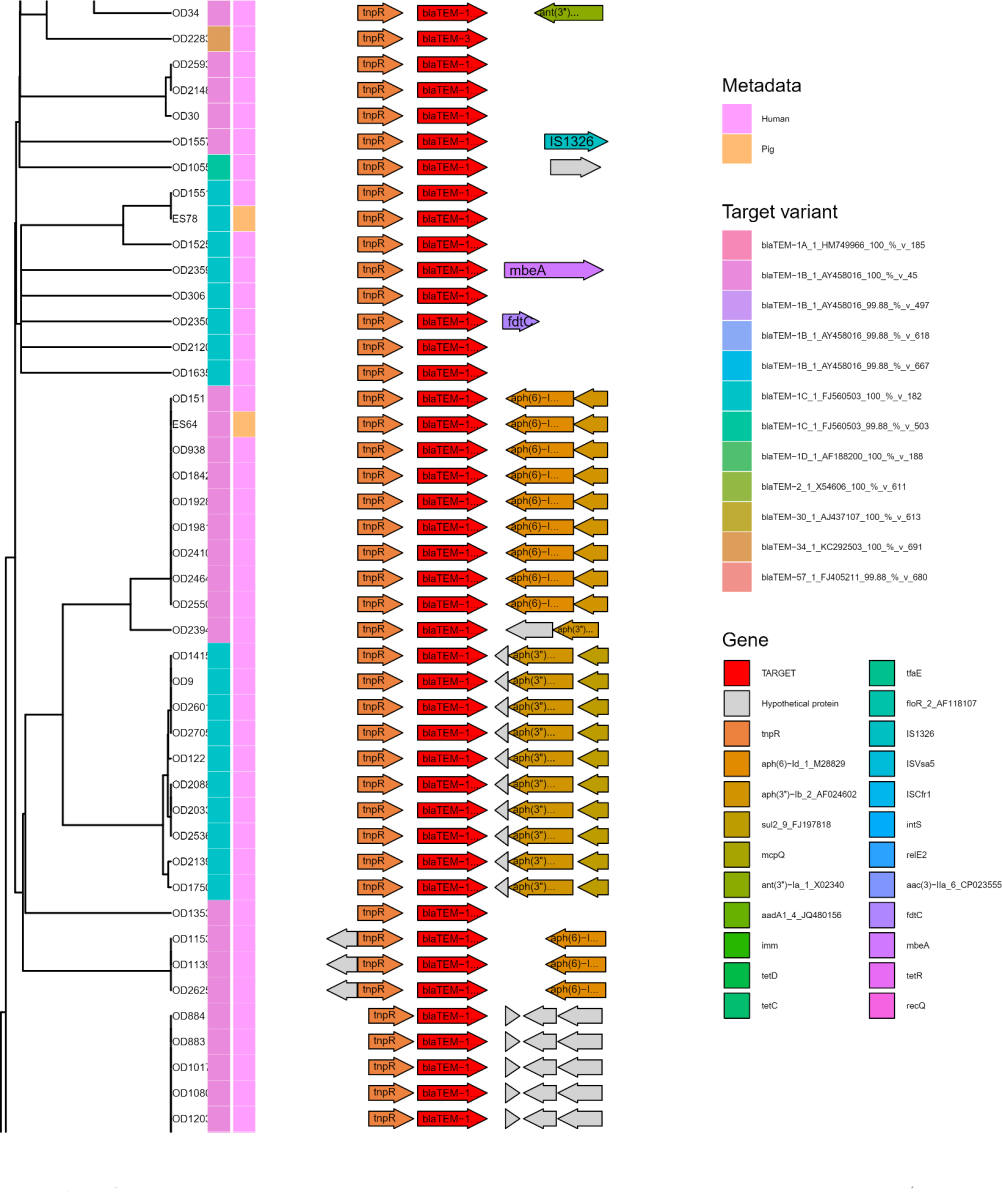
Subset of Flankophile plot for cluster 1—blaTEM. Gene synteny plot of all blaTEM hits from the study that had an upstream and downstream flanking region length of at least 1,500 bp. From left to right: Distance tree made with hierarchical UPGMA (Unweighted Pair Group Method with Arithmetic Mean) cluster from the Jaccard distance of the blaTEM flanking regions. Color annotation columns represent the target variant (left) and the host species (right). The right panel with arrows depicts the gene synteny, with the target sequence, centered on the Flankophile target sequence in the middle (red). See Table S1 for data set abbreviations (“OD” and “ES” sequences are from isolated, bacteria assemblies). See full plot in Fig. S1 in plot named “Cluster 1_blaTEM_57_1_FJ405211.”

**Fig 6:**
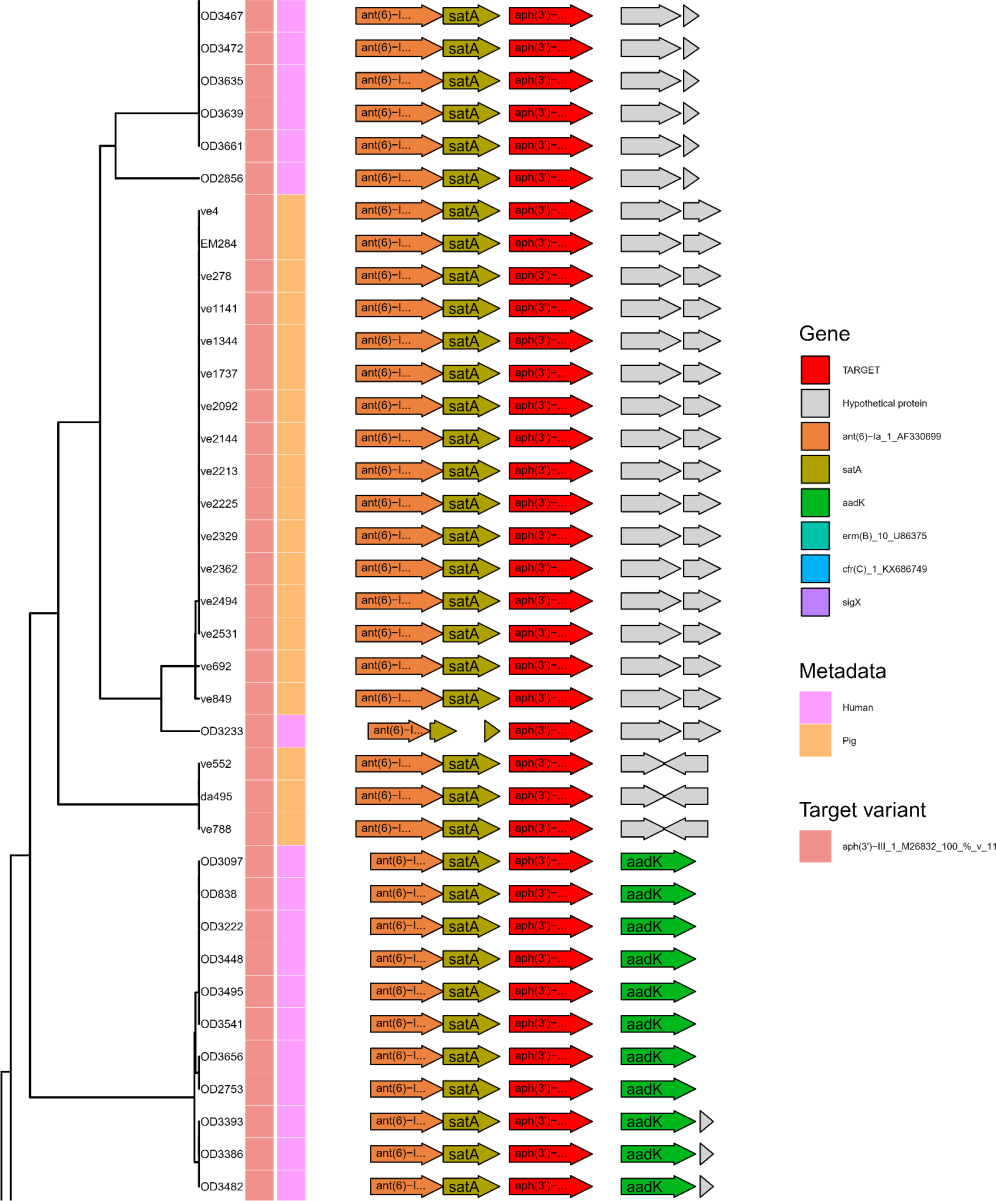
Subset of Flankophile plot for cluster 58—aph(3*′*)-III. The figure depicts a gene synteny plot of all the aph(3*′*)-III hits from the study that had an upstream and downstream flanking region length of at least 1,500 bp. See Fig. 5 for details on annotation. See Table S1 for data set abbreviations (“ve,” “EM,” and “da sequences are from metagenomic assemblies). See full plot in Fig. S1 in plot named “Cluster 58_aph_3__III_1_M26832.”

The ARG hits that had at least 1,500 base pairs both upstream and downstream (Fig. 2B) were included by Flankophile in the flanking region analysis. Based on the reference sequences, the hits were clustered into 165 ARG clusters. Seven clusters contained the ARG variants that were observed at least 10 times in samples of human and of pig origin (Table 2). Flankophile’s gene synteny plots based on distance trees of the flanking region sequences of the seven clusters can be seen in Fig. S1. Two examples of these plots are provided in Fig. 5 and 6.

**TABLE 2:** Overview of exact ARG variants detected at least 10 times in both human-derived and pig-derived samples^*a*^

Cluster number	Gene name	Variant name	Number of hits—pig samples	Number of hits—human samples	% Identity compared to reference gene
1	blaTEM-1B_1_AY458016	v_45	17	229	100
27	aph(3*''*)-Ib_5_AF321551	v_8	22	146	100
28	sul2_2_AY034138	v_28	31	112	100
58	aph(3*'*)-III_1_M26832	v_11	268	67	100
132	sul1_5_EU780013	v_31	11	125	99.89
140	aph (6)-Id_1_M28829	v_48	21	131	100
162	ant (6)-Ia_1_AF330699	v_39	127	40	100

^*a*^ The first column refers to the first part of the plot name in Fig. S1. The number of hits found stated in the table may be larger than the number of times the variant was included in the gene synteny plots due to not all hits having the required flank lengths for flanking region analysis. All seven variants had 100% coverage compared to their references.

Figure 5 depicts a subset of the plot for cluster 1 (which contains *bla*TEM-1A, *bla*TEM-1B, *bla*TEM-1C, *bla*TEM-1D, *bla*TEM-2, *bla*TEM-30, *bla*TEM-34, and *bla*TEM-57). The flanking region analysis data contained numerous flanked ARGs from humans and just two derived from pigs. The two-pig ARG flanking regions cluster among the human ones, and thus, it cannot be excluded that transmission between the two reservoirs has occurred. See the full plot in Fig. S1.

As seen in Fig. S1, cluster 27 (*aph*(3*''*)−Ib, *aph*(3*''*)−Ib), cluster 140 (*aph* (6)−Id), cluster 132 (*sul*1_5), and cluster 28 (*sul*2_2 and *sul*2_3) all show multiple pig flanking regions that are identical to various human flanking regions. Cluster 162 (*ant* (6)−Ia) only contains one-pig AMR gene observation. It is not identical to any human observations.

Figure 6 shows a subset of the plot for cluster 58 where all the ARG hits were identical to the reference sequence of *aph*(3*'*)-III, see full plot in Fig. S1. In this cluster, there appears to be a separation by host in the clustering of the flanking regions, thus, suggesting that there is no or little transmission of this ARG between the two reservoirs.

## DISCUSSION

We developed and implemented Flankophile, a freely available bioinformatics tool for analyzing the immediate genomic contexts and variants of user-specified prokaryotic sequences of interest. Because the inputs are simply assemblies, it is feasible to integrate analysis of data sets from whole genome sequencing, metagenomics, and single-cell sequencing produced with various technologies. As an example, we here show its capacity to analyze the gene synteny around acquired ARGs, which are notoriously mobile. We applied Flankophile on a combination of microbial genomic and metagenomic data sets from Danish humans and pigs. This was done to demonstrate the tool capabilities and to investigate whether resolving gene variants and flanking regions would provide additional information of the possible transmission of ARGs between livestock and humans in Denmark. Our comparison was only done on a limited number of data sets, but when simply comparing overlap in ARGs, 15% of the genes were shared between the reservoirs. When further comparing at the ARG sequence variant level, this was reduced to 4%. Additionally, utilizing the flanking region on a subset of the gene clusters, we found examples of flanking regions from pig-derived sequences that were identical to those in human-derived sequences, suggesting potential sharing of genes between reservoirs. Synteny analysis results like those for *aph*(3*'*)-III show that Flankophile can help the user identify whether propagation of a gene happens within or between reservoirs, even when gene itself is identical.

Flankophile can help distinguish whether a widespread sequence is found in multiple data sets due to a recent common source or just because it is common. Such information may be very valuable when attempting, e.g., source attribution of ARGs in humans. Flankophile includes all hits in the overall results tables and in the variant file, but hits on contigs that cannot fulfill the required flank length are not included in the gene synteny plots. A current challenge is, therefore, that most sequencing is performed with short Illumina reads, often resulting in more fragmented assemblies. This is especially difficult for ARGs that are often flanked by repetitive sequences. This limitation is, however, likely to go away as longer and accurate reads and assemblies are becoming more common with technologies such as Oxford nanopore (13).

Even though our use case shows the potential value of including flanking region information in epidemiological analyses, any conclusions should still be drawn with care. We highlighted Flankophile on a limited number of sequenced genomes and metagenomes, and it was only possible to obtain sufficient flanking regions for 1/10 of ARGs from the pig metagenomes. One therefore needs to carefully consider how quantities derived from metagenomic assemblies are interpreted. With the data filtering happening in our pipeline, one should not count the absence of evidence as evidence of absence. Sequence data set complexity will determine assembly quality and the extent to which we can recover flanked genes and evidence of mobilization. Results are in that sense qualitative and have most value in cases where many flanks can be extracted and compared across groups of data sets. However, with further data and longer accurate sequencing reads, we do believe that such analyses have the potential to drastically improve our understanding of ARG evolution and transmission between reservoirs and bacterial species.

More than 80% of the unique ARG variants were not identical to reference sequences. Since some of these were observed many times, there seem to be more common gene variants out there than found in even an extensive reference database such as the ResFinder database. Flankophile is well suited to discover and report such new ARG variants. Importantly, Flankophile can also be used in other fields than the study of ARGs as any sequences can be used as reference database. Such other use cases could be transmission of virulence genes, the cargo genes of transposase genes, metabolic genes, etc.

## MATERIALS AND METHODS

The first subchapters here are describing how the Flankophile pipeline works in its different stages and has headers prefixed with “Flankophile.” Later subchapters relate to the specific use case that we highlight here (AMR in Danish pigs and people), and how we configured the pipeline and ran used the output. These chapters have headers prefixed with “Use case.”

### Flankophile: pipeline overview

Flankophile is a command-line bioinformatics pipeline written in the Python-based workflow manager Snakemake. Miniconda (tested with version 4.11.0) (14) and Snakemake (tested with version 6.9.1) (15) need to be installed to run Flankophile. The pipeline automatically sets up the conda environment needed for the analysis. Flankophile is freely available for download at https://bitbucket.org/genomicepidemiology/flankophile. It comes with a config file which enables the user to dictate certain settings and thresholds for tools used by the pipeline. The config file also contains the path for a user-defined reference database and an input list created by the user. The reference database can be any user-supplied multi-FASTA file containing DNA sequences of interest to search for in the input data. The input list is a tab-separated file containing the file paths to each input file as well as an optional metadata column, allowing the user to annotate groups of data sets in the output plots. Each input file is a multi-FASTA file which could be a combination of, e.g., bacterial draft genomes, metagenomic assemblies, and single-cell-assembled genomes.

### Flankophile: search and detection of user sequence variants

The first step of Flankophile is to index the reference database of user sequences and search for those in the input files using the tool ABRicate (16), which uses Blast (17) version 2.12.0 and any2fasta (18) version 0.4.2. The user controls the values of ABRicate filtering flags “—minid” (minimum DNA percent identity compared to closest reference sequence) and “—mincov” (minimum DNA percent coverage compared to closest reference sequence). Values from 90% to 100% are accepted, with a default of 98%. Simultaneously Seqkit (19) version 2.1.0 is used to find the length of each contig/sequence in the input files using the command “fx2tab.” Flankophile combines the ABRicate results from all input files into one file and expands the main results table also to include a unique identifier for each hit, the length of the contig/sequence where each hit was found, and metadata. BEDTools (20) version 2.30.0 is used to extract the hit sequences using the command “getfasta,” and the sequences are sorted with Seqkit (19) command “sort.”

DupRemover (21) version 1.0.3 is used to remove duplicates of sequences, and then each unique sequence variant is given a variant ID. The variant ID corresponding to each hit is added to the main results table.

### Flankophile: filtering on flanking region length and extraction of flanking sequences

The next step is the flanking region analysis. When target hits are found close to the start or end of the contig, there is less flanking region to analyze. Flankophile filters the hits according to a user-defined threshold for the minimum number of base pairs present upstream and downstream of the target. The rule is run for each individual seed so that one seed can easily count as the flank of another. Target hits that do not meet the threshold are excluded from the flanking region analysis. The main results table is filtered, and BEDTools (20) “getfasta” is used to extract target variants with and without their flanking regions. The BEDTools (20) command “maskfasta” is used to make a version of the flanking sequence where the user target sequence is masked.

### Flankophile: clustering of hits with similar reference sequence

All reference sequences that are found to be closest match to a hit used in the flanking region analysis are extracted with Seqkit (19) “grep.” These sequences are clustered according to percentage identity using CD-HIT-EST from the tool CD-HIT (22) version 4.8.1 using the more accurate but slow mode with unlimited memory usage. The CD-HIT-EST flag settings for sequence identity threshold value and for the length difference cutoff values control how similar sequences must be in order to be clustered together. These two flags are defined by the user who is thereby able to control how similar sequences must be in order for their flanking regions to be visualized in the same cluster. The value for the identity threshold value and for the length difference cutoff is defined by a single number between 0.8 and 1, with 1 corresponding to 100%. The following command-line flags were used for CD-HIT-EST: “-M 0 -d 0 -sc 1 g 1 c < user_param> -s < user_param> -n < variable word size>.” The word size flag is automatically defined by Flankophile depending on what the CD-HIT manual defines as appropriate from the choice of identity threshold and length difference cutoff value. The clustering results are used to split all hits included in the flanking region analysis into clusters based on the cluster of their closest matching reference gene. Seqkit (19) “grep” is used to transfer the hit sequences and corresponding flanking sequences to FASTA files for each cluster.

### Flankophile: annotation of flanking regions

The Seqkit (19) command “translate” is used to translate all sequences in the user-defined reference database in order to prepare a custom database for annotation. For each cluster, all the flanking sequences are annotated with Prokka (23) version 1.14.6. The command-line flag “—proteins” is used to annotate first with the custom database. After annotating with the custom database, Prokka annotates insertion sequence transposases from the ISfinder database (24), NCBI Bacterial Antimicrobial Resistance Reference Gene Database (25), and UniProtKB (26) (SwissProt). The Prokka results are combined with the ABRicate results to form annotation tables for later gene synteny plotting with R.

### Flankophile: calculation of distance matrices

KMA (27) command “index” is used on each cluster to index the hit sequences, the flanking sequences, and the combined sequences using a k-mer size defined by the user, with *k* = 6 as the minimum. Then KMA (27) command “dist” is used to calculate distance matrices on the sequences in each cluster using a user-defined distance measure. The user can choose between Jaccard distance, k-mer hamming distance, cosine distance, and chi-square distance. Gene clusters where only a single sequence satisfies the flank requirements is not compared to anything but is still retained for other types of output. Distance calculations between flanking regions and finding of encoded features are entirely independent processes, both working on the contig nucleotides. Therefore, no flanking features need to be identified for clustering and dendrogram formation.

### Flankophile: distance trees and visualization of flanking region plots

The last step of Flankophile is visualization of the flanking regions of the hits in each cluster. R (28) version 4.1.3 is used with the packages Tidyverse (29) version 1.3.2, ggtree (30) version 3.2.0, gggenes (31) version 0.4.1, treeio (32) version 1.18.0, ape (33) version 5.6, and ggnewscale (34) version 0.4.7. The R script constructs distance trees by hierarchical clustering of distance matrices using the “average” (UPGMA) method. This can only occur in gene clusters with >1 flanked variant sequences. For each cluster, three plots are created featuring the distance trees based on the flanking regions alone, the hit sequence alone, and the combined sequence, respectively. The distance trees are combined with heatmap bars, indicating the exact sequence variant of the hit corresponding to each tree tip along with metadata corresponding to the sample if it was supplied by the user. The right side of the plots visualizes the gene/sequence annotations corresponding to each tree tip as colored arrows where the “TARGET” arrow symbolizes the user-specified sequence. Clusters with just a single hit will not get a dendrogram but still get a synteny figure. Figures are output as vector graphics in pdf format, alongside result tables for all hits as well as the hits and annotation tables for each cluster in the flanking region analysis.

### Use case: evaluation data set

We applied Flankophile to a data set consisting of whole-genome-sequenced isolates and metagenomes from humans and pigs in Denmark to compare the gene synteny of acquired resistance genes found in them. We decided to include only sequences from samples that were collected within a 10-year period: 2012–2021. An exhaustive search for all suitable data from that period was not conducted. A total of five data sets from ENA (the European Nucleotide Archive) (35) were used for the study (Table 1).

The first data set is the entire One Day in Denmark collection, which consists of all single isolate clinical samples from Danish patients from a single day in 2018 (36, 37). The second data set is the entire Danish porcine subset of the EFFORT project’s collection of isolated *E. coli* genomes (38) where samples were obtained from cecum of Danish pigs at slaughter. The third data set is the entire Danish porcine subset of the EFFORT project’s fecal-derived metagenome samples (39). The fourth data set is pig fecal metagenomes from Danish VETII project (40) and consists of 210 sequence runs for which we ran metagenomic assembly. The fifth data set is the 2012–2021 subset of the Danmap 2 (Danish Integrated Antimicrobial Resistance Monitoring and Research Programme) collection (41) of fecal-derived metagenomes from pigs. The complete list of sequencing reads, taxonomic origin, and their ENA (35) identifiers can be found in Table S1.

### Use case: reference database

As reference database, we used the ResFinder (9, 10) database of acquired resistance genes which contains 3,144 unique ARGs (version of 31 May 2022). The FASTA file for Flankophile was made by combining the FASTA files for all antibiotic classes and removing duplicates of the sequences that were found in multiple classes.

### Use case: bioinformatic analyses with Flankophile

The single isolate data sets were first assembled with SPAdes (42), and the metagenomes were assembled with meta-SPAdes (43). Paths to the assembly FASTA files along with metadata were given in the input list for Flankophile along with metadata on host. For the Flankophile evaluation run, we chose the following settings in the config file: We required minimum coverage and identity percentages of 99%. A high threshold of 99% for both was used to decrease the risk of including hits that did not confer resistance. A minimum upstream and downstream flank length of 1,500 bp was used since this length allowed space for around two to three genes on each flanking region while also not excluding too much data due to short contigs length in many of the metagenome assemblies. The clustering threshold used for both percent identity and maximum length differences was set to 98%, which allowed very similar reference sequences to be presented in the same cluster. A k-mer size of 16 was chosen for indexing, and Jaccard distance was used as the method for calculating the distance matrix. Flankophile was run on a Linux server with 180 GB memory and 40 CPU cores.

### Use case: processing of results from Flankophile

The result tables from Flankophile along with the AMR class information from the ResFinder database were analyzed using the R (28) version 4.1.2 and the R package Tidyverse (29) version 1.3.1 to produce the results (Fig. 2 to 4). The gene synteny plots were made directly by Flankophile. To reduce the width of the plot subsets in Fig. 5 and 6, the plots have been modified slightly by removing white space between the tree, heatmap, and annotation block. The full plots shown in Fig. S1 have not been altered.

## AFTER EPUB

*[This article was published on 12 December 2023 with with incomplete information in Fig. 2 and 3. The figures were updated in the current version, posted on 20 December 2023.]*

## SUPPLEMENTAL MATERIAL

Figure S1 and Table S1 spectrum.02413-23-s0001.pdf Figure S1 contains 7 gene synteny plots in full size from Flankophile. Table S1 contains information related to the ENA sequences used in the study.

ASM does not own the copyrights to Supplemental Material that may be linked to, or accessed through, an article. The authors have granted ASM a non-exclusive, world-wide license to publish the Supplemental Material files. Please contact the corresponding author directly for reuse.
