# Supplementary material for: Flankophile: a bioinformatic pipeline for prokaryotic genomic synteny analysis
Source: Microbiol Spectr. 2023 Dec 12;12(1):e02413-23. doi: 10.1128/spectrum.02413-23 (PMC10783016; doi:10.1128/spectrum.02413-23)
Supplement: Figure S1 and Table S1 — Figure S1 contains 7 gene synteny plots in full size from Flankophile. Table S1 contains information related to the ENA sequences used in the study. [file spectrum.02413-23-s0001.pdf]

Supplementary Figure 1. Seven gene synteny plots from Flankophile in full size. Each is found on a separate page as they are produced by Flankophile. See Figure 5 for details on annotation and see Table S1 for dataset abbreviations.

Cluster 1_blaTEM_57_1_FJ405211 − distance tree based on flanking region sequences only

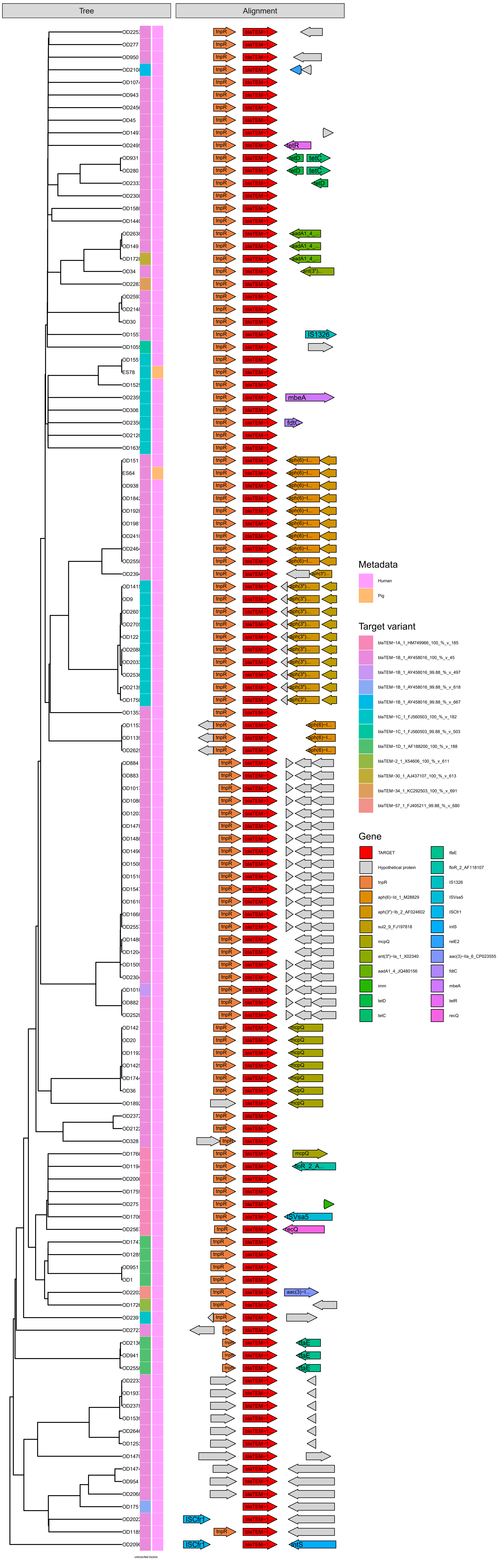

Cluster 27_aph_3____Ib_2_AF024602 − distance tree based on flanking region sequences only

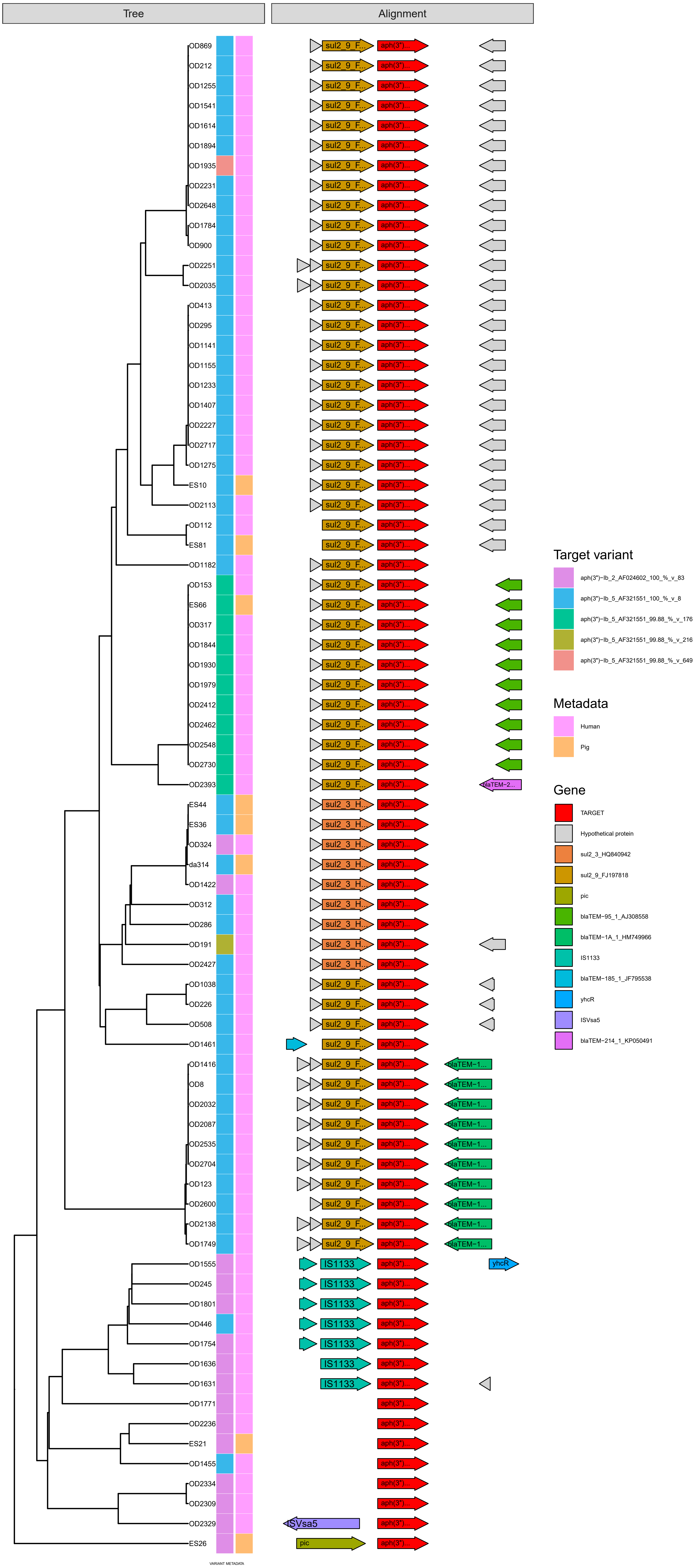

Cluster 28_sul2_2_AY034138 − distance tree based on flanking region sequences only

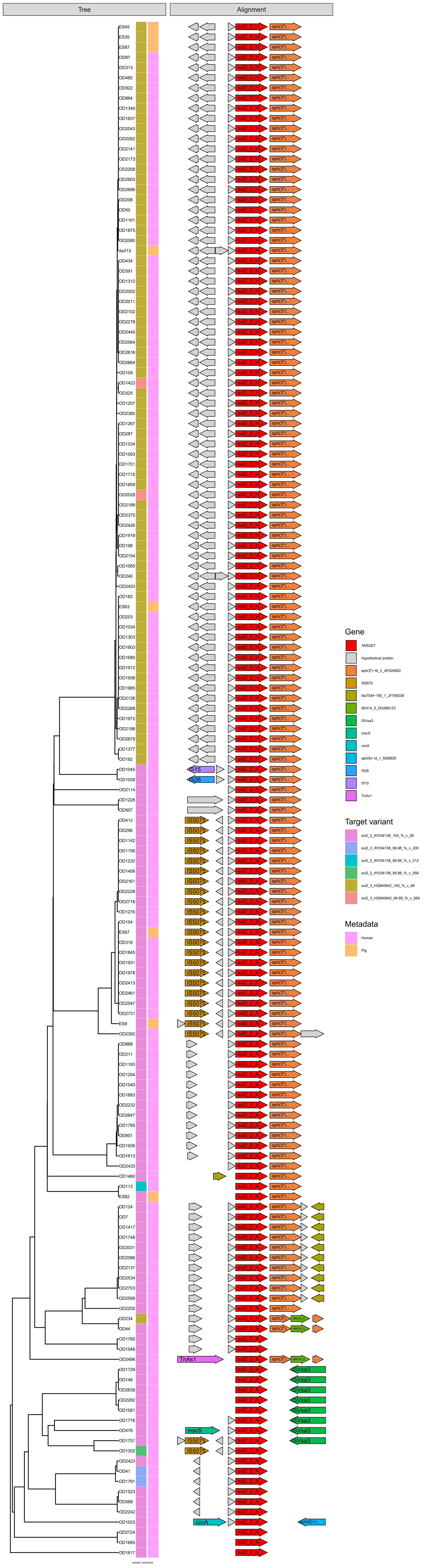

Cluster 58_aph_3___III_1_M26832 − distance tree based on flanking region sequences only

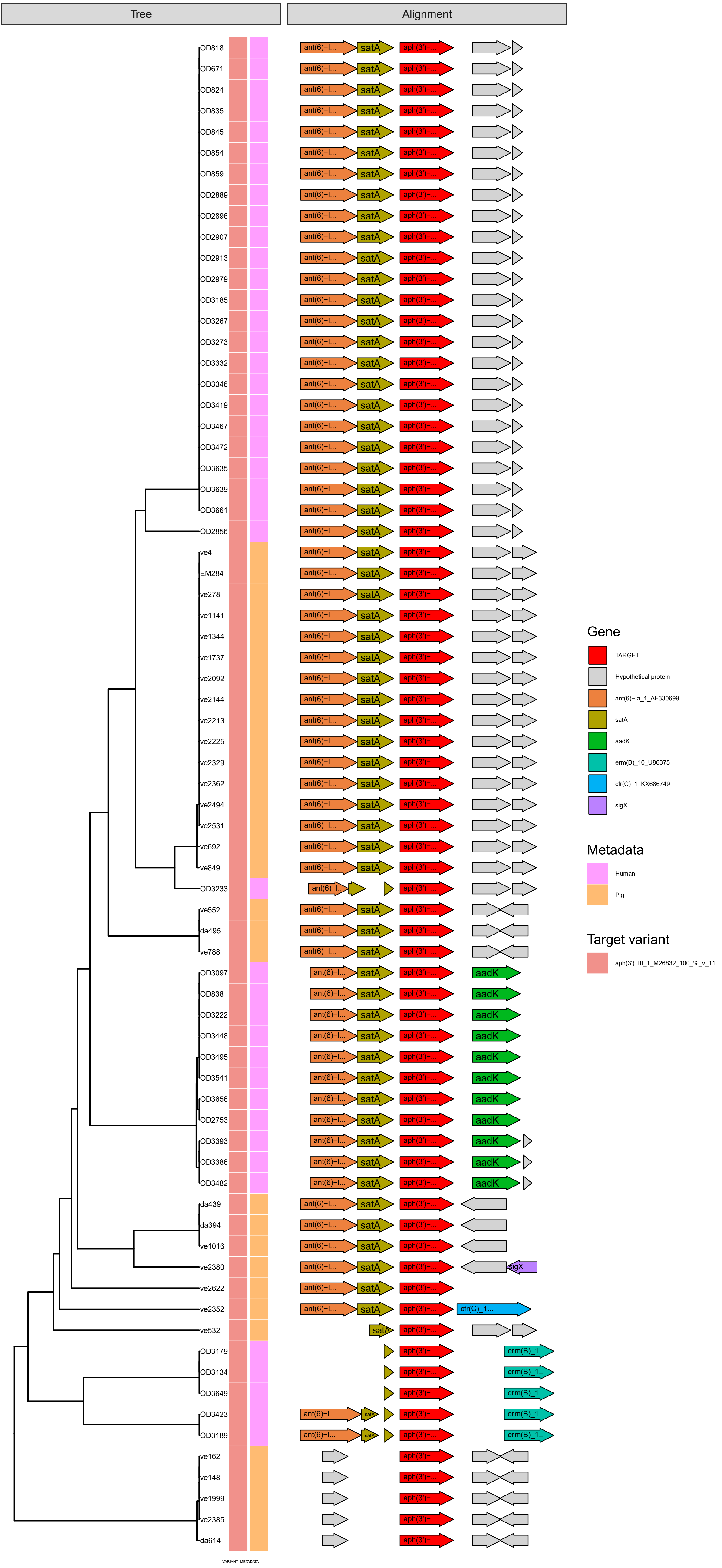

Cluster 132_sul1_5_EU780013 − distance tree based on flanking region sequences only

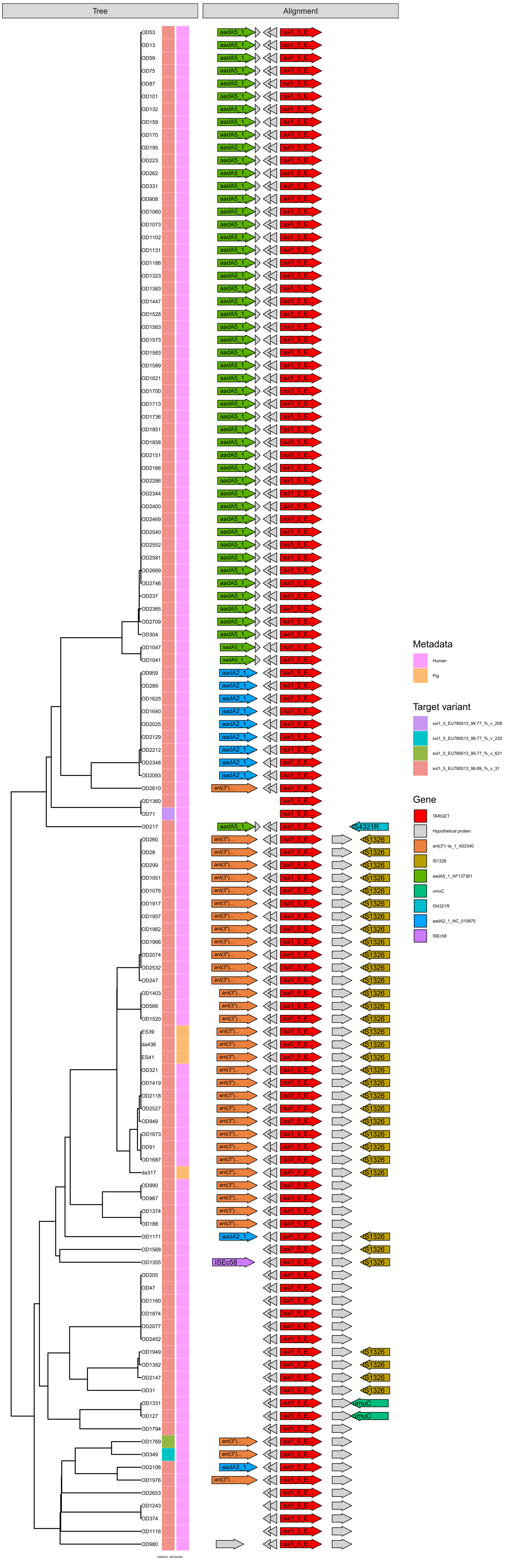

Cluster 140_aph_6__Id_1_M28829 − distance tree based on flanking region sequences only

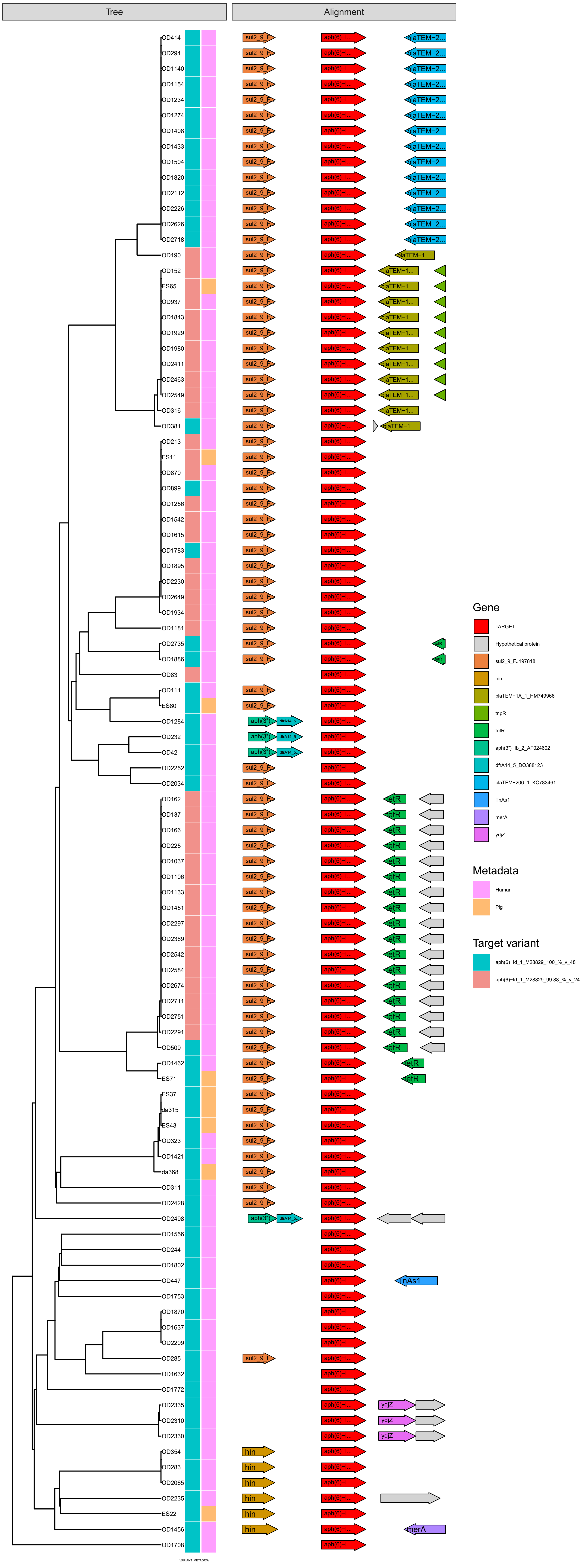

Cluster 162_ant_6__Ia_1_AF330699 − distance tree based on flanking region sequences only

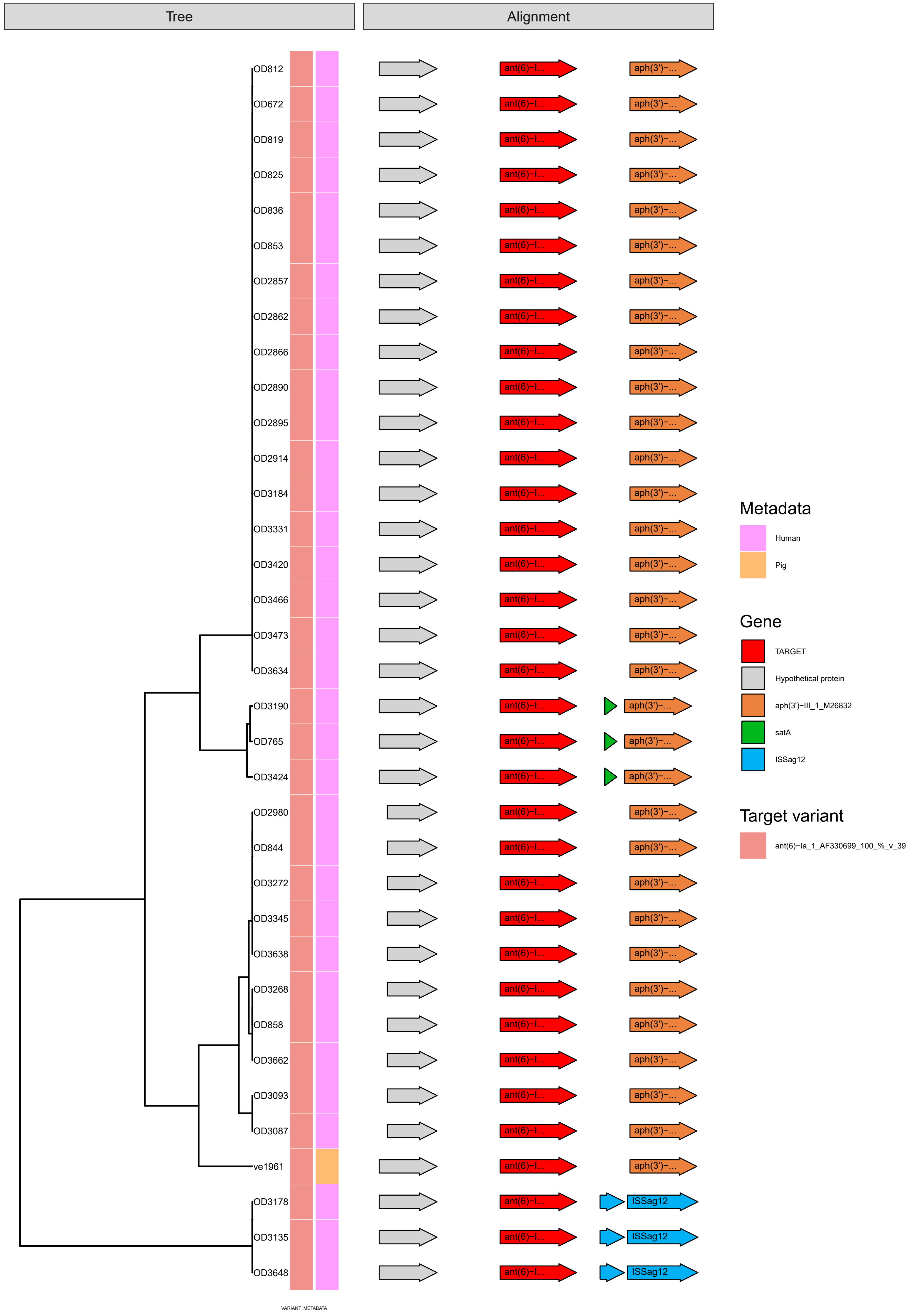

Supplementary Table 1. Table of all the datasets used in this study. Each sequence dataset is annotated with metadata and ENA sequence run accession numbers. The Data Type column abbreviations are the following; MG: metagenome, SI: single isolation (genome).

Table S1

Data set	Host	Year	Study accession ENA	Sample accession ENA	Run accession ENA	Scientific name ENA	Data Type	Data Source
effort_MG	Pig	2015	PRJEB22062	SAMEA104467411	ERR2241908	metagenome	MG	Faecal_samples
effort_MG	Pig	2015	PRJEB22062	SAMEA104467413	ERR2241910	metagenome	MG	Faecal_samples
effort_MG	Pig	2015	PRJEB22062	SAMEA104467415	ERR2241912	metagenome	MG	Faecal_samples
effort_MG	Pig	2015	PRJEB22062	SAMEA104467417	ERR2241914	metagenome	MG	Faecal_samples
effort_MG	Pig	2015	PRJEB22062	SAMEA104467419	ERR2241916	metagenome	MG	Faecal_samples
effort_MG	Pig	2015	PRJEB22062	SAMEA104467421	ERR2241918	metagenome	MG	Faecal_samples
effort_MG	Pig	2015	PRJEB22062	SAMEA104467423	ERR2241920	metagenome	MG	Faecal_samples
effort_MG	Pig	2015	PRJEB22062	SAMEA104467425	ERR2241922	metagenome	MG	Faecal_samples
effort_MG	Pig	2015	PRJEB22062	SAMEA104467427	ERR2241924	metagenome	MG	Faecal_samples
effort_MG	Pig	2015	PRJEB22062	SAMEA104467429	ERR2241926	metagenome	MG	Faecal_samples
effort_MG	Pig	2015	PRJEB22062	SAMEA104467429	ERR2245490	metagenome	MG	Faecal_samples
effort_MG	Pig	2015	PRJEB22062	SAMEA104467431	ERR2241928	metagenome	MG	Faecal_samples
effort_MG	Pig	2015	PRJEB22062	SAMEA104467433	ERR2241930	metagenome	MG	Faecal_samples
effort_MG	Pig	2015	PRJEB22062	SAMEA104467435	ERR2241932	metagenome	MG	Faecal_samples
effort_MG	Pig	2015	PRJEB22062	SAMEA104467437	ERR2241934	metagenome	MG	Faecal_samples
effort_MG	Pig	2015	PRJEB22062	SAMEA104467439	ERR2241936	metagenome	MG	Faecal_samples
effort_MG	Pig	2015	PRJEB22062	SAMEA104467441	ERR2241938	metagenome	MG	Faecal_samples
effort_MG	Pig	2015	PRJEB22062	SAMEA104467443	ERR2241940	metagenome	MG	Faecal_samples
effort_MG	Pig	2015	PRJEB22062	SAMEA104467445	ERR2241942	metagenome	MG	Faecal_samples
effort_MG	Pig	2015	PRJEB22062	SAMEA104467447	ERR2241944	metagenome	MG	Faecal_samples
effort_MG	Pig	2015	PRJEB22062	SAMEA104467449	ERR2241946	metagenome	MG	Faecal_samples
effort_SI	Pig	2015	PRJEB41365	SAMEA7578124	ERR4843771	Escherichia coli	SI	Faecal_samples
effort_SI	Pig	2015	PRJEB41365	SAMEA7578125	ERR4843772	Escherichia coli	SI	Faecal_samples
effort_SI	Pig	2015	PRJEB41365	SAMEA7578126	ERR4843773	Escherichia coli	SI	Faecal_samples
effort_SI	Pig	2015	PRJEB41365	SAMEA7578127	ERR4843774	Escherichia coli	SI	Faecal_samples
effort_SI	Pig	2015	PRJEB41365	SAMEA7578128	ERR4843775	Escherichia coli	SI	Faecal_samples
effort_SI	Pig	2015	PRJEB41365	SAMEA7578129	ERR4843776	Escherichia coli	SI	Faecal_samples
effort_SI	Pig	2015	PRJEB41365	SAMEA7578130	ERR4843777	Escherichia coli	SI	Faecal_samples
effort_SI	Pig	2015	PRJEB41365	SAMEA7578131	ERR4843778	Escherichia coli	SI	Faecal_samples
effort_SI	Pig	2015	PRJEB41365	SAMEA7578132	ERR4843779	Escherichia coli	SI	Faecal_samples
effort_SI	Pig	2015	PRJEB41365	SAMEA7578133	ERR4843780	Escherichia coli	SI	Faecal_samples
effort_SI	Pig	2015	PRJEB41365	SAMEA7578134	ERR4843781	Escherichia coli	SI	Faecal_samples

effort_SI	Pig	2015	PRJEB41365	SAMEA7578135	ERR4843782	Escherichia coli	SI	Faecal_samples
effort_SI	Pig	2015	PRJEB41365	SAMEA7578136	ERR4843783	Escherichia coli	SI	Faecal_samples
effort_SI	Pig	2015	PRJEB41365	SAMEA7578137	ERR4843784	Escherichia coli	SI	Faecal_samples
effort_SI	Pig	2015	PRJEB41365	SAMEA7578138	ERR4843785	Escherichia coli	SI	Faecal_samples
effort_SI	Pig	2015	PRJEB41365	SAMEA7578139	ERR4843786	Escherichia coli	SI	Faecal_samples
effort_SI	Pig	2015	PRJEB41365	SAMEA7578140	ERR4843787	Escherichia coli	SI	Faecal_samples
effort_SI	Pig	2015	PRJEB41365	SAMEA7578141	ERR4843788	Escherichia coli	SI	Faecal_samples
effort_SI	Pig	2015	PRJEB41365	SAMEA7578142	ERR4843789	Escherichia coli	SI	Faecal_samples
effort_SI	Pig	2015	PRJEB41365	SAMEA7578143	ERR4843790	Escherichia coli	SI	Faecal_samples
effort_SI	Pig	2015	PRJEB41365	SAMEA7578144	ERR4843791	Escherichia coli	SI	Faecal_samples
effort_SI	Pig	2015	PRJEB41365	SAMEA7578145	ERR4843792	Escherichia coli	SI	Faecal_samples
effort_SI	Pig	2015	PRJEB41365	SAMEA7578146	ERR4843793	Escherichia coli	SI	Faecal_samples
effort_SI	Pig	2015	PRJEB41365	SAMEA7578147	ERR4843794	Escherichia coli	SI	Faecal_samples
effort_SI	Pig	2015	PRJEB41365	SAMEA7578148	ERR4843795	Escherichia coli	SI	Faecal_samples
effort_SI	Pig	2015	PRJEB41365	SAMEA7578149	ERR4843796	Escherichia coli	SI	Faecal_samples
effort_SI	Pig	2015	PRJEB41365	SAMEA7578150	ERR4843797	Escherichia coli	SI	Faecal_samples
effort_SI	Pig	2015	PRJEB41365	SAMEA7578151	ERR4843798	Escherichia coli	SI	Faecal_samples
effort_SI	Pig	2015	PRJEB41365	SAMEA7578152	ERR4843799	Escherichia coli	SI	Faecal_samples
effort_SI	Pig	2015	PRJEB41365	SAMEA7578153	ERR4843800	Escherichia coli	SI	Faecal_samples
effort_SI	Pig	2015	PRJEB41365	SAMEA7578154	ERR4843801	Escherichia coli	SI	Faecal_samples
effort_SI	Pig	2015	PRJEB41365	SAMEA7578155	ERR4843802	Escherichia coli	SI	Faecal_samples
effort_SI	Pig	2015	PRJEB41365	SAMEA7578156	ERR4843803	Escherichia coli	SI	Faecal_samples
effort_SI	Pig	2015	PRJEB41365	SAMEA7578157	ERR4843804	Escherichia coli	SI	Faecal_samples
effort_SI	Pig	2015	PRJEB41365	SAMEA7578158	ERR4843805	Escherichia coli	SI	Faecal_samples
effort_SI	Pig	2015	PRJEB41365	SAMEA7578159	ERR4843806	Escherichia coli	SI	Faecal_samples
effort_SI	Pig	2015	PRJEB41365	SAMEA7578160	ERR4843807	Escherichia coli	SI	Faecal_samples
effort_SI	Pig	2015	PRJEB41365	SAMEA7578161	ERR4843808	Escherichia coli	SI	Faecal_samples
effort_SI	Pig	2015	PRJEB41365	SAMEA7578162	ERR4843809	Escherichia coli	SI	Faecal_samples
effort_SI	Pig	2015	PRJEB41365	SAMEA7578163	ERR4843810	Escherichia coli	SI	Faecal_samples
effort_SI	Pig	2015	PRJEB41365	SAMEA7578164	ERR4843811	Escherichia coli	SI	Faecal_samples
effort_SI	Pig	2015	PRJEB41365	SAMEA7578165	ERR4843812	Escherichia coli	SI	Faecal_samples
effort_SI	Pig	2015	PRJEB41365	SAMEA7578166	ERR4843813	Escherichia coli	SI	Faecal_samples
effort_SI	Pig	2015	PRJEB41365	SAMEA7578167	ERR4843814	Escherichia coli	SI	Faecal_samples
effort_SI	Pig	2015	PRJEB41365	SAMEA7578168	ERR4843815	Escherichia coli	SI	Faecal_samples

effort_SI	Pig	2015	PRJEB41365	SAMEA7578169	ERR4843816	Escherichia coli	SI	Faecal_samples
effort_SI	Pig	2015	PRJEB41365	SAMEA7578170	ERR4843817	Escherichia coli	SI	Faecal_samples
effort_SI	Pig	2015	PRJEB41365	SAMEA7578171	ERR4843818	Escherichia coli	SI	Faecal_samples
effort_SI	Pig	2015	PRJEB41365	SAMEA7578172	ERR4843819	Escherichia coli	SI	Faecal_samples
effort_SI	Pig	2015	PRJEB41365	SAMEA7578173	ERR4843820	Escherichia coli	SI	Faecal_samples
effort_SI	Pig	2015	PRJEB41365	SAMEA7578174	ERR4843821	Escherichia coli	SI	Faecal_samples
One Day In Denmark	Human	2018	PRJEB37711	SAMEA6656320	ERR4013380	Escherichia coli	SI	Clinical_samples
One Day In Denmark	Human	2018	PRJEB37711	SAMEA6656321	ERR4013381	Escherichia coli	SI	Clinical_samples
One Day In Denmark	Human	2018	PRJEB37711	SAMEA6656322	ERR4013382	Escherichia coli	SI	Clinical_samples
One Day In Denmark	Human	2018	PRJEB37711	SAMEA6656323	ERR4013383	Escherichia coli	SI	Clinical_samples
One Day In Denmark	Human	2018	PRJEB37711	SAMEA6656324	ERR4013384	Escherichia coli	SI	Clinical_samples
One Day In Denmark	Human	2018	PRJEB37711	SAMEA6656325	ERR4013385	Escherichia coli	SI	Clinical_samples
One Day In Denmark	Human	2018	PRJEB37711	SAMEA6656326	ERR4013386	Escherichia coli	SI	Clinical_samples
One Day In Denmark	Human	2018	PRJEB37711	SAMEA6656327	ERR4013387	Escherichia coli	SI	Clinical_samples
One Day In Denmark	Human	2018	PRJEB37711	SAMEA6656328	ERR4013388	Escherichia coli	SI	Clinical_samples
One Day In Denmark	Human	2018	PRJEB37711	SAMEA6656329	ERR4013389	Escherichia coli	SI	Clinical_samples
One Day In Denmark	Human	2018	PRJEB37711	SAMEA6656330	ERR4013390	Escherichia coli	SI	Clinical_samples
One Day In Denmark	Human	2018	PRJEB37711	SAMEA6656331	ERR4013391	Escherichia coli	SI	Clinical_samples
One Day In Denmark	Human	2018	PRJEB37711	SAMEA6656332	ERR4013392	Aerococcus urinae	SI	Clinical_samples
One Day In Denmark	Human	2018	PRJEB37711	SAMEA6656333	ERR4013393	Escherichia coli	SI	Clinical_samples
One Day In Denmark	Human	2018	PRJEB37711	SAMEA6656334	ERR4013394	Escherichia coli	SI	Clinical_samples
One Day In Denmark	Human	2018	PRJEB37711	SAMEA6656335	ERR4013395	Enterococcus faecalis	SI	Clinical_samples
One Day In Denmark	Human	2018	PRJEB37711	SAMEA6656336	ERR4013396	Escherichia coli	SI	Clinical_samples
One Day In Denmark	Human	2018	PRJEB37711	SAMEA6656337	ERR4013397	Escherichia coli	SI	Clinical_samples
One Day In Denmark	Human	2018	PRJEB37711	SAMEA6656338	ERR4013398	Escherichia coli	SI	Clinical_samples
One Day In Denmark	Human	2018	PRJEB37711	SAMEA6656339	ERR4013399	Escherichia coli	SI	Clinical_samples
One Day In Denmark	Human	2018	PRJEB37711	SAMEA6656340	ERR4013400	Escherichia coli	SI	Clinical_samples
One Day In Denmark	Human	2018	PRJEB37711	SAMEA6656341	ERR4013401	Escherichia coli	SI	Clinical_samples
One Day In Denmark	Human	2018	PRJEB37711	SAMEA6656342	ERR4013402	Escherichia coli	SI	Clinical_samples
One Day In Denmark	Human	2018	PRJEB37711	SAMEA6656343	ERR4013403	Escherichia coli	SI	Clinical_samples
One Day In Denmark	Human	2018	PRJEB37711	SAMEA6656344	ERR4013404	Escherichia coli	SI	Clinical_samples
One Day In Denmark	Human	2018	PRJEB37711	SAMEA6656345	ERR4013405	Escherichia coli	SI	Clinical_samples
One Day In Denmark	Human	2018	PRJEB37711	SAMEA6656346	ERR4013406	Escherichia coli	SI	Clinical_samples
One Day In Denmark	Human	2018	PRJEB37711	SAMEA6656347	ERR4013407	Escherichia coli	SI	Clinical_samples

One Day In Denmark	Human	2018	PRJEB37711	SAMEA6656348	ERR4013408	Escherichia coli	SI	Clinical_samples
One Day In Denmark	Human	2018	PRJEB37711	SAMEA6656349	ERR4013409	Escherichia coli	SI	Clinical_samples
One Day In Denmark	Human	2018	PRJEB37711	SAMEA6656350	ERR4013410	Escherichia coli	SI	Clinical_samples
One Day In Denmark	Human	2018	PRJEB37711	SAMEA6656351	ERR4013411	Escherichia coli	SI	Clinical_samples
One Day In Denmark	Human	2018	PRJEB37711	SAMEA6656352	ERR4013412	Escherichia coli	SI	Clinical_samples
One Day In Denmark	Human	2018	PRJEB37711	SAMEA6656353	ERR4013413	Escherichia coli	SI	Clinical_samples
One Day In Denmark	Human	2018	PRJEB37711	SAMEA6656354	ERR4013414	Escherichia coli	SI	Clinical_samples
One Day In Denmark	Human	2018	PRJEB37711	SAMEA6656355	ERR4013415	Escherichia coli	SI	Clinical_samples
One Day In Denmark	Human	2018	PRJEB37711	SAMEA6656356	ERR4013416	Escherichia coli	SI	Clinical_samples
One Day In Denmark	Human	2018	PRJEB37711	SAMEA6656357	ERR4013417	Escherichia coli	SI	Clinical_samples
One Day In Denmark	Human	2018	PRJEB37711	SAMEA6656358	ERR4013418	Escherichia coli	SI	Clinical_samples
One Day In Denmark	Human	2018	PRJEB37711	SAMEA6656359	ERR4013419	Escherichia coli	SI	Clinical_samples
One Day In Denmark	Human	2018	PRJEB37711	SAMEA6656360	ERR4013420	Escherichia coli	SI	Clinical_samples
One Day In Denmark	Human	2018	PRJEB37711	SAMEA6656361	ERR4013421	Escherichia coli	SI	Clinical_samples
One Day In Denmark	Human	2018	PRJEB37711	SAMEA6656362	ERR4013422	Escherichia coli	SI	Clinical_samples
One Day In Denmark	Human	2018	PRJEB37711	SAMEA6656363	ERR4013423	Escherichia coli	SI	Clinical_samples
One Day In Denmark	Human	2018	PRJEB37711	SAMEA6656364	ERR4013424	Escherichia coli	SI	Clinical_samples
One Day In Denmark	Human	2018	PRJEB37711	SAMEA6656365	ERR4013425	Escherichia coli	SI	Clinical_samples
One Day In Denmark	Human	2018	PRJEB37711	SAMEA6656366	ERR4013426	Escherichia coli	SI	Clinical_samples
One Day In Denmark	Human	2018	PRJEB37711	SAMEA6656367	ERR4013427	Escherichia coli	SI	Clinical_samples
One Day In Denmark	Human	2018	PRJEB37711	SAMEA6656368	ERR4013428	Escherichia coli	SI	Clinical_samples
One Day In Denmark	Human	2018	PRJEB37711	SAMEA6656369	ERR4013429	Escherichia coli	SI	Clinical_samples
One Day In Denmark	Human	2018	PRJEB37711	SAMEA6656370	ERR4013430	Escherichia coli	SI	Clinical_samples
One Day In Denmark	Human	2018	PRJEB37711	SAMEA6656371	ERR4013431	Escherichia coli	SI	Clinical_samples
One Day In Denmark	Human	2018	PRJEB37711	SAMEA6656372	ERR4013432	Escherichia coli	SI	Clinical_samples
One Day In Denmark	Human	2018	PRJEB37711	SAMEA6656373	ERR4013433	Escherichia coli	SI	Clinical_samples
One Day In Denmark	Human	2018	PRJEB37711	SAMEA6656374	ERR4013434	Escherichia coli	SI	Clinical_samples
One Day In Denmark	Human	2018	PRJEB37711	SAMEA6656375	ERR4013435	Escherichia coli	SI	Clinical_samples
One Day In Denmark	Human	2018	PRJEB37711	SAMEA6656376	ERR4013436	Escherichia coli	SI	Clinical_samples
One Day In Denmark	Human	2018	PRJEB37711	SAMEA6656377	ERR4013437	Escherichia coli	SI	Clinical_samples
One Day In Denmark	Human	2018	PRJEB37711	SAMEA6656378	ERR4013438	Escherichia coli	SI	Clinical_samples
One Day In Denmark	Human	2018	PRJEB37711	SAMEA6656379	ERR4013439	Escherichia coli	SI	Clinical_samples
One Day In Denmark	Human	2018	PRJEB37711	SAMEA6656380	ERR4013440	Escherichia coli	SI	Clinical_samples
One Day In Denmark	Human	2018	PRJEB37711	SAMEA6656381	ERR4013441	Escherichia coli	SI	Clinical_samples

One Day In Denmark	Human	2018	PRJEB37711	SAMEA6656382	ERR4013442	Escherichia coli	SI	Clinical_samples
One Day In Denmark	Human	2018	PRJEB37711	SAMEA6656383	ERR4013443	Escherichia coli	SI	Clinical_samples
One Day In Denmark	Human	2018	PRJEB37711	SAMEA6656384	ERR4013444	Escherichia coli	SI	Clinical_samples
One Day In Denmark	Human	2018	PRJEB37711	SAMEA6656385	ERR4013445	Escherichia coli	SI	Clinical_samples
One Day In Denmark	Human	2018	PRJEB37711	SAMEA6656386	ERR4013446	Escherichia coli	SI	Clinical_samples
One Day In Denmark	Human	2018	PRJEB37711	SAMEA6656387	ERR4013447	Escherichia coli	SI	Clinical_samples
One Day In Denmark	Human	2018	PRJEB37711	SAMEA6656388	ERR4013448	Escherichia coli	SI	Clinical_samples
One Day In Denmark	Human	2018	PRJEB37711	SAMEA6656389	ERR4013449	Escherichia coli	SI	Clinical_samples
One Day In Denmark	Human	2018	PRJEB37711	SAMEA6656390	ERR4013450	Escherichia coli	SI	Clinical_samples
One Day In Denmark	Human	2018	PRJEB37711	SAMEA6656391	ERR4013451	Escherichia coli	SI	Clinical_samples
One Day In Denmark	Human	2018	PRJEB37711	SAMEA6656392	ERR4013452	Escherichia coli	SI	Clinical_samples
One Day In Denmark	Human	2018	PRJEB37711	SAMEA6656393	ERR4013453	Escherichia coli	SI	Clinical_samples
One Day In Denmark	Human	2018	PRJEB37711	SAMEA6656394	ERR4013454	Escherichia coli	SI	Clinical_samples
One Day In Denmark	Human	2018	PRJEB37711	SAMEA6656395	ERR4013455	Escherichia coli	SI	Clinical_samples
One Day In Denmark	Human	2018	PRJEB37711	SAMEA6656396	ERR4013456	Escherichia coli	SI	Clinical_samples
One Day In Denmark	Human	2018	PRJEB37711	SAMEA6656397	ERR4013457	Escherichia coli	SI	Clinical_samples
One Day In Denmark	Human	2018	PRJEB37711	SAMEA6656398	ERR4013458	Escherichia coli	SI	Clinical_samples
One Day In Denmark	Human	2018	PRJEB37711	SAMEA6656399	ERR4013459	Escherichia coli	SI	Clinical_samples
One Day In Denmark	Human	2018	PRJEB37711	SAMEA6656400	ERR4013460	Escherichia coli	SI	Clinical_samples
One Day In Denmark	Human	2018	PRJEB37711	SAMEA6656401	ERR4013461	Escherichia coli	SI	Clinical_samples
One Day In Denmark	Human	2018	PRJEB37711	SAMEA6656402	ERR4013462	Escherichia coli	SI	Clinical_samples
One Day In Denmark	Human	2018	PRJEB37711	SAMEA6656403	ERR4013463	Escherichia coli	SI	Clinical_samples
One Day In Denmark	Human	2018	PRJEB37711	SAMEA6656404	ERR4013464	Escherichia coli	SI	Clinical_samples
One Day In Denmark	Human	2018	PRJEB37711	SAMEA6656405	ERR4013465	Escherichia coli	SI	Clinical_samples
One Day In Denmark	Human	2018	PRJEB37711	SAMEA6656406	ERR4013466	Escherichia coli	SI	Clinical_samples
One Day In Denmark	Human	2018	PRJEB37711	SAMEA6656407	ERR4013467	Escherichia coli	SI	Clinical_samples
One Day In Denmark	Human	2018	PRJEB37711	SAMEA6656408	ERR4013468	Escherichia coli	SI	Clinical_samples
One Day In Denmark	Human	2018	PRJEB37711	SAMEA6656409	ERR4013469	Escherichia coli	SI	Clinical_samples
One Day In Denmark	Human	2018	PRJEB37711	SAMEA6656410	ERR4013470	Escherichia coli	SI	Clinical_samples
One Day In Denmark	Human	2018	PRJEB37711	SAMEA6656411	ERR4013471	Escherichia coli	SI	Clinical_samples
One Day In Denmark	Human	2018	PRJEB37711	SAMEA6656412	ERR4013472	Escherichia coli	SI	Clinical_samples
One Day In Denmark	Human	2018	PRJEB37711	SAMEA6656413	ERR4013473	Escherichia coli	SI	Clinical_samples
One Day In Denmark	Human	2018	PRJEB37711	SAMEA6656414	ERR4013474	Escherichia coli	SI	Clinical_samples
One Day In Denmark	Human	2018	PRJEB37711	SAMEA6656415	ERR4013475	Escherichia coli	SI	Clinical_samples

One Day In Denmark	Human	2018	PRJEB37711	SAMEA6656416	ERR4013476	Escherichia coli	SI	Clinical_samples
One Day In Denmark	Human	2018	PRJEB37711	SAMEA6656417	ERR4013477	Escherichia coli	SI	Clinical_samples
One Day In Denmark	Human	2018	PRJEB37711	SAMEA6656418	ERR4013478	Escherichia coli	SI	Clinical_samples
One Day In Denmark	Human	2018	PRJEB37711	SAMEA6656419	ERR4013479	Escherichia coli	SI	Clinical_samples
One Day In Denmark	Human	2018	PRJEB37711	SAMEA6656420	ERR4013480	Escherichia coli	SI	Clinical_samples
One Day In Denmark	Human	2018	PRJEB37711	SAMEA6656421	ERR4013481	Escherichia coli	SI	Clinical_samples
One Day In Denmark	Human	2018	PRJEB37711	SAMEA6656422	ERR4013482	Escherichia coli	SI	Clinical_samples
One Day In Denmark	Human	2018	PRJEB37711	SAMEA6656423	ERR4013483	Escherichia coli	SI	Clinical_samples
One Day In Denmark	Human	2018	PRJEB37711	SAMEA6656424	ERR4013484	Escherichia coli	SI	Clinical_samples
One Day In Denmark	Human	2018	PRJEB37711	SAMEA6656425	ERR4013485	Escherichia coli	SI	Clinical_samples
One Day In Denmark	Human	2018	PRJEB37711	SAMEA6656426	ERR4013486	Escherichia coli	SI	Clinical_samples
One Day In Denmark	Human	2018	PRJEB37711	SAMEA6656427	ERR4013487	Escherichia coli	SI	Clinical_samples
One Day In Denmark	Human	2018	PRJEB37711	SAMEA6656428	ERR4013488	Escherichia coli	SI	Clinical_samples
One Day In Denmark	Human	2018	PRJEB37711	SAMEA6656429	ERR4013489	Escherichia coli	SI	Clinical_samples
One Day In Denmark	Human	2018	PRJEB37711	SAMEA6656430	ERR4013490	Escherichia coli	SI	Clinical_samples
One Day In Denmark	Human	2018	PRJEB37711	SAMEA6656431	ERR4013491	Escherichia coli	SI	Clinical_samples
One Day In Denmark	Human	2018	PRJEB37711	SAMEA6656432	ERR4013492	Escherichia coli	SI	Clinical_samples
One Day In Denmark	Human	2018	PRJEB37711	SAMEA6656433	ERR4013493	Escherichia coli	SI	Clinical_samples
One Day In Denmark	Human	2018	PRJEB37711	SAMEA6656434	ERR4013494	Escherichia coli	SI	Clinical_samples
One Day In Denmark	Human	2018	PRJEB37711	SAMEA6656435	ERR4013495	Escherichia coli	SI	Clinical_samples
One Day In Denmark	Human	2018	PRJEB37711	SAMEA6656436	ERR4013496	Escherichia coli	SI	Clinical_samples
One Day In Denmark	Human	2018	PRJEB37711	SAMEA6656437	ERR4013497	Escherichia coli	SI	Clinical_samples
One Day In Denmark	Human	2018	PRJEB37711	SAMEA6656438	ERR4013498	Escherichia coli	SI	Clinical_samples
One Day In Denmark	Human	2018	PRJEB37711	SAMEA6656439	ERR4013499	Escherichia coli	SI	Clinical_samples
One Day In Denmark	Human	2018	PRJEB37711	SAMEA6656440	ERR4013500	Escherichia coli	SI	Clinical_samples
One Day In Denmark	Human	2018	PRJEB37711	SAMEA6656441	ERR4013501	Escherichia coli	SI	Clinical_samples
One Day In Denmark	Human	2018	PRJEB37711	SAMEA6656442	ERR4013502	Escherichia coli	SI	Clinical_samples
One Day In Denmark	Human	2018	PRJEB37711	SAMEA6656443	ERR4013503	Escherichia coli	SI	Clinical_samples
One Day In Denmark	Human	2018	PRJEB37711	SAMEA6656444	ERR4013504	Escherichia coli	SI	Clinical_samples
One Day In Denmark	Human	2018	PRJEB37711	SAMEA6656445	ERR4013505	Escherichia coli	SI	Clinical_samples
One Day In Denmark	Human	2018	PRJEB37711	SAMEA6656446	ERR4013506	Escherichia coli	SI	Clinical_samples
One Day In Denmark	Human	2018	PRJEB37711	SAMEA6656447	ERR4013507	Escherichia coli	SI	Clinical_samples
One Day In Denmark	Human	2018	PRJEB37711	SAMEA6656448	ERR4013508	Escherichia coli	SI	Clinical_samples
One Day In Denmark	Human	2018	PRJEB37711	SAMEA6656449	ERR4013509	Escherichia coli	SI	Clinical_samples

One Day In Denmark	Human	2018	PRJEB37711	SAMEA6656450	ERR4013510	Escherichia coli	SI	Clinical_samples
One Day In Denmark	Human	2018	PRJEB37711	SAMEA6656451	ERR4013511	Escherichia coli	SI	Clinical_samples
One Day In Denmark	Human	2018	PRJEB37711	SAMEA6656452	ERR4013512	Escherichia coli	SI	Clinical_samples
One Day In Denmark	Human	2018	PRJEB37711	SAMEA6656453	ERR4013513	Escherichia coli	SI	Clinical_samples
One Day In Denmark	Human	2018	PRJEB37711	SAMEA6656454	ERR4013514	Escherichia coli	SI	Clinical_samples
One Day In Denmark	Human	2018	PRJEB37711	SAMEA6656455	ERR4013515	Escherichia coli	SI	Clinical_samples
One Day In Denmark	Human	2018	PRJEB37711	SAMEA6656456	ERR4013516	Escherichia coli	SI	Clinical_samples
One Day In Denmark	Human	2018	PRJEB37711	SAMEA6656457	ERR4013517	Escherichia coli	SI	Clinical_samples
One Day In Denmark	Human	2018	PRJEB37711	SAMEA6656458	ERR4013518	Escherichia coli	SI	Clinical_samples
One Day In Denmark	Human	2018	PRJEB37711	SAMEA6656459	ERR4013519	Escherichia coli	SI	Clinical_samples
One Day In Denmark	Human	2018	PRJEB37711	SAMEA6656460	ERR4013520	Escherichia coli	SI	Clinical_samples
One Day In Denmark	Human	2018	PRJEB37711	SAMEA6656461	ERR4013521	Escherichia coli	SI	Clinical_samples
One Day In Denmark	Human	2018	PRJEB37711	SAMEA6656462	ERR4013522	Escherichia coli	SI	Clinical_samples
One Day In Denmark	Human	2018	PRJEB37711	SAMEA6656463	ERR4013523	Escherichia coli	SI	Clinical_samples
One Day In Denmark	Human	2018	PRJEB37711	SAMEA6656464	ERR4013524	Escherichia coli	SI	Clinical_samples
One Day In Denmark	Human	2018	PRJEB37711	SAMEA6656465	ERR4013525	Escherichia coli	SI	Clinical_samples
One Day In Denmark	Human	2018	PRJEB37711	SAMEA6656466	ERR4013526	Escherichia coli	SI	Clinical_samples
One Day In Denmark	Human	2018	PRJEB37711	SAMEA6656467	ERR4013527	Escherichia coli	SI	Clinical_samples
One Day In Denmark	Human	2018	PRJEB37711	SAMEA6656468	ERR4013528	Escherichia coli	SI	Clinical_samples
One Day In Denmark	Human	2018	PRJEB37711	SAMEA6656469	ERR4013529	Escherichia coli	SI	Clinical_samples
One Day In Denmark	Human	2018	PRJEB37711	SAMEA6656470	ERR4013530	Escherichia coli	SI	Clinical_samples
One Day In Denmark	Human	2018	PRJEB37711	SAMEA6656471	ERR4013531	Escherichia coli	SI	Clinical_samples
One Day In Denmark	Human	2018	PRJEB37711	SAMEA6656472	ERR4013532	Escherichia coli	SI	Clinical_samples
One Day In Denmark	Human	2018	PRJEB37711	SAMEA6656473	ERR4013533	Escherichia coli	SI	Clinical_samples
One Day In Denmark	Human	2018	PRJEB37711	SAMEA6656474	ERR4013534	Escherichia coli	SI	Clinical_samples
One Day In Denmark	Human	2018	PRJEB37711	SAMEA6656475	ERR4013535	Escherichia coli	SI	Clinical_samples
One Day In Denmark	Human	2018	PRJEB37711	SAMEA6656476	ERR4013536	Escherichia coli	SI	Clinical_samples
One Day In Denmark	Human	2018	PRJEB37711	SAMEA6656477	ERR4013537	Escherichia coli	SI	Clinical_samples
One Day In Denmark	Human	2018	PRJEB37711	SAMEA6656478	ERR4013538	Escherichia coli	SI	Clinical_samples
One Day In Denmark	Human	2018	PRJEB37711	SAMEA6656479	ERR4013539	Escherichia coli	SI	Clinical_samples
One Day In Denmark	Human	2018	PRJEB37711	SAMEA6656480	ERR4013540	Escherichia coli	SI	Clinical_samples
One Day In Denmark	Human	2018	PRJEB37711	SAMEA6656481	ERR4013541	Escherichia coli	SI	Clinical_samples
One Day In Denmark	Human	2018	PRJEB37711	SAMEA6656482	ERR4013542	Escherichia coli	SI	Clinical_samples
One Day In Denmark	Human	2018	PRJEB37711	SAMEA6656483	ERR4013543	Escherichia coli	SI	Clinical_samples

One Day In Denmark	Human	2018	PRJEB37711	SAMEA6656484	ERR4013544	Escherichia coli	SI	Clinical_samples
One Day In Denmark	Human	2018	PRJEB37711	SAMEA6656485	ERR4013545	Escherichia coli	SI	Clinical_samples
One Day In Denmark	Human	2018	PRJEB37711	SAMEA6656486	ERR4013546	Escherichia coli	SI	Clinical_samples
One Day In Denmark	Human	2018	PRJEB37711	SAMEA6656487	ERR4013547	Escherichia coli	SI	Clinical_samples
One Day In Denmark	Human	2018	PRJEB37711	SAMEA6656488	ERR4013548	Escherichia coli	SI	Clinical_samples
One Day In Denmark	Human	2018	PRJEB37711	SAMEA6656489	ERR4013549	Escherichia coli	SI	Clinical_samples
One Day In Denmark	Human	2018	PRJEB37711	SAMEA6656490	ERR4013550	Escherichia coli	SI	Clinical_samples
One Day In Denmark	Human	2018	PRJEB37711	SAMEA6656491	ERR4013551	Raoultella planticola	SI	Clinical_samples
One Day In Denmark	Human	2018	PRJEB37711	SAMEA6656492	ERR4013552	Klebsiella oxytoca	SI	Clinical_samples
One Day In Denmark	Human	2018	PRJEB37711	SAMEA6656493	ERR4013553	Klebsiella oxytoca	SI	Clinical_samples
One Day In Denmark	Human	2018	PRJEB37711	SAMEA6656494	ERR4013554	Klebsiella oxytoca	SI	Clinical_samples
One Day In Denmark	Human	2018	PRJEB37711	SAMEA6656495	ERR4013555	Klebsiella oxytoca	SI	Clinical_samples
One Day In Denmark	Human	2018	PRJEB37711	SAMEA6656496	ERR4013556	Klebsiella oxytoca	SI	Clinical_samples
One Day In Denmark	Human	2018	PRJEB37711	SAMEA6656497	ERR4013557	Klebsiella oxytoca	SI	Clinical_samples
One Day In Denmark	Human	2018	PRJEB37711	SAMEA6656498	ERR4013558	Klebsiella oxytoca	SI	Clinical_samples
One Day In Denmark	Human	2018	PRJEB37711	SAMEA6656499	ERR4013559	Klebsiella oxytoca	SI	Clinical_samples
One Day In Denmark	Human	2018	PRJEB37711	SAMEA6656500	ERR4013560	Klebsiella oxytoca	SI	Clinical_samples
One Day In Denmark	Human	2018	PRJEB37711	SAMEA6656501	ERR4013561	Klebsiella pneumoniae	SI	Clinical_samples
One Day In Denmark	Human	2018	PRJEB37711	SAMEA6656502	ERR4013562	Klebsiella pneumoniae	SI	Clinical_samples
One Day In Denmark	Human	2018	PRJEB37711	SAMEA6656503	ERR4013563	Klebsiella pneumoniae	SI	Clinical_samples
One Day In Denmark	Human	2018	PRJEB37711	SAMEA6656504	ERR4013564	Klebsiella pneumoniae	SI	Clinical_samples
One Day In Denmark	Human	2018	PRJEB37711	SAMEA6656505	ERR4013565	Klebsiella pneumoniae	SI	Clinical_samples
One Day In Denmark	Human	2018	PRJEB37711	SAMEA6656506	ERR4013566	Klebsiella pneumoniae	SI	Clinical_samples
One Day In Denmark	Human	2018	PRJEB37711	SAMEA6656507	ERR4013567	Klebsiella pneumoniae	SI	Clinical_samples
One Day In Denmark	Human	2018	PRJEB37711	SAMEA6656508	ERR4013568	Klebsiella pneumoniae	SI	Clinical_samples
One Day In Denmark	Human	2018	PRJEB37711	SAMEA6656509	ERR4013569	Klebsiella pneumoniae	SI	Clinical_samples
One Day In Denmark	Human	2018	PRJEB37711	SAMEA6656510	ERR4013570	Klebsiella pneumoniae	SI	Clinical_samples
One Day In Denmark	Human	2018	PRJEB37711	SAMEA6656511	ERR4013571	Klebsiella pneumoniae	SI	Clinical_samples
One Day In Denmark	Human	2018	PRJEB37711	SAMEA6656512	ERR4013572	Klebsiella variicola	SI	Clinical_samples
One Day In Denmark	Human	2018	PRJEB37711	SAMEA6656513	ERR4013573	Klebsiella pneumoniae	SI	Clinical_samples
One Day In Denmark	Human	2018	PRJEB37711	SAMEA6656514	ERR4013574	Klebsiella pneumoniae	SI	Clinical_samples
One Day In Denmark	Human	2018	PRJEB37711	SAMEA6656515	ERR4013575	Klebsiella pneumoniae	SI	Clinical_samples
One Day In Denmark	Human	2018	PRJEB37711	SAMEA6656516	ERR4013576	Klebsiella pneumoniae	SI	Clinical_samples
One Day In Denmark	Human	2018	PRJEB37711	SAMEA6656517	ERR4013577	Klebsiella pneumoniae	SI	Clinical_samples

One Day In Denmark	Human	2018	PRJEB37711	SAMEA6656518	ERR4013578	Klebsiella pneumoniae	SI	Clinical_samples
One Day In Denmark	Human	2018	PRJEB37711	SAMEA6656519	ERR4013579	Klebsiella oxytoca	SI	Clinical_samples
One Day In Denmark	Human	2018	PRJEB37711	SAMEA6656520	ERR4013580	Klebsiella pneumoniae	SI	Clinical_samples
One Day In Denmark	Human	2018	PRJEB37711	SAMEA6656521	ERR4013581	Klebsiella pneumoniae	SI	Clinical_samples
One Day In Denmark	Human	2018	PRJEB37711	SAMEA6656522	ERR4013582	Klebsiella pneumoniae	SI	Clinical_samples
One Day In Denmark	Human	2018	PRJEB37711	SAMEA6656523	ERR4013583	Proteus mirabilis	SI	Clinical_samples
One Day In Denmark	Human	2018	PRJEB37711	SAMEA6656524	ERR4013584	Proteus mirabilis	SI	Clinical_samples
One Day In Denmark	Human	2018	PRJEB37711	SAMEA6656525	ERR4013585	Proteus mirabilis	SI	Clinical_samples
One Day In Denmark	Human	2018	PRJEB37711	SAMEA6656526	ERR4013586	Proteus mirabilis	SI	Clinical_samples
One Day In Denmark	Human	2018	PRJEB37711	SAMEA6656527	ERR4013587	Proteus mirabilis	SI	Clinical_samples
One Day In Denmark	Human	2018	PRJEB37711	SAMEA6656528	ERR4013588	Proteus mirabilis	SI	Clinical_samples
One Day In Denmark	Human	2018	PRJEB37711	SAMEA6656529	ERR4013589	Proteus mirabilis	SI	Clinical_samples
One Day In Denmark	Human	2018	PRJEB37711	SAMEA6656530	ERR4013590	Proteus mirabilis	SI	Clinical_samples
One Day In Denmark	Human	2018	PRJEB37711	SAMEA6656531	ERR4013591	Proteus mirabilis	SI	Clinical_samples
One Day In Denmark	Human	2018	PRJEB37711	SAMEA6656532	ERR4013592	Proteus mirabilis	SI	Clinical_samples
One Day In Denmark	Human	2018	PRJEB37711	SAMEA6656533	ERR4013593	Morganella morganii	SI	Clinical_samples
One Day In Denmark	Human	2018	PRJEB37711	SAMEA6656534	ERR4013594	Citrobacter braakii	SI	Clinical_samples
One Day In Denmark	Human	2018	PRJEB37711	SAMEA6656535	ERR4013595	Enterobacter cloacae	SI	Clinical_samples
One Day In Denmark	Human	2018	PRJEB37711	SAMEA6656536	ERR4013596	Citrobacter braakii	SI	Clinical_samples
One Day In Denmark	Human	2018	PRJEB37711	SAMEA6656537	ERR4013597	Citrobacter freundii	SI	Clinical_samples
One Day In Denmark	Human	2018	PRJEB37711	SAMEA6656538	ERR4013598	Citrobacter koseri	SI	Clinical_samples
One Day In Denmark	Human	2018	PRJEB37711	SAMEA6656539	ERR4013599	Citrobacter koseri	SI	Clinical_samples
One Day In Denmark	Human	2018	PRJEB37711	SAMEA6656540	ERR4013600	Citrobacter koseri	SI	Clinical_samples
One Day In Denmark	Human	2018	PRJEB37711	SAMEA6656541	ERR4013601	Citrobacter koseri	SI	Clinical_samples
One Day In Denmark	Human	2018	PRJEB37711	SAMEA6656542	ERR4013602	Citrobacter koseri	SI	Clinical_samples
One Day In Denmark	Human	2018	PRJEB37711	SAMEA6656543	ERR4013603	Klebsiella aerogenes	SI	Clinical_samples
One Day In Denmark	Human	2018	PRJEB37711	SAMEA6656544	ERR4013604	Klebsiella aerogenes	SI	Clinical_samples
One Day In Denmark	Human	2018	PRJEB37711	SAMEA6656545	ERR4013605	Enterobacter cloacae	SI	Clinical_samples
One Day In Denmark	Human	2018	PRJEB37711	SAMEA6656546	ERR4013606	Enterobacter cloacae	SI	Clinical_samples
One Day In Denmark	Human	2018	PRJEB37711	SAMEA6656547	ERR4013607	Enterobacter cloacae	SI	Clinical_samples
One Day In Denmark	Human	2018	PRJEB37711	SAMEA6656548	ERR4013608	Enterobacter cloacae	SI	Clinical_samples
One Day In Denmark	Human	2018	PRJEB37711	SAMEA6656549	ERR4013609	Citrobacter freundii	SI	Clinical_samples
One Day In Denmark	Human	2018	PRJEB37711	SAMEA6656550	ERR4013610	Citrobacter freundii	SI	Clinical_samples
One Day In Denmark	Human	2018	PRJEB37711	SAMEA6656551	ERR4013611	Enterobacter cloacae	SI	Clinical_samples

One Day In Denmark	Human	2018	PRJEB37711	SAMEA6656552	ERR4013612	Enterobacter bugandensis	SI	Clinical_samples
One Day In Denmark	Human	2018	PRJEB37711	SAMEA6656553	ERR4013613	Enterobacter cloacae	SI	Clinical_samples
One Day In Denmark	Human	2018	PRJEB37711	SAMEA6656554	ERR4013614	Salmonella enterica	SI	Clinical_samples
One Day In Denmark	Human	2018	PRJEB37711	SAMEA6656555	ERR4013615	Erwinia persicina	SI	Clinical_samples
One Day In Denmark	Human	2018	PRJEB37711	SAMEA6656556	ERR4013616	Pseudomonas aeruginosa	SI	Clinical_samples
One Day In Denmark	Human	2018	PRJEB37711	SAMEA6656557	ERR4013617	Pseudomonas aeruginosa	SI	Clinical_samples
One Day In Denmark	Human	2018	PRJEB37711	SAMEA6656558	ERR4013618	Pseudomonas aeruginosa	SI	Clinical_samples
One Day In Denmark	Human	2018	PRJEB37711	SAMEA6656559	ERR4013619	Pseudomonas aeruginosa	SI	Clinical_samples
One Day In Denmark	Human	2018	PRJEB37711	SAMEA6656560	ERR4013620	Pseudomonas aeruginosa	SI	Clinical_samples
One Day In Denmark	Human	2018	PRJEB37711	SAMEA6656561	ERR4013621	Pseudomonas aeruginosa	SI	Clinical_samples
One Day In Denmark	Human	2018	PRJEB37711	SAMEA6656562	ERR4013622	Pseudomonas aeruginosa	SI	Clinical_samples
One Day In Denmark	Human	2018	PRJEB37711	SAMEA6656563	ERR4013623	Pseudomonas aeruginosa	SI	Clinical_samples
One Day In Denmark	Human	2018	PRJEB37711	SAMEA6656564	ERR4013624	Pseudomonas aeruginosa	SI	Clinical_samples
One Day In Denmark	Human	2018	PRJEB37711	SAMEA6656565	ERR4013625	Pseudomonas aeruginosa	SI	Clinical_samples
One Day In Denmark	Human	2018	PRJEB37711	SAMEA6656566	ERR4013626	Pseudomonas aeruginosa	SI	Clinical_samples
One Day In Denmark	Human	2018	PRJEB37711	SAMEA6656567	ERR4013627	Pseudomonas aeruginosa	SI	Clinical_samples
One Day In Denmark	Human	2018	PRJEB37711	SAMEA6656568	ERR4013628	Pseudomonas aeruginosa	SI	Clinical_samples
One Day In Denmark	Human	2018	PRJEB37711	SAMEA6656569	ERR4013629	Pseudomonas aeruginosa	SI	Clinical_samples
One Day In Denmark	Human	2018	PRJEB37711	SAMEA6656570	ERR4013630	Pseudomonas aeruginosa	SI	Clinical_samples
One Day In Denmark	Human	2018	PRJEB37711	SAMEA6656571	ERR4013631	Pseudomonas aeruginosa	SI	Clinical_samples
One Day In Denmark	Human	2018	PRJEB37711	SAMEA6656572	ERR4013632	Pseudomonas aeruginosa	SI	Clinical_samples
One Day In Denmark	Human	2018	PRJEB37711	SAMEA6656573	ERR4013633	Pseudomonas aeruginosa	SI	Clinical_samples
One Day In Denmark	Human	2018	PRJEB37711	SAMEA6656574	ERR4013634	Pseudomonas aeruginosa	SI	Clinical_samples
One Day In Denmark	Human	2018	PRJEB37711	SAMEA6656575	ERR4013635	Pseudomonas aeruginosa	SI	Clinical_samples
One Day In Denmark	Human	2018	PRJEB37711	SAMEA6656576	ERR4013636	Providencia rettgeri	SI	Clinical_samples
One Day In Denmark	Human	2018	PRJEB37711	SAMEA6656577	ERR4013637	Stenotrophomonas maltophilia	SI	Clinical_samples
One Day In Denmark	Human	2018	PRJEB37711	SAMEA6656578	ERR4013638	Stenotrophomonas maltophilia	SI	Clinical_samples
One Day In Denmark	Human	2018	PRJEB37711	SAMEA6656579	ERR4013639	Serratia marcescens	SI	Clinical_samples
One Day In Denmark	Human	2018	PRJEB37711	SAMEA6656580	ERR4013640	Serratia marcescens	SI	Clinical_samples
One Day In Denmark	Human	2018	PRJEB37711	SAMEA6656581	ERR4013641	Yersinia enterocolitica	SI	Clinical_samples
One Day In Denmark	Human	2018	PRJEB37711	SAMEA6656582	ERR4013642	Staphylococcus aureus	SI	Clinical_samples
One Day In Denmark	Human	2018	PRJEB37711	SAMEA6656583	ERR4013643	Escherichia coli	SI	Clinical_samples
One Day In Denmark	Human	2018	PRJEB37711	SAMEA6656584	ERR4013644	Staphylococcus aureus	SI	Clinical_samples
One Day In Denmark	Human	2018	PRJEB37711	SAMEA6656585	ERR4013645	Staphylococcus aureus	SI	Clinical_samples

One Day In Denmark	Human	2018	PRJEB37711	SAMEA6656586	ERR4013646	Staphylococcus aureus	SI	Clinical_samples
One Day In Denmark	Human	2018	PRJEB37711	SAMEA6656587	ERR4013647	Staphylococcus aureus	SI	Clinical_samples
One Day In Denmark	Human	2018	PRJEB37711	SAMEA6656588	ERR4013648	Staphylococcus aureus	SI	Clinical_samples
One Day In Denmark	Human	2018	PRJEB37711	SAMEA6656589	ERR4013649	Staphylococcus aureus	SI	Clinical_samples
One Day In Denmark	Human	2018	PRJEB37711	SAMEA6656590	ERR4013650	Staphylococcus aureus	SI	Clinical_samples
One Day In Denmark	Human	2018	PRJEB37711	SAMEA6656591	ERR4013651	Staphylococcus aureus	SI	Clinical_samples
One Day In Denmark	Human	2018	PRJEB37711	SAMEA6656592	ERR4013652	Staphylococcus aureus	SI	Clinical_samples
One Day In Denmark	Human	2018	PRJEB37711	SAMEA6656593	ERR4013653	Staphylococcus aureus	SI	Clinical_samples
One Day In Denmark	Human	2018	PRJEB37711	SAMEA6656594	ERR4013654	Staphylococcus aureus	SI	Clinical_samples
One Day In Denmark	Human	2018	PRJEB37711	SAMEA6656595	ERR4013655	Staphylococcus aureus	SI	Clinical_samples
One Day In Denmark	Human	2018	PRJEB37711	SAMEA6656596	ERR4013656	Staphylococcus aureus	SI	Clinical_samples
One Day In Denmark	Human	2018	PRJEB37711	SAMEA6656597	ERR4013657	Staphylococcus aureus	SI	Clinical_samples
One Day In Denmark	Human	2018	PRJEB37711	SAMEA6656598	ERR4013658	Staphylococcus aureus	SI	Clinical_samples
One Day In Denmark	Human	2018	PRJEB37711	SAMEA6656599	ERR4013659	Staphylococcus aureus	SI	Clinical_samples
One Day In Denmark	Human	2018	PRJEB37711	SAMEA6656600	ERR4013660	Staphylococcus aureus	SI	Clinical_samples
One Day In Denmark	Human	2018	PRJEB37711	SAMEA6656601	ERR4013661	Staphylococcus aureus	SI	Clinical_samples
One Day In Denmark	Human	2018	PRJEB37711	SAMEA6656602	ERR4013662	Staphylococcus aureus	SI	Clinical_samples
One Day In Denmark	Human	2018	PRJEB37711	SAMEA6656603	ERR4013663	Staphylococcus aureus	SI	Clinical_samples
One Day In Denmark	Human	2018	PRJEB37711	SAMEA6656604	ERR4013664	Staphylococcus aureus	SI	Clinical_samples
One Day In Denmark	Human	2018	PRJEB37711	SAMEA6656605	ERR4013665	Staphylococcus aureus	SI	Clinical_samples
One Day In Denmark	Human	2018	PRJEB37711	SAMEA6656606	ERR4013666	Staphylococcus aureus	SI	Clinical_samples
One Day In Denmark	Human	2018	PRJEB37711	SAMEA6656607	ERR4013667	Staphylococcus aureus	SI	Clinical_samples
One Day In Denmark	Human	2018	PRJEB37711	SAMEA6656608	ERR4013668	Staphylococcus aureus	SI	Clinical_samples
One Day In Denmark	Human	2018	PRJEB37711	SAMEA6656609	ERR4013669	Staphylococcus aureus	SI	Clinical_samples
One Day In Denmark	Human	2018	PRJEB37711	SAMEA6656610	ERR4013670	Staphylococcus aureus	SI	Clinical_samples
One Day In Denmark	Human	2018	PRJEB37711	SAMEA6656611	ERR4013671	Staphylococcus aureus	SI	Clinical_samples
One Day In Denmark	Human	2018	PRJEB37711	SAMEA6656612	ERR4013672	Staphylococcus aureus	SI	Clinical_samples
One Day In Denmark	Human	2018	PRJEB37711	SAMEA6656613	ERR4013673	Staphylococcus aureus	SI	Clinical_samples
One Day In Denmark	Human	2018	PRJEB37711	SAMEA6656614	ERR4013674	Staphylococcus aureus	SI	Clinical_samples
One Day In Denmark	Human	2018	PRJEB37711	SAMEA6656615	ERR4013675	Staphylococcus aureus	SI	Clinical_samples
One Day In Denmark	Human	2018	PRJEB37711	SAMEA6656616	ERR4013676	Staphylococcus aureus	SI	Clinical_samples
One Day In Denmark	Human	2018	PRJEB37711	SAMEA6656617	ERR4013677	Staphylococcus aureus	SI	Clinical_samples
One Day In Denmark	Human	2018	PRJEB37711	SAMEA6656618	ERR4013678	Staphylococcus aureus	SI	Clinical_samples
One Day In Denmark	Human	2018	PRJEB37711	SAMEA6656619	ERR4013679	Staphylococcus aureus	SI	Clinical_samples

One Day In Denmark	Human	2018	PRJEB37711	SAMEA6656620	ERR4013680	Staphylococcus argenteus	SI	Clinical_samples
One Day In Denmark	Human	2018	PRJEB37711	SAMEA6656621	ERR4013681	Staphylococcus aureus	SI	Clinical_samples
One Day In Denmark	Human	2018	PRJEB37711	SAMEA6656622	ERR4013682	Staphylococcus aureus	SI	Clinical_samples
One Day In Denmark	Human	2018	PRJEB37711	SAMEA6656623	ERR4013683	Staphylococcus aureus	SI	Clinical_samples
One Day In Denmark	Human	2018	PRJEB37711	SAMEA6656624	ERR4013684	Staphylococcus aureus	SI	Clinical_samples
One Day In Denmark	Human	2018	PRJEB37711	SAMEA6656625	ERR4013685	Staphylococcus aureus	SI	Clinical_samples
One Day In Denmark	Human	2018	PRJEB37711	SAMEA6656626	ERR4013686	Staphylococcus aureus	SI	Clinical_samples
One Day In Denmark	Human	2018	PRJEB37711	SAMEA6656627	ERR4013687	Staphylococcus aureus	SI	Clinical_samples
One Day In Denmark	Human	2018	PRJEB37711	SAMEA6656628	ERR4013688	Staphylococcus aureus	SI	Clinical_samples
One Day In Denmark	Human	2018	PRJEB37711	SAMEA6656629	ERR4013689	Staphylococcus aureus	SI	Clinical_samples
One Day In Denmark	Human	2018	PRJEB37711	SAMEA6656630	ERR4013690	Staphylococcus aureus	SI	Clinical_samples
One Day In Denmark	Human	2018	PRJEB37711	SAMEA6656631	ERR4013691	Staphylococcus aureus	SI	Clinical_samples
One Day In Denmark	Human	2018	PRJEB37711	SAMEA6656632	ERR4013692	Streptococcus dysgalactiae	SI	Clinical_samples
One Day In Denmark	Human	2018	PRJEB37711	SAMEA6656633	ERR4013693	Staphylococcus aureus	SI	Clinical_samples
One Day In Denmark	Human	2018	PRJEB37711	SAMEA6656634	ERR4013694	Staphylococcus aureus	SI	Clinical_samples
One Day In Denmark	Human	2018	PRJEB37711	SAMEA6656635	ERR4013695	Staphylococcus aureus	SI	Clinical_samples
One Day In Denmark	Human	2018	PRJEB37711	SAMEA6656636	ERR4013696	Staphylococcus aureus	SI	Clinical_samples
One Day In Denmark	Human	2018	PRJEB37711	SAMEA6656637	ERR4013697	Staphylococcus aureus	SI	Clinical_samples
One Day In Denmark	Human	2018	PRJEB37711	SAMEA6656638	ERR4013698	Staphylococcus aureus	SI	Clinical_samples
One Day In Denmark	Human	2018	PRJEB37711	SAMEA6656639	ERR4013699	Staphylococcus aureus	SI	Clinical_samples
One Day In Denmark	Human	2018	PRJEB37711	SAMEA6656640	ERR4013700	Staphylococcus aureus	SI	Clinical_samples
One Day In Denmark	Human	2018	PRJEB37711	SAMEA6656641	ERR4013701	Staphylococcus aureus	SI	Clinical_samples
One Day In Denmark	Human	2018	PRJEB37711	SAMEA6656642	ERR4013702	Staphylococcus aureus	SI	Clinical_samples
One Day In Denmark	Human	2018	PRJEB37711	SAMEA6656643	ERR4013703	Staphylococcus aureus	SI	Clinical_samples
One Day In Denmark	Human	2018	PRJEB37711	SAMEA6656644	ERR4013704	Staphylococcus aureus	SI	Clinical_samples
One Day In Denmark	Human	2018	PRJEB37711	SAMEA6656645	ERR4013705	Staphylococcus aureus	SI	Clinical_samples
One Day In Denmark	Human	2018	PRJEB37711	SAMEA6656646	ERR4013706	Staphylococcus aureus	SI	Clinical_samples
One Day In Denmark	Human	2018	PRJEB37711	SAMEA6656647	ERR4013707	Staphylococcus aureus	SI	Clinical_samples
One Day In Denmark	Human	2018	PRJEB37711	SAMEA6656648	ERR4013708	Staphylococcus aureus	SI	Clinical_samples
One Day In Denmark	Human	2018	PRJEB37711	SAMEA6656649	ERR4013709	Staphylococcus aureus	SI	Clinical_samples
One Day In Denmark	Human	2018	PRJEB37711	SAMEA6656650	ERR4013710	Staphylococcus aureus	SI	Clinical_samples
One Day In Denmark	Human	2018	PRJEB37711	SAMEA6656651	ERR4013711	Staphylococcus aureus	SI	Clinical_samples
One Day In Denmark	Human	2018	PRJEB37711	SAMEA6656652	ERR4013712	Staphylococcus aureus	SI	Clinical_samples
One Day In Denmark	Human	2018	PRJEB37711	SAMEA6656653	ERR4013713	Staphylococcus aureus	SI	Clinical_samples

One Day In Denmark	Human	2018	PRJEB37711	SAMEA6656654	ERR4013714	Staphylococcus aureus	SI	Clinical_samples
One Day In Denmark	Human	2018	PRJEB37711	SAMEA6656655	ERR4013715	Staphylococcus aureus	SI	Clinical_samples
One Day In Denmark	Human	2018	PRJEB37711	SAMEA6656656	ERR4013716	Staphylococcus aureus	SI	Clinical_samples
One Day In Denmark	Human	2018	PRJEB37711	SAMEA6656657	ERR4013717	Staphylococcus aureus	SI	Clinical_samples
One Day In Denmark	Human	2018	PRJEB37711	SAMEA6656658	ERR4013718	Staphylococcus aureus	SI	Clinical_samples
One Day In Denmark	Human	2018	PRJEB37711	SAMEA6656659	ERR4013719	Staphylococcus aureus	SI	Clinical_samples
One Day In Denmark	Human	2018	PRJEB37711	SAMEA6656660	ERR4013720	Staphylococcus aureus	SI	Clinical_samples
One Day In Denmark	Human	2018	PRJEB37711	SAMEA6656661	ERR4013721	Staphylococcus aureus	SI	Clinical_samples
One Day In Denmark	Human	2018	PRJEB37711	SAMEA6656662	ERR4013722	Staphylococcus aureus	SI	Clinical_samples
One Day In Denmark	Human	2018	PRJEB37711	SAMEA6656663	ERR4013723	Staphylococcus aureus	SI	Clinical_samples
One Day In Denmark	Human	2018	PRJEB37711	SAMEA6656664	ERR4013724	Staphylococcus aureus	SI	Clinical_samples
One Day In Denmark	Human	2018	PRJEB37711	SAMEA6656665	ERR4013725	Staphylococcus aureus	SI	Clinical_samples
One Day In Denmark	Human	2018	PRJEB37711	SAMEA6656666	ERR4013726	Staphylococcus aureus	SI	Clinical_samples
One Day In Denmark	Human	2018	PRJEB37711	SAMEA6656667	ERR4013727	Staphylococcus aureus	SI	Clinical_samples
One Day In Denmark	Human	2018	PRJEB37711	SAMEA6656669	ERR4013728	Staphylococcus aureus	SI	Clinical_samples
One Day In Denmark	Human	2018	PRJEB37711	SAMEA6656670	ERR4013729	Staphylococcus aureus	SI	Clinical_samples
One Day In Denmark	Human	2018	PRJEB37711	SAMEA6656671	ERR4013730	Staphylococcus aureus	SI	Clinical_samples
One Day In Denmark	Human	2018	PRJEB37711	SAMEA6656672	ERR4013731	Staphylococcus epidermidis	SI	Clinical_samples
One Day In Denmark	Human	2018	PRJEB37711	SAMEA6656673	ERR4013732	Staphylococcus epidermidis	SI	Clinical_samples
One Day In Denmark	Human	2018	PRJEB37711	SAMEA6656674	ERR4013733	Enterococcus faecium	SI	Clinical_samples
One Day In Denmark	Human	2018	PRJEB37711	SAMEA6656675	ERR4013734	Staphylococcus epidermidis	SI	Clinical_samples
One Day In Denmark	Human	2018	PRJEB37711	SAMEA6656676	ERR4013735	Staphylococcus epidermidis	SI	Clinical_samples
One Day In Denmark	Human	2018	PRJEB37711	SAMEA6656677	ERR4013736	Staphylococcus epidermidis	SI	Clinical_samples
One Day In Denmark	Human	2018	PRJEB37711	SAMEA6656678	ERR4013737	Staphylococcus epidermidis	SI	Clinical_samples
One Day In Denmark	Human	2018	PRJEB37711	SAMEA6656679	ERR4013738	Staphylococcus epidermidis	SI	Clinical_samples
One Day In Denmark	Human	2018	PRJEB37711	SAMEA6656680	ERR4013739	Staphylococcus epidermidis	SI	Clinical_samples
One Day In Denmark	Human	2018	PRJEB37711	SAMEA6656681	ERR4013740	Staphylococcus hominis	SI	Clinical_samples
One Day In Denmark	Human	2018	PRJEB37711	SAMEA6656682	ERR4013741	Staphylococcus saprophyticus	SI	Clinical_samples
One Day In Denmark	Human	2018	PRJEB37711	SAMEA6656683	ERR4013742	Staphylococcus lugdunensis	SI	Clinical_samples
One Day In Denmark	Human	2018	PRJEB37711	SAMEA6656684	ERR4013743	Staphylococcus lugdunensis	SI	Clinical_samples
One Day In Denmark	Human	2018	PRJEB37711	SAMEA6656685	ERR4013744	Staphylococcus lugdunensis	SI	Clinical_samples
One Day In Denmark	Human	2018	PRJEB37711	SAMEA6656686	ERR4013745	Corynebacterium striatum	SI	Clinical_samples
One Day In Denmark	Human	2018	PRJEB37711	SAMEA6656687	ERR4013746	Streptococcus agalactiae	SI	Clinical_samples
One Day In Denmark	Human	2018	PRJEB37711	SAMEA6656688	ERR4013747	Streptococcus agalactiae	SI	Clinical_samples

One Day In Denmark	Human	2018	PRJEB37711	SAMEA6656689	ERR4013748	Streptococcus agalactiae	SI	Clinical_samples
One Day In Denmark	Human	2018	PRJEB37711	SAMEA6656690	ERR4013749	Staphylococcus aureus	SI	Clinical_samples
One Day In Denmark	Human	2018	PRJEB37711	SAMEA6656691	ERR4013750	Streptococcus agalactiae	SI	Clinical_samples
One Day In Denmark	Human	2018	PRJEB37711	SAMEA6656692	ERR4013751	Streptococcus agalactiae	SI	Clinical_samples
One Day In Denmark	Human	2018	PRJEB37711	SAMEA6656693	ERR4013752	Streptococcus agalactiae	SI	Clinical_samples
One Day In Denmark	Human	2018	PRJEB37711	SAMEA6656694	ERR4013753	Streptococcus agalactiae	SI	Clinical_samples
One Day In Denmark	Human	2018	PRJEB37711	SAMEA6656695	ERR4013754	Streptococcus agalactiae	SI	Clinical_samples
One Day In Denmark	Human	2018	PRJEB37711	SAMEA6656696	ERR4013755	Streptococcus agalactiae	SI	Clinical_samples
One Day In Denmark	Human	2018	PRJEB37711	SAMEA6656697	ERR4013756	Streptococcus agalactiae	SI	Clinical_samples
One Day In Denmark	Human	2018	PRJEB37711	SAMEA6656698	ERR4013757	Streptococcus agalactiae	SI	Clinical_samples
One Day In Denmark	Human	2018	PRJEB37711	SAMEA6656699	ERR4013758	Streptococcus agalactiae	SI	Clinical_samples
One Day In Denmark	Human	2018	PRJEB37711	SAMEA6656700	ERR4013759	Streptococcus agalactiae	SI	Clinical_samples
One Day In Denmark	Human	2018	PRJEB37711	SAMEA6656701	ERR4013760	Streptococcus agalactiae	SI	Clinical_samples
One Day In Denmark	Human	2018	PRJEB37711	SAMEA6656702	ERR4013761	Streptococcus agalactiae	SI	Clinical_samples
One Day In Denmark	Human	2018	PRJEB37711	SAMEA6656703	ERR4013762	Streptococcus agalactiae	SI	Clinical_samples
One Day In Denmark	Human	2018	PRJEB37711	SAMEA6656704	ERR4013763	Streptococcus agalactiae	SI	Clinical_samples
One Day In Denmark	Human	2018	PRJEB37711	SAMEA6656705	ERR4013764	Streptococcus agalactiae	SI	Clinical_samples
One Day In Denmark	Human	2018	PRJEB37711	SAMEA6656706	ERR4013765	Streptococcus agalactiae	SI	Clinical_samples
One Day In Denmark	Human	2018	PRJEB37711	SAMEA6656707	ERR4013766	Streptococcus agalactiae	SI	Clinical_samples
One Day In Denmark	Human	2018	PRJEB37711	SAMEA6656708	ERR4013767	Streptococcus agalactiae	SI	Clinical_samples
One Day In Denmark	Human	2018	PRJEB37711	SAMEA6656709	ERR4013768	Streptococcus agalactiae	SI	Clinical_samples
One Day In Denmark	Human	2018	PRJEB37711	SAMEA6656710	ERR4013769	Streptococcus agalactiae	SI	Clinical_samples
One Day In Denmark	Human	2018	PRJEB37711	SAMEA6656711	ERR4013770	Streptococcus dysgalactiae	SI	Clinical_samples
One Day In Denmark	Human	2018	PRJEB37711	SAMEA6656712	ERR4013771	Streptococcus dysgalactiae	SI	Clinical_samples
One Day In Denmark	Human	2018	PRJEB37711	SAMEA6656713	ERR4013772	Streptococcus dysgalactiae	SI	Clinical_samples
One Day In Denmark	Human	2018	PRJEB37711	SAMEA6656714	ERR4013773	Streptococcus dysgalactiae	SI	Clinical_samples
One Day In Denmark	Human	2018	PRJEB37711	SAMEA6656715	ERR4013774	Enterococcus faecalis	SI	Clinical_samples
One Day In Denmark	Human	2018	PRJEB37711	SAMEA6656716	ERR4013775	Streptococcus dysgalactiae	SI	Clinical_samples
One Day In Denmark	Human	2018	PRJEB37711	SAMEA6656717	ERR4013776	Streptococcus dysgalactiae	SI	Clinical_samples
One Day In Denmark	Human	2018	PRJEB37711	SAMEA6656718	ERR4013777	Streptococcus dysgalactiae	SI	Clinical_samples
One Day In Denmark	Human	2018	PRJEB37711	SAMEA6656719	ERR4013778	Streptococcus dysgalactiae	SI	Clinical_samples
One Day In Denmark	Human	2018	PRJEB37711	SAMEA6656720	ERR4013779	Streptococcus dysgalactiae	SI	Clinical_samples
One Day In Denmark	Human	2018	PRJEB37711	SAMEA6656721	ERR4013780	Streptococcus dysgalactiae	SI	Clinical_samples
One Day In Denmark	Human	2018	PRJEB37711	SAMEA6656722	ERR4013781	Streptococcus dysgalactiae	SI	Clinical_samples

One Day In Denmark	Human	2018	PRJEB37711	SAMEA6656723	ERR4013782	Streptococcus oralis	SI	Clinical_samples
One Day In Denmark	Human	2018	PRJEB37711	SAMEA6656724	ERR4013783	Streptococcus pneumoniae	SI	Clinical_samples
One Day In Denmark	Human	2018	PRJEB37711	SAMEA6656725	ERR4013784	Streptococcus pneumoniae	SI	Clinical_samples
One Day In Denmark	Human	2018	PRJEB37711	SAMEA6656726	ERR4013785	Streptococcus pneumoniae	SI	Clinical_samples
One Day In Denmark	Human	2018	PRJEB37711	SAMEA6656727	ERR4013786	Streptococcus pneumoniae	SI	Clinical_samples
One Day In Denmark	Human	2018	PRJEB37711	SAMEA6656728	ERR4013787	Streptococcus pneumoniae	SI	Clinical_samples
One Day In Denmark	Human	2018	PRJEB37711	SAMEA6656729	ERR4013788	Streptococcus pneumoniae	SI	Clinical_samples
One Day In Denmark	Human	2018	PRJEB37711	SAMEA6656730	ERR4013789	Streptococcus pneumoniae	SI	Clinical_samples
One Day In Denmark	Human	2018	PRJEB37711	SAMEA6656731	ERR4013790	Streptococcus pyogenes	SI	Clinical_samples
One Day In Denmark	Human	2018	PRJEB37711	SAMEA6656732	ERR4013791	Streptococcus pyogenes	SI	Clinical_samples
One Day In Denmark	Human	2018	PRJEB37711	SAMEA6656733	ERR4013792	Streptococcus pyogenes	SI	Clinical_samples
One Day In Denmark	Human	2018	PRJEB37711	SAMEA6656734	ERR4013793	Streptococcus pyogenes	SI	Clinical_samples
One Day In Denmark	Human	2018	PRJEB37711	SAMEA6656735	ERR4013794	Corynebacterium striatum	SI	Clinical_samples
One Day In Denmark	Human	2018	PRJEB37711	SAMEA6656736	ERR4013795	Streptococcus pyogenes	SI	Clinical_samples
One Day In Denmark	Human	2018	PRJEB37711	SAMEA6656737	ERR4013796	Streptococcus pyogenes	SI	Clinical_samples
One Day In Denmark	Human	2018	PRJEB37711	SAMEA6656738	ERR4013797	Streptococcus pyogenes	SI	Clinical_samples
One Day In Denmark	Human	2018	PRJEB37711	SAMEA6656739	ERR4013798	Streptococcus pyogenes	SI	Clinical_samples
One Day In Denmark	Human	2018	PRJEB37711	SAMEA6656740	ERR4013799	Staphylococcus aureus	SI	Clinical_samples
One Day In Denmark	Human	2018	PRJEB37711	SAMEA6656741	ERR4013800	Staphylococcus aureus	SI	Clinical_samples
One Day In Denmark	Human	2018	PRJEB37711	SAMEA6656742	ERR4013801	Streptococcus pyogenes	SI	Clinical_samples
One Day In Denmark	Human	2018	PRJEB37711	SAMEA6656743	ERR4013802	Streptococcus gordonii	SI	Clinical_samples
One Day In Denmark	Human	2018	PRJEB37711	SAMEA6656744	ERR4013803	Streptococcus mitis	SI	Clinical_samples
One Day In Denmark	Human	2018	PRJEB37711	SAMEA6656745	ERR4013804	Enterococcus faecalis	SI	Clinical_samples
One Day In Denmark	Human	2018	PRJEB37711	SAMEA6656746	ERR4013805	Enterococcus faecalis	SI	Clinical_samples
One Day In Denmark	Human	2018	PRJEB37711	SAMEA6656747	ERR4013806	Enterococcus faecalis	SI	Clinical_samples
One Day In Denmark	Human	2018	PRJEB37711	SAMEA6656748	ERR4013807	Enterococcus faecalis	SI	Clinical_samples
One Day In Denmark	Human	2018	PRJEB37711	SAMEA6656749	ERR4013808	Enterococcus faecalis	SI	Clinical_samples
One Day In Denmark	Human	2018	PRJEB37711	SAMEA6656750	ERR4013809	Enterococcus faecalis	SI	Clinical_samples
One Day In Denmark	Human	2018	PRJEB37711	SAMEA6656751	ERR4013810	Enterococcus faecalis	SI	Clinical_samples
One Day In Denmark	Human	2018	PRJEB37711	SAMEA6656752	ERR4013811	Enterococcus faecalis	SI	Clinical_samples
One Day In Denmark	Human	2018	PRJEB37711	SAMEA6656753	ERR4013812	Enterococcus faecalis	SI	Clinical_samples
One Day In Denmark	Human	2018	PRJEB37711	SAMEA6656754	ERR4013813	Enterococcus faecalis	SI	Clinical_samples
One Day In Denmark	Human	2018	PRJEB37711	SAMEA6656755	ERR4013814	Enterococcus faecalis	SI	Clinical_samples
One Day In Denmark	Human	2018	PRJEB37711	SAMEA6656756	ERR4013815	Enterococcus faecalis	SI	Clinical_samples

One Day In Denmark	Human	2018	PRJEB37711	SAMEA6656757	ERR4013816	Enterococcus faecalis	SI	Clinical_samples
One Day In Denmark	Human	2018	PRJEB37711	SAMEA6656758	ERR4013817	Enterococcus faecalis	SI	Clinical_samples
One Day In Denmark	Human	2018	PRJEB37711	SAMEA6656759	ERR4013818	Enterococcus faecalis	SI	Clinical_samples
One Day In Denmark	Human	2018	PRJEB37711	SAMEA6656760	ERR4013819	Enterococcus faecium	SI	Clinical_samples
One Day In Denmark	Human	2018	PRJEB37711	SAMEA6656761	ERR4013820	Enterococcus faecium	SI	Clinical_samples
One Day In Denmark	Human	2018	PRJEB37711	SAMEA6656762	ERR4013821	Enterococcus faecium	SI	Clinical_samples
One Day In Denmark	Human	2018	PRJEB37711	SAMEA6656763	ERR4013822	Enterococcus faecium	SI	Clinical_samples
One Day In Denmark	Human	2018	PRJEB37711	SAMEA6656764	ERR4013823	Enterococcus faecium	SI	Clinical_samples
One Day In Denmark	Human	2018	PRJEB37711	SAMEA6656765	ERR4013824	Enterococcus faecium	SI	Clinical_samples
One Day In Denmark	Human	2018	PRJEB37711	SAMEA6656766	ERR4013825	Enterococcus faecalis	SI	Clinical_samples
One Day In Denmark	Human	2018	PRJEB37711	SAMEA6656767	ERR4013826	Enterococcus faecalis	SI	Clinical_samples
One Day In Denmark	Human	2018	PRJEB37711	SAMEA6656768	ERR4013827	Enterococcus faecalis	SI	Clinical_samples
One Day In Denmark	Human	2018	PRJEB37711	SAMEA6656769	ERR4013828	Enterococcus faecalis	SI	Clinical_samples
One Day In Denmark	Human	2018	PRJEB37711	SAMEA6656770	ERR4013829	Enterococcus faecium	SI	Clinical_samples
One Day In Denmark	Human	2018	PRJEB37711	SAMEA6656771	ERR4013830	Enterococcus faecalis	SI	Clinical_samples
One Day In Denmark	Human	2018	PRJEB37711	SAMEA6656772	ERR4013831	Enterococcus faecalis	SI	Clinical_samples
One Day In Denmark	Human	2018	PRJEB37711	SAMEA6656773	ERR4013832	Enterococcus faecium	SI	Clinical_samples
One Day In Denmark	Human	2018	PRJEB37711	SAMEA6656774	ERR4013833	Aerococcus sanguinicola	SI	Clinical_samples
One Day In Denmark	Human	2018	PRJEB37711	SAMEA6656775	ERR4013834	Escherichia coli	SI	Clinical_samples
One Day In Denmark	Human	2018	PRJEB37711	SAMEA6656776	ERR4013835	Aerococcus urinae	SI	Clinical_samples
One Day In Denmark	Human	2018	PRJEB37711	SAMEA6656777	ERR4013836	Aerococcus urinae	SI	Clinical_samples
One Day In Denmark	Human	2018	PRJEB37711	SAMEA6656778	ERR4013837	Moraxella catarrhalis	SI	Clinical_samples
One Day In Denmark	Human	2018	PRJEB37711	SAMEA6656779	ERR4013838	Moraxella catarrhalis	SI	Clinical_samples
One Day In Denmark	Human	2018	PRJEB37711	SAMEA6656780	ERR4013839	Moraxella catarrhalis	SI	Clinical_samples
One Day In Denmark	Human	2018	PRJEB37711	SAMEA6656781	ERR4013840	Moraxella catarrhalis	SI	Clinical_samples
One Day In Denmark	Human	2018	PRJEB37711	SAMEA6656782	ERR4013841	Moraxella catarrhalis	SI	Clinical_samples
One Day In Denmark	Human	2018	PRJEB37711	SAMEA6656783	ERR4013842	Moraxella catarrhalis	SI	Clinical_samples
One Day In Denmark	Human	2018	PRJEB37711	SAMEA6656784	ERR4013843	Moraxella catarrhalis	SI	Clinical_samples
One Day In Denmark	Human	2018	PRJEB37711	SAMEA6656785	ERR4013844	Moraxella catarrhalis	SI	Clinical_samples
One Day In Denmark	Human	2018	PRJEB37711	SAMEA6656786	ERR4013845	Moraxella catarrhalis	SI	Clinical_samples
One Day In Denmark	Human	2018	PRJEB37711	SAMEA6656787	ERR4013846	Haemophilus influenzae	SI	Clinical_samples
One Day In Denmark	Human	2018	PRJEB37711	SAMEA6656788	ERR4013847	Haemophilus influenzae	SI	Clinical_samples
One Day In Denmark	Human	2018	PRJEB37711	SAMEA6656789	ERR4013848	Haemophilus influenzae	SI	Clinical_samples
One Day In Denmark	Human	2018	PRJEB37711	SAMEA6656790	ERR4013849	Haemophilus influenzae	SI	Clinical_samples

One Day In Denmark	Human	2018	PRJEB37711	SAMEA6656791	ERR4013850	Haemophilus influenzae	SI	Clinical_samples
One Day In Denmark	Human	2018	PRJEB37711	SAMEA6656792	ERR4013851	Haemophilus influenzae	SI	Clinical_samples
One Day In Denmark	Human	2018	PRJEB37711	SAMEA6656793	ERR4013852	Haemophilus influenzae	SI	Clinical_samples
One Day In Denmark	Human	2018	PRJEB37711	SAMEA6656794	ERR4013853	Haemophilus influenzae	SI	Clinical_samples
One Day In Denmark	Human	2018	PRJEB37711	SAMEA6656795	ERR4013854	Haemophilus influenzae	SI	Clinical_samples
One Day In Denmark	Human	2018	PRJEB37711	SAMEA6656796	ERR4013855	Haemophilus influenzae	SI	Clinical_samples
One Day In Denmark	Human	2018	PRJEB37711	SAMEA6656797	ERR4013856	Haemophilus influenzae	SI	Clinical_samples
One Day In Denmark	Human	2018	PRJEB37711	SAMEA6656798	ERR4013857	Haemophilus influenzae	SI	Clinical_samples
One Day In Denmark	Human	2018	PRJEB37711	SAMEA6656799	ERR4013858	Haemophilus influenzae	SI	Clinical_samples
One Day In Denmark	Human	2018	PRJEB37711	SAMEA6656800	ERR4013859	Haemophilus influenzae	SI	Clinical_samples
One Day In Denmark	Human	2018	PRJEB37711	SAMEA6656801	ERR4013860	Moraxella catarrhalis	SI	Clinical_samples
One Day In Denmark	Human	2018	PRJEB37711	SAMEA6656802	ERR4013861	Peptoniphilus harei	SI	Clinical_samples
One Day In Denmark	Human	2018	PRJEB37711	SAMEA6656803	ERR4013862	Pseudomonas aeruginosa	SI	Clinical_samples
One Day In Denmark	Human	2018	PRJEB37711	SAMEA6656804	ERR4013863	Anaerococcus hydrogenalis	SI	Clinical_samples
One Day In Denmark	Human	2018	PRJEB37711	SAMEA6656805	ERR4013864	Cutibacterium acnes	SI	Clinical_samples
One Day In Denmark	Human	2018	PRJEB37711	SAMEA6656806	ERR4013865	Bacteroides fragilis	SI	Clinical_samples
One Day In Denmark	Human	2018	PRJEB37711	SAMEA6656807	ERR4013866	Bacteroides fragilis	SI	Clinical_samples
One Day In Denmark	Human	2018	PRJEB37711	SAMEA6656808	ERR4013867	Bacteroides fragilis	SI	Clinical_samples
One Day In Denmark	Human	2018	PRJEB37711	SAMEA6656809	ERR4013868	Actinomyces oris	SI	Clinical_samples
One Day In Denmark	Human	2018	PRJEB37711	SAMEA6656810	ERR4013869	Actinomyces oris	SI	Clinical_samples
One Day In Denmark	Human	2018	PRJEB37711	SAMEA6656811	ERR4013870	Escherichia coli	SI	Clinical_samples
One Day In Denmark	Human	2018	PRJEB37711	SAMEA6656812	ERR4013871	Escherichia coli	SI	Clinical_samples
One Day In Denmark	Human	2018	PRJEB37711	SAMEA6656813	ERR4013872	Escherichia coli	SI	Clinical_samples
One Day In Denmark	Human	2018	PRJEB37711	SAMEA6656814	ERR4013873	Escherichia coli	SI	Clinical_samples
One Day In Denmark	Human	2018	PRJEB37711	SAMEA6656815	ERR4013874	Escherichia coli	SI	Clinical_samples
One Day In Denmark	Human	2018	PRJEB37711	SAMEA6656816	ERR4013875	Escherichia coli	SI	Clinical_samples
One Day In Denmark	Human	2018	PRJEB37711	SAMEA6656817	ERR4013876	Escherichia coli	SI	Clinical_samples
One Day In Denmark	Human	2018	PRJEB37711	SAMEA6656818	ERR4013877	Escherichia coli	SI	Clinical_samples
One Day In Denmark	Human	2018	PRJEB37711	SAMEA6656819	ERR4013878	Escherichia coli	SI	Clinical_samples
One Day In Denmark	Human	2018	PRJEB37711	SAMEA6656820	ERR4013879	Escherichia coli	SI	Clinical_samples
One Day In Denmark	Human	2018	PRJEB37711	SAMEA6656821	ERR4013880	Proteus mirabilis	SI	Clinical_samples
One Day In Denmark	Human	2018	PRJEB37711	SAMEA6656822	ERR4013881	Escherichia coli	SI	Clinical_samples
One Day In Denmark	Human	2018	PRJEB37711	SAMEA6656823	ERR4013882	Citrobacter koseri	SI	Clinical_samples
One Day In Denmark	Human	2018	PRJEB37711	SAMEA6656824	ERR4013883	Escherichia coli	SI	Clinical_samples

One Day In Denmark	Human	2018	PRJEB37711	SAMEA6656825	ERR4013884	Escherichia coli	SI	Clinical_samples
One Day In Denmark	Human	2018	PRJEB37711	SAMEA6656826	ERR4013885	Escherichia coli	SI	Clinical_samples
One Day In Denmark	Human	2018	PRJEB37711	SAMEA6656827	ERR4013886	Enterobacter cloacae	SI	Clinical_samples
One Day In Denmark	Human	2018	PRJEB37711	SAMEA6656828	ERR4013887	Escherichia coli	SI	Clinical_samples
One Day In Denmark	Human	2018	PRJEB37711	SAMEA6656829	ERR4013888	Escherichia coli	SI	Clinical_samples
One Day In Denmark	Human	2018	PRJEB37711	SAMEA6656830	ERR4013889	Escherichia coli	SI	Clinical_samples
One Day In Denmark	Human	2018	PRJEB37711	SAMEA6656831	ERR4013890	Escherichia coli	SI	Clinical_samples
One Day In Denmark	Human	2018	PRJEB37711	SAMEA6656832	ERR4013891	Escherichia coli	SI	Clinical_samples
One Day In Denmark	Human	2018	PRJEB37711	SAMEA6656833	ERR4013892	Escherichia coli	SI	Clinical_samples
One Day In Denmark	Human	2018	PRJEB37711	SAMEA6656834	ERR4013893	Escherichia coli	SI	Clinical_samples
One Day In Denmark	Human	2018	PRJEB37711	SAMEA6656835	ERR4013894	Escherichia coli	SI	Clinical_samples
One Day In Denmark	Human	2018	PRJEB37711	SAMEA6656836	ERR4013895	Escherichia coli	SI	Clinical_samples
One Day In Denmark	Human	2018	PRJEB37711	SAMEA6656837	ERR4013896	Escherichia coli	SI	Clinical_samples
One Day In Denmark	Human	2018	PRJEB37711	SAMEA6656838	ERR4013897	Escherichia coli	SI	Clinical_samples
One Day In Denmark	Human	2018	PRJEB37711	SAMEA6656839	ERR4013898	Escherichia coli	SI	Clinical_samples
One Day In Denmark	Human	2018	PRJEB37711	SAMEA6656840	ERR4013899	Escherichia coli	SI	Clinical_samples
One Day In Denmark	Human	2018	PRJEB37711	SAMEA6656841	ERR4013900	Morganella morganii	SI	Clinical_samples
One Day In Denmark	Human	2018	PRJEB37711	SAMEA6656842	ERR4013901	Pseudomonas aeruginosa	SI	Clinical_samples
One Day In Denmark	Human	2018	PRJEB37711	SAMEA6656843	ERR4013902	Klebsiella aerogenes	SI	Clinical_samples
One Day In Denmark	Human	2018	PRJEB37711	SAMEA6656844	ERR4013903	Moraxella catarrhalis	SI	Clinical_samples
One Day In Denmark	Human	2018	PRJEB37711	SAMEA6656845	ERR4013904	Haemophilus influenzae	SI	Clinical_samples
One Day In Denmark	Human	2018	PRJEB37711	SAMEA6656846	ERR4013905	Pseudomonas aeruginosa	SI	Clinical_samples
One Day In Denmark	Human	2018	PRJEB37711	SAMEA6656847	ERR4013906	Pseudomonas aeruginosa	SI	Clinical_samples
One Day In Denmark	Human	2018	PRJEB37711	SAMEA6656848	ERR4013907	Klebsiella pneumoniae	SI	Clinical_samples
One Day In Denmark	Human	2018	PRJEB37711	SAMEA6656849	ERR4013908	Klebsiella pneumoniae	SI	Clinical_samples
One Day In Denmark	Human	2018	PRJEB37711	SAMEA6656850	ERR4013909	Staphylococcus epidermidis	SI	Clinical_samples
One Day In Denmark	Human	2018	PRJEB37711	SAMEA6656851	ERR4013910	Staphylococcus hominis	SI	Clinical_samples
One Day In Denmark	Human	2018	PRJEB37711	SAMEA6656852	ERR4013911	Staphylococcus epidermidis	SI	Clinical_samples
One Day In Denmark	Human	2018	PRJEB37711	SAMEA6656853	ERR4013912	Staphylococcus epidermidis	SI	Clinical_samples
One Day In Denmark	Human	2018	PRJEB37711	SAMEA6656854	ERR4013913	Klebsiella pneumoniae	SI	Clinical_samples
One Day In Denmark	Human	2018	PRJEB37711	SAMEA6656855	ERR4013914	Klebsiella pneumoniae	SI	Clinical_samples
One Day In Denmark	Human	2018	PRJEB37711	SAMEA6656856	ERR4013915	Moraxella catarrhalis	SI	Clinical_samples
One Day In Denmark	Human	2018	PRJEB37711	SAMEA6656857	ERR4013916	Haemophilus influenzae	SI	Clinical_samples
One Day In Denmark	Human	2018	PRJEB37711	SAMEA6656858	ERR4013917	Haemophilus influenzae	SI	Clinical_samples

One Day In Denmark	Human	2018	PRJEB37711	SAMEA6656859	ERR4013918	Klebsiella pneumoniae	SI	Clinical_samples
One Day In Denmark	Human	2018	PRJEB37711	SAMEA6656860	ERR4013919	Escherichia coli	SI	Clinical_samples
One Day In Denmark	Human	2018	PRJEB37711	SAMEA6656861	ERR4013920	Escherichia coli	SI	Clinical_samples
One Day In Denmark	Human	2018	PRJEB37711	SAMEA6656862	ERR4013921	Escherichia coli	SI	Clinical_samples
One Day In Denmark	Human	2018	PRJEB37711	SAMEA6656863	ERR4013922	Escherichia coli	SI	Clinical_samples
One Day In Denmark	Human	2018	PRJEB37711	SAMEA6656864	ERR4013923	Klebsiella oxytoca	SI	Clinical_samples
One Day In Denmark	Human	2018	PRJEB37711	SAMEA6656865	ERR4013924	Pseudomonas aeruginosa	SI	Clinical_samples
One Day In Denmark	Human	2018	PRJEB37711	SAMEA6656866	ERR4013925	Escherichia coli	SI	Clinical_samples
One Day In Denmark	Human	2018	PRJEB37711	SAMEA6656867	ERR4013926	Escherichia coli	SI	Clinical_samples
One Day In Denmark	Human	2018	PRJEB37711	SAMEA6656868	ERR4013927	Escherichia coli	SI	Clinical_samples
One Day In Denmark	Human	2018	PRJEB37711	SAMEA6656869	ERR4013928	Escherichia coli	SI	Clinical_samples
One Day In Denmark	Human	2018	PRJEB37711	SAMEA6656870	ERR4013929	Escherichia coli	SI	Clinical_samples
One Day In Denmark	Human	2018	PRJEB37711	SAMEA6656871	ERR4013930	Escherichia coli	SI	Clinical_samples
One Day In Denmark	Human	2018	PRJEB37711	SAMEA6656872	ERR4013931	Escherichia coli	SI	Clinical_samples
One Day In Denmark	Human	2018	PRJEB37711	SAMEA6656873	ERR4013932	Escherichia coli	SI	Clinical_samples
One Day In Denmark	Human	2018	PRJEB37711	SAMEA6656874	ERR4013933	Proteus mirabilis	SI	Clinical_samples
One Day In Denmark	Human	2018	PRJEB37711	SAMEA6656875	ERR4013934	Escherichia coli	SI	Clinical_samples
One Day In Denmark	Human	2018	PRJEB37711	SAMEA6656876	ERR4013935	Escherichia coli	SI	Clinical_samples
One Day In Denmark	Human	2018	PRJEB37711	SAMEA6656877	ERR4013936	Escherichia coli	SI	Clinical_samples
One Day In Denmark	Human	2018	PRJEB37711	SAMEA6656878	ERR4013937	Escherichia coli	SI	Clinical_samples
One Day In Denmark	Human	2018	PRJEB37711	SAMEA6656879	ERR4013938	Escherichia coli	SI	Clinical_samples
One Day In Denmark	Human	2018	PRJEB37711	SAMEA6656880	ERR4013939	Escherichia coli	SI	Clinical_samples
One Day In Denmark	Human	2018	PRJEB37711	SAMEA6656881	ERR4013940	Escherichia coli	SI	Clinical_samples
One Day In Denmark	Human	2018	PRJEB37711	SAMEA6656882	ERR4013941	Escherichia coli	SI	Clinical_samples
One Day In Denmark	Human	2018	PRJEB37711	SAMEA6656883	ERR4013942	Enterobacter cloacae	SI	Clinical_samples
One Day In Denmark	Human	2018	PRJEB37711	SAMEA6656884	ERR4013943	Haemophilus influenzae	SI	Clinical_samples
One Day In Denmark	Human	2018	PRJEB37711	SAMEA6656885	ERR4013944	Pseudomonas putida	SI	Clinical_samples
One Day In Denmark	Human	2018	PRJEB37711	SAMEA6656886	ERR4013945	Citrobacter freundii	SI	Clinical_samples
One Day In Denmark	Human	2018	PRJEB37711	SAMEA6656887	ERR4013946	Pseudomonas aeruginosa	SI	Clinical_samples
One Day In Denmark	Human	2018	PRJEB37711	SAMEA6656888	ERR4013947	Escherichia coli	SI	Clinical_samples
One Day In Denmark	Human	2018	PRJEB37711	SAMEA6656889	ERR4013948	Klebsiella pneumoniae	SI	Clinical_samples
One Day In Denmark	Human	2018	PRJEB37711	SAMEA6656890	ERR4013949	Pseudomonas aeruginosa	SI	Clinical_samples
One Day In Denmark	Human	2018	PRJEB37711	SAMEA6656891	ERR4013950	Citrobacter koseri	SI	Clinical_samples
One Day In Denmark	Human	2018	PRJEB37711	SAMEA6656892	ERR4013951	Pseudomonas aeruginosa	SI	Clinical_samples

One Day In Denmark	Human	2018	PRJEB37711	SAMEA6656893	ERR4013952	Pseudomonas aeruginosa	SI	Clinical_samples
One Day In Denmark	Human	2018	PRJEB37711	SAMEA6656894	ERR4013953	Escherichia coli	SI	Clinical_samples
One Day In Denmark	Human	2018	PRJEB37711	SAMEA6656895	ERR4013954	Enterobacter cloacae	SI	Clinical_samples
One Day In Denmark	Human	2018	PRJEB37711	SAMEA6656896	ERR4013955	Moraxella catarrhalis	SI	Clinical_samples
One Day In Denmark	Human	2018	PRJEB37711	SAMEA6656897	ERR4013956	Pseudomonas aeruginosa	SI	Clinical_samples
One Day In Denmark	Human	2018	PRJEB37711	SAMEA6656898	ERR4013957	Proteus mirabilis	SI	Clinical_samples
One Day In Denmark	Human	2018	PRJEB37711	SAMEA6656899	ERR4013958	Escherichia coli	SI	Clinical_samples
One Day In Denmark	Human	2018	PRJEB37711	SAMEA6656900	ERR4013959	Pseudomonas aeruginosa	SI	Clinical_samples
One Day In Denmark	Human	2018	PRJEB37711	SAMEA6656901	ERR4013960	Proteus vulgaris	SI	Clinical_samples
One Day In Denmark	Human	2018	PRJEB37711	SAMEA6656902	ERR4013961	Escherichia coli	SI	Clinical_samples
One Day In Denmark	Human	2018	PRJEB37711	SAMEA6656903	ERR4013962	Escherichia coli	SI	Clinical_samples
One Day In Denmark	Human	2018	PRJEB37711	SAMEA6656904	ERR4013963	Raoultella ornithinolytica	SI	Clinical_samples
One Day In Denmark	Human	2018	PRJEB37711	SAMEA6656905	ERR4013964	Escherichia coli	SI	Clinical_samples
One Day In Denmark	Human	2018	PRJEB37711	SAMEA6656906	ERR4013965	Klebsiella oxytoca	SI	Clinical_samples
One Day In Denmark	Human	2018	PRJEB37711	SAMEA6656907	ERR4013966	Escherichia coli	SI	Clinical_samples
One Day In Denmark	Human	2018	PRJEB37711	SAMEA6656908	ERR4013967	Klebsiella pneumoniae	SI	Clinical_samples
One Day In Denmark	Human	2018	PRJEB37711	SAMEA6656909	ERR4013968	Escherichia coli	SI	Clinical_samples
One Day In Denmark	Human	2018	PRJEB37711	SAMEA6656910	ERR4013969	Escherichia coli	SI	Clinical_samples
One Day In Denmark	Human	2018	PRJEB37711	SAMEA6656911	ERR4013970	Escherichia coli	SI	Clinical_samples
One Day In Denmark	Human	2018	PRJEB37711	SAMEA6656912	ERR4013971	Escherichia coli	SI	Clinical_samples
One Day In Denmark	Human	2018	PRJEB37711	SAMEA6656913	ERR4013972	Escherichia coli	SI	Clinical_samples
One Day In Denmark	Human	2018	PRJEB37711	SAMEA6656914	ERR4013973	Escherichia coli	SI	Clinical_samples
One Day In Denmark	Human	2018	PRJEB37711	SAMEA6656915	ERR4013974	Escherichia coli	SI	Clinical_samples
One Day In Denmark	Human	2018	PRJEB37711	SAMEA6656916	ERR4013975	Citrobacter freundii	SI	Clinical_samples
One Day In Denmark	Human	2018	PRJEB37711	SAMEA6656917	ERR4013976	Escherichia coli	SI	Clinical_samples
One Day In Denmark	Human	2018	PRJEB37711	SAMEA6656918	ERR4013977	Escherichia coli	SI	Clinical_samples
One Day In Denmark	Human	2018	PRJEB37711	SAMEA6656919	ERR4013978	Klebsiella pneumoniae	SI	Clinical_samples
One Day In Denmark	Human	2018	PRJEB37711	SAMEA6656920	ERR4013979	Enterobacter hormaechei subsp. xiangf	SI	Clinical_samples
One Day In Denmark	Human	2018	PRJEB37711	SAMEA6656921	ERR4013980	Escherichia coli	SI	Clinical_samples
One Day In Denmark	Human	2018	PRJEB37711	SAMEA6656922	ERR4013981	Escherichia coli	SI	Clinical_samples
One Day In Denmark	Human	2018	PRJEB37711	SAMEA6656923	ERR4013982	Escherichia coli	SI	Clinical_samples
One Day In Denmark	Human	2018	PRJEB37711	SAMEA6656924	ERR4013983	Escherichia coli	SI	Clinical_samples
One Day In Denmark	Human	2018	PRJEB37711	SAMEA6656925	ERR4013984	Escherichia coli	SI	Clinical_samples
One Day In Denmark	Human	2018	PRJEB37711	SAMEA6656926	ERR4013985	Haemophilus influenzae	SI	Clinical_samples

One Day In Denmark	Human	2018	PRJEB37711	SAMEA6656927	ERR4013986	Proteus vulgaris	SI	Clinical_samples
One Day In Denmark	Human	2018	PRJEB37711	SAMEA6656928	ERR4013987	Haemophilus influenzae	SI	Clinical_samples
One Day In Denmark	Human	2018	PRJEB37711	SAMEA6656929	ERR4013988	Moraxella catarrhalis	SI	Clinical_samples
One Day In Denmark	Human	2018	PRJEB37711	SAMEA6656930	ERR4013989	Escherichia coli	SI	Clinical_samples
One Day In Denmark	Human	2018	PRJEB37711	SAMEA6656931	ERR4013990	Haemophilus influenzae	SI	Clinical_samples
One Day In Denmark	Human	2018	PRJEB37711	SAMEA6656932	ERR4013991	Escherichia coli	SI	Clinical_samples
One Day In Denmark	Human	2018	PRJEB37711	SAMEA6656933	ERR4013992	Escherichia coli	SI	Clinical_samples
One Day In Denmark	Human	2018	PRJEB37711	SAMEA6656934	ERR4013993	Escherichia coli	SI	Clinical_samples
One Day In Denmark	Human	2018	PRJEB37711	SAMEA6656935	ERR4013994	Escherichia coli	SI	Clinical_samples
One Day In Denmark	Human	2018	PRJEB37711	SAMEA6656936	ERR4013995	Escherichia coli	SI	Clinical_samples
One Day In Denmark	Human	2018	PRJEB37711	SAMEA6656937	ERR4013996	Escherichia coli	SI	Clinical_samples
One Day In Denmark	Human	2018	PRJEB37711	SAMEA6656938	ERR4013997	Klebsiella oxytoca	SI	Clinical_samples
One Day In Denmark	Human	2018	PRJEB37711	SAMEA6656939	ERR4013998	Escherichia coli	SI	Clinical_samples
One Day In Denmark	Human	2018	PRJEB37711	SAMEA6656940	ERR4013999	Escherichia coli	SI	Clinical_samples
One Day In Denmark	Human	2018	PRJEB37711	SAMEA6656941	ERR4014000	Escherichia coli	SI	Clinical_samples
One Day In Denmark	Human	2018	PRJEB37711	SAMEA6656942	ERR4014001	Escherichia coli	SI	Clinical_samples
One Day In Denmark	Human	2018	PRJEB37711	SAMEA6656943	ERR4014002	Moraxella catarrhalis	SI	Clinical_samples
One Day In Denmark	Human	2018	PRJEB37711	SAMEA6656944	ERR4014003	Haemophilus influenzae	SI	Clinical_samples
One Day In Denmark	Human	2018	PRJEB37711	SAMEA6656945	ERR4014004	Moraxella catarrhalis	SI	Clinical_samples
One Day In Denmark	Human	2018	PRJEB37711	SAMEA6656946	ERR4014005	Haemophilus influenzae	SI	Clinical_samples
One Day In Denmark	Human	2018	PRJEB37711	SAMEA6656947	ERR4014006	Haemophilus influenzae	SI	Clinical_samples
One Day In Denmark	Human	2018	PRJEB37711	SAMEA6656948	ERR4014007	Haemophilus influenzae	SI	Clinical_samples
One Day In Denmark	Human	2018	PRJEB37711	SAMEA6656949	ERR4014008	Haemophilus influenzae	SI	Clinical_samples
One Day In Denmark	Human	2018	PRJEB37711	SAMEA6656950	ERR4014009	Klebsiella oxytoca	SI	Clinical_samples
One Day In Denmark	Human	2018	PRJEB37711	SAMEA6656951	ERR4014010	Escherichia coli	SI	Clinical_samples
One Day In Denmark	Human	2018	PRJEB37711	SAMEA6656952	ERR4014011	Escherichia coli	SI	Clinical_samples
One Day In Denmark	Human	2018	PRJEB37711	SAMEA6656953	ERR4014012	Klebsiella pneumoniae	SI	Clinical_samples
One Day In Denmark	Human	2018	PRJEB37711	SAMEA6656954	ERR4014013	Klebsiella pneumoniae	SI	Clinical_samples
One Day In Denmark	Human	2018	PRJEB37711	SAMEA6656955	ERR4014014	Escherichia coli	SI	Clinical_samples
One Day In Denmark	Human	2018	PRJEB37711	SAMEA6656956	ERR4014015	Citrobacter koseri	SI	Clinical_samples
One Day In Denmark	Human	2018	PRJEB37711	SAMEA6656957	ERR4014016	Escherichia coli	SI	Clinical_samples
One Day In Denmark	Human	2018	PRJEB37711	SAMEA6656958	ERR4014017	Pseudomonas aeruginosa	SI	Clinical_samples
One Day In Denmark	Human	2018	PRJEB37711	SAMEA6656959	ERR4014018	Klebsiella pneumoniae	SI	Clinical_samples
One Day In Denmark	Human	2018	PRJEB37711	SAMEA6656960	ERR4014019	Pseudomonas aeruginosa	SI	Clinical_samples

One Day In Denmark	Human	2018	PRJEB37711	SAMEA6656961	ERR4014020	Escherichia coli	SI	Clinical_samples
One Day In Denmark	Human	2018	PRJEB37711	SAMEA6656962	ERR4014021	Escherichia coli	SI	Clinical_samples
One Day In Denmark	Human	2018	PRJEB37711	SAMEA6656963	ERR4014022	Klebsiella variicola	SI	Clinical_samples
One Day In Denmark	Human	2018	PRJEB37711	SAMEA6656964	ERR4014023	Klebsiella pneumoniae	SI	Clinical_samples
One Day In Denmark	Human	2018	PRJEB37711	SAMEA6656965	ERR4014024	Escherichia coli	SI	Clinical_samples
One Day In Denmark	Human	2018	PRJEB37711	SAMEA6656966	ERR4014025	Pseudomonas aeruginosa	SI	Clinical_samples
One Day In Denmark	Human	2018	PRJEB37711	SAMEA6656967	ERR4014026	Enterobacter cloacae	SI	Clinical_samples
One Day In Denmark	Human	2018	PRJEB37711	SAMEA6656968	ERR4014027	Escherichia coli	SI	Clinical_samples
One Day In Denmark	Human	2018	PRJEB37711	SAMEA6656969	ERR4014028	Citrobacter freundii	SI	Clinical_samples
One Day In Denmark	Human	2018	PRJEB37711	SAMEA6656970	ERR4014029	Escherichia coli	SI	Clinical_samples
One Day In Denmark	Human	2018	PRJEB37711	SAMEA6656971	ERR4014030	Escherichia coli	SI	Clinical_samples
One Day In Denmark	Human	2018	PRJEB37711	SAMEA6656972	ERR4014031	Haemophilus influenzae	SI	Clinical_samples
One Day In Denmark	Human	2018	PRJEB37711	SAMEA6656973	ERR4014032	Haemophilus influenzae	SI	Clinical_samples
One Day In Denmark	Human	2018	PRJEB37711	SAMEA6656974	ERR4014033	Escherichia coli	SI	Clinical_samples
One Day In Denmark	Human	2018	PRJEB37711	SAMEA6656975	ERR4014034	Escherichia coli	SI	Clinical_samples
One Day In Denmark	Human	2018	PRJEB37711	SAMEA6656976	ERR4014035	Escherichia coli	SI	Clinical_samples
One Day In Denmark	Human	2018	PRJEB37711	SAMEA6656977	ERR4014036	Escherichia coli	SI	Clinical_samples
One Day In Denmark	Human	2018	PRJEB37711	SAMEA6656978	ERR4014037	Escherichia coli	SI	Clinical_samples
One Day In Denmark	Human	2018	PRJEB37711	SAMEA6656979	ERR4014038	Klebsiella pneumoniae	SI	Clinical_samples
One Day In Denmark	Human	2018	PRJEB37711	SAMEA6656980	ERR4014039	Escherichia coli	SI	Clinical_samples
One Day In Denmark	Human	2018	PRJEB37711	SAMEA6656981	ERR4014040	Escherichia coli	SI	Clinical_samples
One Day In Denmark	Human	2018	PRJEB37711	SAMEA6656982	ERR4014041	Escherichia coli	SI	Clinical_samples
One Day In Denmark	Human	2018	PRJEB37711	SAMEA6656983	ERR4014042	Escherichia coli	SI	Clinical_samples
One Day In Denmark	Human	2018	PRJEB37711	SAMEA6656984	ERR4014043	Escherichia coli	SI	Clinical_samples
One Day In Denmark	Human	2018	PRJEB37711	SAMEA6656985	ERR4014044	Pseudomonas aeruginosa	SI	Clinical_samples
One Day In Denmark	Human	2018	PRJEB37711	SAMEA6656986	ERR4014045	Citrobacter koseri	SI	Clinical_samples
One Day In Denmark	Human	2018	PRJEB37711	SAMEA6656987	ERR4014046	Escherichia coli	SI	Clinical_samples
One Day In Denmark	Human	2018	PRJEB37711	SAMEA6656988	ERR4014047	Klebsiella pneumoniae	SI	Clinical_samples
One Day In Denmark	Human	2018	PRJEB37711	SAMEA6656989	ERR4014048	Escherichia coli	SI	Clinical_samples
One Day In Denmark	Human	2018	PRJEB37711	SAMEA6656990	ERR4014049	Escherichia coli	SI	Clinical_samples
One Day In Denmark	Human	2018	PRJEB37711	SAMEA6656991	ERR4014050	Escherichia coli	SI	Clinical_samples
One Day In Denmark	Human	2018	PRJEB37711	SAMEA6656992	ERR4014051	Escherichia coli	SI	Clinical_samples
One Day In Denmark	Human	2018	PRJEB37711	SAMEA6656993	ERR4014052	Escherichia coli	SI	Clinical_samples
One Day In Denmark	Human	2018	PRJEB37711	SAMEA6656994	ERR4014053	Klebsiella pneumoniae	SI	Clinical_samples

One Day In Denmark	Human	2018	PRJEB37711	SAMEA6656995	ERR4014054	Escherichia coli	SI	Clinical_samples
One Day In Denmark	Human	2018	PRJEB37711	SAMEA6656996	ERR4014055	Escherichia coli	SI	Clinical_samples
One Day In Denmark	Human	2018	PRJEB37711	SAMEA6656997	ERR4014056	Klebsiella pneumoniae	SI	Clinical_samples
One Day In Denmark	Human	2018	PRJEB37711	SAMEA6656998	ERR4014057	Klebsiella pneumoniae	SI	Clinical_samples
One Day In Denmark	Human	2018	PRJEB37711	SAMEA6656999	ERR4014058	Escherichia coli	SI	Clinical_samples
One Day In Denmark	Human	2018	PRJEB37711	SAMEA6657000	ERR4014059	Escherichia coli	SI	Clinical_samples
One Day In Denmark	Human	2018	PRJEB37711	SAMEA6657001	ERR4014060	Escherichia coli	SI	Clinical_samples
One Day In Denmark	Human	2018	PRJEB37711	SAMEA6657002	ERR4014061	Escherichia coli	SI	Clinical_samples
One Day In Denmark	Human	2018	PRJEB37711	SAMEA6657003	ERR4014062	Escherichia coli	SI	Clinical_samples
One Day In Denmark	Human	2018	PRJEB37711	SAMEA6657004	ERR4014063	Yersinia enterocolitica	SI	Clinical_samples
One Day In Denmark	Human	2018	PRJEB37711	SAMEA6657005	ERR4014064	Shigella sonnei	SI	Clinical_samples
One Day In Denmark	Human	2018	PRJEB37711	SAMEA6657006	ERR4014065	Escherichia coli	SI	Clinical_samples
One Day In Denmark	Human	2018	PRJEB37711	SAMEA6657007	ERR4014066	Escherichia coli	SI	Clinical_samples
One Day In Denmark	Human	2018	PRJEB37711	SAMEA6657008	ERR4014067	Escherichia coli	SI	Clinical_samples
One Day In Denmark	Human	2018	PRJEB37711	SAMEA6657009	ERR4014068	Escherichia coli	SI	Clinical_samples
One Day In Denmark	Human	2018	PRJEB37711	SAMEA6657010	ERR4014069	Bacteroides fragilis	SI	Clinical_samples
One Day In Denmark	Human	2018	PRJEB37711	SAMEA6657011	ERR4014070	Pseudomonas aeruginosa	SI	Clinical_samples
One Day In Denmark	Human	2018	PRJEB37711	SAMEA6657012	ERR4014071	Escherichia coli	SI	Clinical_samples
One Day In Denmark	Human	2018	PRJEB37711	SAMEA6657013	ERR4014072	Klebsiella pneumoniae	SI	Clinical_samples
One Day In Denmark	Human	2018	PRJEB37711	SAMEA6657014	ERR4014073	Escherichia coli	SI	Clinical_samples
One Day In Denmark	Human	2018	PRJEB37711	SAMEA6657015	ERR4014074	Escherichia coli	SI	Clinical_samples
One Day In Denmark	Human	2018	PRJEB37711	SAMEA6657016	ERR4014075	Escherichia coli	SI	Clinical_samples
One Day In Denmark	Human	2018	PRJEB37711	SAMEA6657017	ERR4014076	Escherichia coli	SI	Clinical_samples
One Day In Denmark	Human	2018	PRJEB37711	SAMEA6657018	ERR4014077	Escherichia coli	SI	Clinical_samples
One Day In Denmark	Human	2018	PRJEB37711	SAMEA6657019	ERR4014078	Escherichia coli	SI	Clinical_samples
One Day In Denmark	Human	2018	PRJEB37711	SAMEA6657020	ERR4014079	Escherichia coli	SI	Clinical_samples
One Day In Denmark	Human	2018	PRJEB37711	SAMEA6657021	ERR4014080	Escherichia coli	SI	Clinical_samples
One Day In Denmark	Human	2018	PRJEB37711	SAMEA6657022	ERR4014081	Escherichia coli	SI	Clinical_samples
One Day In Denmark	Human	2018	PRJEB37711	SAMEA6657023	ERR4014082	Escherichia coli	SI	Clinical_samples
One Day In Denmark	Human	2018	PRJEB37711	SAMEA6657024	ERR4014083	Escherichia coli	SI	Clinical_samples
One Day In Denmark	Human	2018	PRJEB37711	SAMEA6657025	ERR4014084	Escherichia coli	SI	Clinical_samples
One Day In Denmark	Human	2018	PRJEB37711	SAMEA6657026	ERR4014085	Escherichia coli	SI	Clinical_samples
One Day In Denmark	Human	2018	PRJEB37711	SAMEA6657027	ERR4014086	Escherichia coli	SI	Clinical_samples
One Day In Denmark	Human	2018	PRJEB37711	SAMEA6657028	ERR4014087	Escherichia coli	SI	Clinical_samples

One Day In Denmark	Human	2018	PRJEB37711	SAMEA6657029	ERR4014088	Pseudomonas aeruginosa	SI	Clinical_samples
One Day In Denmark	Human	2018	PRJEB37711	SAMEA6657030	ERR4014089	Escherichia coli	SI	Clinical_samples
One Day In Denmark	Human	2018	PRJEB37711	SAMEA6657031	ERR4014090	Enterobacter cloacae	SI	Clinical_samples
One Day In Denmark	Human	2018	PRJEB37711	SAMEA6657032	ERR4014091	Escherichia coli	SI	Clinical_samples
One Day In Denmark	Human	2018	PRJEB37711	SAMEA6657033	ERR4014092	Escherichia coli	SI	Clinical_samples
One Day In Denmark	Human	2018	PRJEB37711	SAMEA6657034	ERR4014093	Escherichia coli	SI	Clinical_samples
One Day In Denmark	Human	2018	PRJEB37711	SAMEA6657035	ERR4014094	Klebsiella pneumoniae	SI	Clinical_samples
One Day In Denmark	Human	2018	PRJEB37711	SAMEA6657036	ERR4014095	Escherichia coli	SI	Clinical_samples
One Day In Denmark	Human	2018	PRJEB37711	SAMEA6657037	ERR4014096	Escherichia coli	SI	Clinical_samples
One Day In Denmark	Human	2018	PRJEB37711	SAMEA6657038	ERR4014097	Escherichia coli	SI	Clinical_samples
One Day In Denmark	Human	2018	PRJEB37711	SAMEA6657039	ERR4014098	Escherichia coli	SI	Clinical_samples
One Day In Denmark	Human	2018	PRJEB37711	SAMEA6657040	ERR4014099	Escherichia coli	SI	Clinical_samples
One Day In Denmark	Human	2018	PRJEB37711	SAMEA6657041	ERR4014100	Klebsiella pneumoniae	SI	Clinical_samples
One Day In Denmark	Human	2018	PRJEB37711	SAMEA6657042	ERR4014101	Escherichia coli	SI	Clinical_samples
One Day In Denmark	Human	2018	PRJEB37711	SAMEA6657043	ERR4014102	Escherichia coli	SI	Clinical_samples
One Day In Denmark	Human	2018	PRJEB37711	SAMEA6657044	ERR4014103	Escherichia coli	SI	Clinical_samples
One Day In Denmark	Human	2018	PRJEB37711	SAMEA6657045	ERR4014104	Klebsiella pneumoniae	SI	Clinical_samples
One Day In Denmark	Human	2018	PRJEB37711	SAMEA6657046	ERR4014105	Escherichia coli	SI	Clinical_samples
One Day In Denmark	Human	2018	PRJEB37711	SAMEA6657047	ERR4014106	Proteus mirabilis	SI	Clinical_samples
One Day In Denmark	Human	2018	PRJEB37711	SAMEA6657048	ERR4014107	Escherichia coli	SI	Clinical_samples
One Day In Denmark	Human	2018	PRJEB37711	SAMEA6657049	ERR4014108	Escherichia coli	SI	Clinical_samples
One Day In Denmark	Human	2018	PRJEB37711	SAMEA6657050	ERR4014109	Escherichia coli	SI	Clinical_samples
One Day In Denmark	Human	2018	PRJEB37711	SAMEA6657051	ERR4014110	Klebsiella pneumoniae	SI	Clinical_samples
One Day In Denmark	Human	2018	PRJEB37711	SAMEA6657052	ERR4014111	Escherichia coli	SI	Clinical_samples
One Day In Denmark	Human	2018	PRJEB37711	SAMEA6657053	ERR4014112	Escherichia coli	SI	Clinical_samples
One Day In Denmark	Human	2018	PRJEB37711	SAMEA6657054	ERR4014113	Escherichia coli	SI	Clinical_samples
One Day In Denmark	Human	2018	PRJEB37711	SAMEA6657055	ERR4014114	Escherichia coli	SI	Clinical_samples
One Day In Denmark	Human	2018	PRJEB37711	SAMEA6657056	ERR4014115	Escherichia coli	SI	Clinical_samples
One Day In Denmark	Human	2018	PRJEB37711	SAMEA6657057	ERR4014116	Escherichia coli	SI	Clinical_samples
One Day In Denmark	Human	2018	PRJEB37711	SAMEA6657058	ERR4014117	Citrobacter koseri	SI	Clinical_samples
One Day In Denmark	Human	2018	PRJEB37711	SAMEA6657059	ERR4014118	Escherichia coli	SI	Clinical_samples
One Day In Denmark	Human	2018	PRJEB37711	SAMEA6657060	ERR4014119	Escherichia coli	SI	Clinical_samples
One Day In Denmark	Human	2018	PRJEB37711	SAMEA6657061	ERR4014120	Klebsiella pneumoniae	SI	Clinical_samples
One Day In Denmark	Human	2018	PRJEB37711	SAMEA6657062	ERR4014121	Klebsiella variicola	SI	Clinical_samples

One Day In Denmark	Human	2018	PRJEB37711	SAMEA6657063	ERR4014122	Escherichia coli	SI	Clinical_samples
One Day In Denmark	Human	2018	PRJEB37711	SAMEA6657064	ERR4014123	Escherichia coli	SI	Clinical_samples
One Day In Denmark	Human	2018	PRJEB37711	SAMEA6657065	ERR4014124	Escherichia coli	SI	Clinical_samples
One Day In Denmark	Human	2018	PRJEB37711	SAMEA6657066	ERR4014125	Escherichia coli	SI	Clinical_samples
One Day In Denmark	Human	2018	PRJEB37711	SAMEA6657067	ERR4014126	Escherichia coli	SI	Clinical_samples
One Day In Denmark	Human	2018	PRJEB37711	SAMEA6657068	ERR4014127	Bacteroides fragilis	SI	Clinical_samples
One Day In Denmark	Human	2018	PRJEB37711	SAMEA6657069	ERR4014128	Streptococcus dysgalactiae	SI	Clinical_samples
One Day In Denmark	Human	2018	PRJEB37711	SAMEA6657070	ERR4014129	Escherichia coli	SI	Clinical_samples
One Day In Denmark	Human	2018	PRJEB37711	SAMEA6657071	ERR4014130	Proteus vulgaris	SI	Clinical_samples
One Day In Denmark	Human	2018	PRJEB37711	SAMEA6657072	ERR4014131	Staphylococcus aureus	SI	Clinical_samples
One Day In Denmark	Human	2018	PRJEB37711	SAMEA6657073	ERR4014132	Haemophilus influenzae	SI	Clinical_samples
One Day In Denmark	Human	2018	PRJEB37711	SAMEA6657074	ERR4014133	Pasteurella multocida	SI	Clinical_samples
One Day In Denmark	Human	2018	PRJEB37711	SAMEA6657075	ERR4014134	Moraxella catarrhalis	SI	Clinical_samples
One Day In Denmark	Human	2018	PRJEB37711	SAMEA6657076	ERR4014135	Haemophilus influenzae	SI	Clinical_samples
One Day In Denmark	Human	2018	PRJEB37711	SAMEA6657077	ERR4014136	Haemophilus influenzae	SI	Clinical_samples
One Day In Denmark	Human	2018	PRJEB37711	SAMEA6657078	ERR4014137	Escherichia coli	SI	Clinical_samples
One Day In Denmark	Human	2018	PRJEB37711	SAMEA6657079	ERR4014138	Escherichia coli	SI	Clinical_samples
One Day In Denmark	Human	2018	PRJEB37711	SAMEA6657080	ERR4014139	Yersinia enterocolitica	SI	Clinical_samples
One Day In Denmark	Human	2018	PRJEB37711	SAMEA6657081	ERR4014140	Haemophilus influenzae	SI	Clinical_samples
One Day In Denmark	Human	2018	PRJEB37711	SAMEA6657082	ERR4014141	Haemophilus influenzae	SI	Clinical_samples
One Day In Denmark	Human	2018	PRJEB37711	SAMEA6657083	ERR4014142	Moraxella catarrhalis	SI	Clinical_samples
One Day In Denmark	Human	2018	PRJEB37711	SAMEA6657084	ERR4014143	Moraxella catarrhalis	SI	Clinical_samples
One Day In Denmark	Human	2018	PRJEB37711	SAMEA6657085	ERR4014144	Haemophilus influenzae	SI	Clinical_samples
One Day In Denmark	Human	2018	PRJEB37711	SAMEA6657086	ERR4014145	Haemophilus influenzae	SI	Clinical_samples
One Day In Denmark	Human	2018	PRJEB37711	SAMEA6657087	ERR4014146	Aerococcus urinae	SI	Clinical_samples
One Day In Denmark	Human	2018	PRJEB37711	SAMEA6657088	ERR4014147	Moraxella catarrhalis	SI	Clinical_samples
One Day In Denmark	Human	2018	PRJEB37711	SAMEA6657089	ERR4014148	Aerococcus urinae	SI	Clinical_samples
One Day In Denmark	Human	2018	PRJEB37711	SAMEA6657090	ERR4014149	Haemophilus influenzae	SI	Clinical_samples
One Day In Denmark	Human	2018	PRJEB37711	SAMEA6657091	ERR4014150	Haemophilus influenzae	SI	Clinical_samples
One Day In Denmark	Human	2018	PRJEB37711	SAMEA6657092	ERR4014151	Pseudomonas aeruginosa	SI	Clinical_samples
One Day In Denmark	Human	2018	PRJEB37711	SAMEA6657093	ERR4014152	Enterobacter cloacae	SI	Clinical_samples
One Day In Denmark	Human	2018	PRJEB37711	SAMEA6657094	ERR4014153	Escherichia coli	SI	Clinical_samples
One Day In Denmark	Human	2018	PRJEB37711	SAMEA6657095	ERR4014154	Klebsiella variicola	SI	Clinical_samples
One Day In Denmark	Human	2018	PRJEB37711	SAMEA6657096	ERR4014155	Proteus mirabilis	SI	Clinical_samples

One Day In Denmark	Human	2018	PRJEB37711	SAMEA6657097	ERR4014156	Klebsiella pneumoniae	SI	Clinical_samples
One Day In Denmark	Human	2018	PRJEB37711	SAMEA6657098	ERR4014157	Aeromonas caviae	SI	Clinical_samples
One Day In Denmark	Human	2018	PRJEB37711	SAMEA6657099	ERR4014158	Pasteurella canis	SI	Clinical_samples
One Day In Denmark	Human	2018	PRJEB37711	SAMEA6657100	ERR4014159	Haemophilus influenzae	SI	Clinical_samples
One Day In Denmark	Human	2018	PRJEB37711	SAMEA6657101	ERR4014160	Haemophilus influenzae	SI	Clinical_samples
One Day In Denmark	Human	2018	PRJEB37711	SAMEA6657102	ERR4014161	Haemophilus influenzae	SI	Clinical_samples
One Day In Denmark	Human	2018	PRJEB37711	SAMEA6657103	ERR4014162	Haemophilus influenzae	SI	Clinical_samples
One Day In Denmark	Human	2018	PRJEB37711	SAMEA6657104	ERR4014163	Enterobacter cloacae	SI	Clinical_samples
One Day In Denmark	Human	2018	PRJEB37711	SAMEA6657105	ERR4014164	Yersinia enterocolitica	SI	Clinical_samples
One Day In Denmark	Human	2018	PRJEB37711	SAMEA6657106	ERR4014165	Klebsiella pneumoniae	SI	Clinical_samples
One Day In Denmark	Human	2018	PRJEB37711	SAMEA6657107	ERR4014166	Escherichia coli	SI	Clinical_samples
One Day In Denmark	Human	2018	PRJEB37711	SAMEA6657108	ERR4014167	Escherichia coli	SI	Clinical_samples
One Day In Denmark	Human	2018	PRJEB37711	SAMEA6657109	ERR4014168	Escherichia coli	SI	Clinical_samples
One Day In Denmark	Human	2018	PRJEB37711	SAMEA6657110	ERR4014169	Fusobacterium necrophorum	SI	Clinical_samples
One Day In Denmark	Human	2018	PRJEB37711	SAMEA6657111	ERR4014170	Escherichia coli	SI	Clinical_samples
One Day In Denmark	Human	2018	PRJEB37711	SAMEA6657112	ERR4014171	Escherichia coli	SI	Clinical_samples
One Day In Denmark	Human	2018	PRJEB37711	SAMEA6657113	ERR4014172	Escherichia coli	SI	Clinical_samples
One Day In Denmark	Human	2018	PRJEB37711	SAMEA6657114	ERR4014173	Escherichia coli	SI	Clinical_samples
One Day In Denmark	Human	2018	PRJEB37711	SAMEA6657115	ERR4014174	Haemophilus influenzae	SI	Clinical_samples
One Day In Denmark	Human	2018	PRJEB37711	SAMEA6657116	ERR4014175	Haemophilus influenzae	SI	Clinical_samples
One Day In Denmark	Human	2018	PRJEB37711	SAMEA6657117	ERR4014176	Escherichia coli	SI	Clinical_samples
One Day In Denmark	Human	2018	PRJEB37711	SAMEA6657118	ERR4014177	Escherichia coli	SI	Clinical_samples
One Day In Denmark	Human	2018	PRJEB37711	SAMEA6657119	ERR4014178	Escherichia coli	SI	Clinical_samples
One Day In Denmark	Human	2018	PRJEB37711	SAMEA6657120	ERR4014179	Pseudomonas aeruginosa	SI	Clinical_samples
One Day In Denmark	Human	2018	PRJEB37711	SAMEA6657121	ERR4014180	Haemophilus influenzae	SI	Clinical_samples
One Day In Denmark	Human	2018	PRJEB37711	SAMEA6657122	ERR4014181	Escherichia coli	SI	Clinical_samples
One Day In Denmark	Human	2018	PRJEB37711	SAMEA6657123	ERR4014182	Escherichia coli	SI	Clinical_samples
One Day In Denmark	Human	2018	PRJEB37711	SAMEA6657124	ERR4014183	Escherichia coli	SI	Clinical_samples
One Day In Denmark	Human	2018	PRJEB37711	SAMEA6657125	ERR4014184	Klebsiella oxytoca	SI	Clinical_samples
One Day In Denmark	Human	2018	PRJEB37711	SAMEA6657126	ERR4014185	Escherichia coli	SI	Clinical_samples
One Day In Denmark	Human	2018	PRJEB37711	SAMEA6657127	ERR4014186	Proteus mirabilis	SI	Clinical_samples
One Day In Denmark	Human	2018	PRJEB37711	SAMEA6657128	ERR4014187	Escherichia coli	SI	Clinical_samples
One Day In Denmark	Human	2018	PRJEB37711	SAMEA6657129	ERR4014188	Escherichia coli	SI	Clinical_samples
One Day In Denmark	Human	2018	PRJEB37711	SAMEA6657130	ERR4014189	Escherichia coli	SI	Clinical_samples

One Day In Denmark	Human	2018	PRJEB37711	SAMEA6657131	ERR4014190	Aerococcus sanguinicola	SI	Clinical_samples
One Day In Denmark	Human	2018	PRJEB37711	SAMEA6657132	ERR4014191	Escherichia coli	SI	Clinical_samples
One Day In Denmark	Human	2018	PRJEB37711	SAMEA6657133	ERR4014192	Escherichia coli	SI	Clinical_samples
One Day In Denmark	Human	2018	PRJEB37711	SAMEA6657134	ERR4014193	Escherichia coli	SI	Clinical_samples
One Day In Denmark	Human	2018	PRJEB37711	SAMEA6657135	ERR4014194	Escherichia coli	SI	Clinical_samples
One Day In Denmark	Human	2018	PRJEB37711	SAMEA6657136	ERR4014195	Citrobacter koseri	SI	Clinical_samples
One Day In Denmark	Human	2018	PRJEB37711	SAMEA6657137	ERR4014196	Escherichia coli	SI	Clinical_samples
One Day In Denmark	Human	2018	PRJEB37711	SAMEA6657138	ERR4014197	Escherichia coli	SI	Clinical_samples
One Day In Denmark	Human	2018	PRJEB37711	SAMEA6657139	ERR4014198	Escherichia coli	SI	Clinical_samples
One Day In Denmark	Human	2018	PRJEB37711	SAMEA6657140	ERR4014199	Escherichia coli	SI	Clinical_samples
One Day In Denmark	Human	2018	PRJEB37711	SAMEA6657141	ERR4014200	Escherichia coli	SI	Clinical_samples
One Day In Denmark	Human	2018	PRJEB37711	SAMEA6657142	ERR4014201	Escherichia coli	SI	Clinical_samples
One Day In Denmark	Human	2018	PRJEB37711	SAMEA6657143	ERR4014202	Escherichia coli	SI	Clinical_samples
One Day In Denmark	Human	2018	PRJEB37711	SAMEA6657144	ERR4014203	Haemophilus influenzae	SI	Clinical_samples
One Day In Denmark	Human	2018	PRJEB37711	SAMEA6657145	ERR4014204	Enterococcus faecalis	SI	Clinical_samples
One Day In Denmark	Human	2018	PRJEB37711	SAMEA6657146	ERR4014205	Escherichia coli	SI	Clinical_samples
One Day In Denmark	Human	2018	PRJEB37711	SAMEA6657147	ERR4014206	Enterobacter cloacae	SI	Clinical_samples
One Day In Denmark	Human	2018	PRJEB37711	SAMEA6657148	ERR4014207	Escherichia coli	SI	Clinical_samples
One Day In Denmark	Human	2018	PRJEB37711	SAMEA6657149	ERR4014208	Escherichia coli	SI	Clinical_samples
One Day In Denmark	Human	2018	PRJEB37711	SAMEA6657150	ERR4014209	Escherichia coli	SI	Clinical_samples
One Day In Denmark	Human	2018	PRJEB37711	SAMEA6657151	ERR4014210	Acinetobacter baumannii	SI	Clinical_samples
One Day In Denmark	Human	2018	PRJEB37711	SAMEA6657152	ERR4014211	Escherichia coli	SI	Clinical_samples
One Day In Denmark	Human	2018	PRJEB37711	SAMEA6657153	ERR4014212	Escherichia coli	SI	Clinical_samples
One Day In Denmark	Human	2018	PRJEB37711	SAMEA6657154	ERR4014213	Escherichia coli	SI	Clinical_samples
One Day In Denmark	Human	2018	PRJEB37711	SAMEA6657155	ERR4014214	Escherichia coli	SI	Clinical_samples
One Day In Denmark	Human	2018	PRJEB37711	SAMEA6657156	ERR4014215	Proteus vulgaris	SI	Clinical_samples
One Day In Denmark	Human	2018	PRJEB37711	SAMEA6657157	ERR4014216	Escherichia coli	SI	Clinical_samples
One Day In Denmark	Human	2018	PRJEB37711	SAMEA6657158	ERR4014217	Escherichia coli	SI	Clinical_samples
One Day In Denmark	Human	2018	PRJEB37711	SAMEA6657159	ERR4014218	Escherichia coli	SI	Clinical_samples
One Day In Denmark	Human	2018	PRJEB37711	SAMEA6657160	ERR4014219	Escherichia coli	SI	Clinical_samples
One Day In Denmark	Human	2018	PRJEB37711	SAMEA6657161	ERR4014220	Klebsiella oxytoca	SI	Clinical_samples
One Day In Denmark	Human	2018	PRJEB37711	SAMEA6657162	ERR4014221	Escherichia coli	SI	Clinical_samples
One Day In Denmark	Human	2018	PRJEB37711	SAMEA6657163	ERR4014222	Escherichia coli	SI	Clinical_samples
One Day In Denmark	Human	2018	PRJEB37711	SAMEA6657164	ERR4014223	Escherichia coli	SI	Clinical_samples

One Day In Denmark	Human	2018	PRJEB37711	SAMEA6657165	ERR4014224	Escherichia coli	SI	Clinical_samples
One Day In Denmark	Human	2018	PRJEB37711	SAMEA6657166	ERR4014225	Pseudomonas aeruginosa	SI	Clinical_samples
One Day In Denmark	Human	2018	PRJEB37711	SAMEA6657167	ERR4014226	Pseudomonas aeruginosa	SI	Clinical_samples
One Day In Denmark	Human	2018	PRJEB37711	SAMEA6657168	ERR4014227	Escherichia coli	SI	Clinical_samples
One Day In Denmark	Human	2018	PRJEB37711	SAMEA6657169	ERR4014228	Yersinia enterocolitica	SI	Clinical_samples
One Day In Denmark	Human	2018	PRJEB37711	SAMEA6657170	ERR4014229	Escherichia coli	SI	Clinical_samples
One Day In Denmark	Human	2018	PRJEB37711	SAMEA6657171	ERR4014230	Klebsiella pneumoniae	SI	Clinical_samples
One Day In Denmark	Human	2018	PRJEB37711	SAMEA6657172	ERR4014231	Pseudomonas aeruginosa	SI	Clinical_samples
One Day In Denmark	Human	2018	PRJEB37711	SAMEA6657173	ERR4014232	Serratia marcescens	SI	Clinical_samples
One Day In Denmark	Human	2018	PRJEB37711	SAMEA6657174	ERR4014233	Haemophilus influenzae	SI	Clinical_samples
One Day In Denmark	Human	2018	PRJEB37711	SAMEA6657175	ERR4014234	Enterobacter cloacae	SI	Clinical_samples
One Day In Denmark	Human	2018	PRJEB37711	SAMEA6657176	ERR4014235	Haemophilus influenzae	SI	Clinical_samples
One Day In Denmark	Human	2018	PRJEB37711	SAMEA6657177	ERR4014236	Klebsiella pneumoniae	SI	Clinical_samples
One Day In Denmark	Human	2018	PRJEB37711	SAMEA6657178	ERR4014237	Citrobacter koseri	SI	Clinical_samples
One Day In Denmark	Human	2018	PRJEB37711	SAMEA6657179	ERR4014238	Citrobacter amalonaticus	SI	Clinical_samples
One Day In Denmark	Human	2018	PRJEB37711	SAMEA6657180	ERR4014239	Escherichia coli	SI	Clinical_samples
One Day In Denmark	Human	2018	PRJEB37711	SAMEA6657181	ERR4014240	Escherichia coli	SI	Clinical_samples
One Day In Denmark	Human	2018	PRJEB37711	SAMEA6657182	ERR4014241	Escherichia coli	SI	Clinical_samples
One Day In Denmark	Human	2018	PRJEB37711	SAMEA6657183	ERR4014242	Escherichia coli	SI	Clinical_samples
One Day In Denmark	Human	2018	PRJEB37711	SAMEA6657184	ERR4014243	Escherichia coli	SI	Clinical_samples
One Day In Denmark	Human	2018	PRJEB37711	SAMEA6657185	ERR4014244	Escherichia coli	SI	Clinical_samples
One Day In Denmark	Human	2018	PRJEB37711	SAMEA6657186	ERR4014245	Escherichia coli	SI	Clinical_samples
One Day In Denmark	Human	2018	PRJEB37711	SAMEA6657187	ERR4014246	Enterobacter cloacae	SI	Clinical_samples
One Day In Denmark	Human	2018	PRJEB37711	SAMEA6657188	ERR4014247	Klebsiella oxytoca	SI	Clinical_samples
One Day In Denmark	Human	2018	PRJEB37711	SAMEA6657189	ERR4014248	Klebsiella oxytoca	SI	Clinical_samples
One Day In Denmark	Human	2018	PRJEB37711	SAMEA6657190	ERR4014249	Escherichia coli	SI	Clinical_samples
One Day In Denmark	Human	2018	PRJEB37711	SAMEA6657191	ERR4014250	Escherichia coli	SI	Clinical_samples
One Day In Denmark	Human	2018	PRJEB37711	SAMEA6657192	ERR4014251	Escherichia coli	SI	Clinical_samples
One Day In Denmark	Human	2018	PRJEB37711	SAMEA6657193	ERR4014252	Citrobacter freundii	SI	Clinical_samples
One Day In Denmark	Human	2018	PRJEB37711	SAMEA6657194	ERR4014253	Citrobacter freundii	SI	Clinical_samples
One Day In Denmark	Human	2018	PRJEB37711	SAMEA6657195	ERR4014254	Escherichia coli	SI	Clinical_samples
One Day In Denmark	Human	2018	PRJEB37711	SAMEA6657196	ERR4014255	Klebsiella pneumoniae	SI	Clinical_samples
One Day In Denmark	Human	2018	PRJEB37711	SAMEA6657197	ERR4014256	Klebsiella oxytoca	SI	Clinical_samples
One Day In Denmark	Human	2018	PRJEB37711	SAMEA6657198	ERR4014257	Proteus mirabilis	SI	Clinical_samples

One Day In Denmark	Human	2018	PRJEB37711	SAMEA6657199	ERR4014258	Klebsiella aerogenes	SI	Clinical_samples
One Day In Denmark	Human	2018	PRJEB37711	SAMEA6657200	ERR4014259	Klebsiella variicola	SI	Clinical_samples
One Day In Denmark	Human	2018	PRJEB37711	SAMEA6657201	ERR4014260	Proteus mirabilis	SI	Clinical_samples
One Day In Denmark	Human	2018	PRJEB37711	SAMEA6657202	ERR4014261	Escherichia coli	SI	Clinical_samples
One Day In Denmark	Human	2018	PRJEB37711	SAMEA6657203	ERR4014262	Escherichia coli	SI	Clinical_samples
One Day In Denmark	Human	2018	PRJEB37711	SAMEA6657204	ERR4014263	Escherichia coli	SI	Clinical_samples
One Day In Denmark	Human	2018	PRJEB37711	SAMEA6657205	ERR4014264	Klebsiella pneumoniae	SI	Clinical_samples
One Day In Denmark	Human	2018	PRJEB37711	SAMEA6657206	ERR4014265	Klebsiella pneumoniae	SI	Clinical_samples
One Day In Denmark	Human	2018	PRJEB37711	SAMEA6657207	ERR4014266	Escherichia coli	SI	Clinical_samples
One Day In Denmark	Human	2018	PRJEB37711	SAMEA6657208	ERR4014267	Escherichia coli	SI	Clinical_samples
One Day In Denmark	Human	2018	PRJEB37711	SAMEA6657209	ERR4014268	Escherichia coli	SI	Clinical_samples
One Day In Denmark	Human	2018	PRJEB37711	SAMEA6657210	ERR4014269	Escherichia coli	SI	Clinical_samples
One Day In Denmark	Human	2018	PRJEB37711	SAMEA6657211	ERR4014270	Escherichia coli	SI	Clinical_samples
One Day In Denmark	Human	2018	PRJEB37711	SAMEA6657212	ERR4014271	Escherichia coli	SI	Clinical_samples
One Day In Denmark	Human	2018	PRJEB37711	SAMEA6657213	ERR4014272	Escherichia coli	SI	Clinical_samples
One Day In Denmark	Human	2018	PRJEB37711	SAMEA6657214	ERR4014273	Escherichia coli	SI	Clinical_samples
One Day In Denmark	Human	2018	PRJEB37711	SAMEA6657215	ERR4014274	Escherichia coli	SI	Clinical_samples
One Day In Denmark	Human	2018	PRJEB37711	SAMEA6657216	ERR4014275	Escherichia coli	SI	Clinical_samples
One Day In Denmark	Human	2018	PRJEB37711	SAMEA6657217	ERR4014276	Klebsiella oxytoca	SI	Clinical_samples
One Day In Denmark	Human	2018	PRJEB37711	SAMEA6657218	ERR4014277	Enterobacter cloacae	SI	Clinical_samples
One Day In Denmark	Human	2018	PRJEB37711	SAMEA6657219	ERR4014278	Pseudomonas aeruginosa	SI	Clinical_samples
One Day In Denmark	Human	2018	PRJEB37711	SAMEA6657220	ERR4014279	Escherichia coli	SI	Clinical_samples
One Day In Denmark	Human	2018	PRJEB37711	SAMEA6657221	ERR4014280	Proteus mirabilis	SI	Clinical_samples
One Day In Denmark	Human	2018	PRJEB37711	SAMEA6657222	ERR4014281	Escherichia coli	SI	Clinical_samples
One Day In Denmark	Human	2018	PRJEB37711	SAMEA6657223	ERR4014282	Escherichia coli	SI	Clinical_samples
One Day In Denmark	Human	2018	PRJEB37711	SAMEA6657224	ERR4014283	Escherichia coli	SI	Clinical_samples
One Day In Denmark	Human	2018	PRJEB37711	SAMEA6657225	ERR4014284	Escherichia coli	SI	Clinical_samples
One Day In Denmark	Human	2018	PRJEB37711	SAMEA6657226	ERR4014285	Klebsiella pneumoniae	SI	Clinical_samples
One Day In Denmark	Human	2018	PRJEB37711	SAMEA6657227	ERR4014286	Klebsiella pneumoniae	SI	Clinical_samples
One Day In Denmark	Human	2018	PRJEB37711	SAMEA6657228	ERR4014287	Proteus mirabilis	SI	Clinical_samples
One Day In Denmark	Human	2018	PRJEB37711	SAMEA6657229	ERR4014288	Escherichia coli	SI	Clinical_samples
One Day In Denmark	Human	2018	PRJEB37711	SAMEA6657230	ERR4014289	Escherichia coli	SI	Clinical_samples
One Day In Denmark	Human	2018	PRJEB37711	SAMEA6657231	ERR4014290	Citrobacter braakii	SI	Clinical_samples
One Day In Denmark	Human	2018	PRJEB37711	SAMEA6657232	ERR4014291	Proteus mirabilis	SI	Clinical_samples

One Day In Denmark	Human	2018	PRJEB37711	SAMEA6657233	ERR4014292	Klebsiella pneumoniae	SI	Clinical_samples
One Day In Denmark	Human	2018	PRJEB37711	SAMEA6657234	ERR4014293	Klebsiella aerogenes	SI	Clinical_samples
One Day In Denmark	Human	2018	PRJEB37711	SAMEA6657235	ERR4014294	Proteus mirabilis	SI	Clinical_samples
One Day In Denmark	Human	2018	PRJEB37711	SAMEA6657236	ERR4014295	Klebsiella pneumoniae	SI	Clinical_samples
One Day In Denmark	Human	2018	PRJEB37711	SAMEA6657237	ERR4014296	Enterobacter cloacae	SI	Clinical_samples
One Day In Denmark	Human	2018	PRJEB37711	SAMEA6657238	ERR4014297	Escherichia coli	SI	Clinical_samples
One Day In Denmark	Human	2018	PRJEB37711	SAMEA6657239	ERR4014298	Proteus mirabilis	SI	Clinical_samples
One Day In Denmark	Human	2018	PRJEB37711	SAMEA6657240	ERR4014299	Klebsiella pneumoniae	SI	Clinical_samples
One Day In Denmark	Human	2018	PRJEB37711	SAMEA6657241	ERR4014300	Citrobacter freundii	SI	Clinical_samples
One Day In Denmark	Human	2018	PRJEB37711	SAMEA6657242	ERR4014301	Escherichia coli	SI	Clinical_samples
One Day In Denmark	Human	2018	PRJEB37711	SAMEA6657243	ERR4014302	Klebsiella pneumoniae	SI	Clinical_samples
One Day In Denmark	Human	2018	PRJEB37711	SAMEA6657244	ERR4014303	Klebsiella pneumoniae	SI	Clinical_samples
One Day In Denmark	Human	2018	PRJEB37711	SAMEA6657245	ERR4014304	Escherichia coli	SI	Clinical_samples
One Day In Denmark	Human	2018	PRJEB37711	SAMEA6657246	ERR4014305	Proteus mirabilis	SI	Clinical_samples
One Day In Denmark	Human	2018	PRJEB37711	SAMEA6657247	ERR4014306	Escherichia coli	SI	Clinical_samples
One Day In Denmark	Human	2018	PRJEB37711	SAMEA6657248	ERR4014307	Escherichia coli	SI	Clinical_samples
One Day In Denmark	Human	2018	PRJEB37711	SAMEA6657249	ERR4014308	Escherichia coli	SI	Clinical_samples
One Day In Denmark	Human	2018	PRJEB37711	SAMEA6657250	ERR4014309	Escherichia coli	SI	Clinical_samples
One Day In Denmark	Human	2018	PRJEB37711	SAMEA6657251	ERR4014310	Escherichia coli	SI	Clinical_samples
One Day In Denmark	Human	2018	PRJEB37711	SAMEA6657252	ERR4014311	Escherichia coli	SI	Clinical_samples
One Day In Denmark	Human	2018	PRJEB37711	SAMEA6657253	ERR4014312	Escherichia coli	SI	Clinical_samples
One Day In Denmark	Human	2018	PRJEB37711	SAMEA6657254	ERR4014313	Escherichia coli	SI	Clinical_samples
One Day In Denmark	Human	2018	PRJEB37711	SAMEA6657255	ERR4014314	Escherichia coli	SI	Clinical_samples
One Day In Denmark	Human	2018	PRJEB37711	SAMEA6657256	ERR4014315	Escherichia coli	SI	Clinical_samples
One Day In Denmark	Human	2018	PRJEB37711	SAMEA6657257	ERR4014316	Escherichia coli	SI	Clinical_samples
One Day In Denmark	Human	2018	PRJEB37711	SAMEA6657258	ERR4014317	Escherichia coli	SI	Clinical_samples
One Day In Denmark	Human	2018	PRJEB37711	SAMEA6657259	ERR4014318	Escherichia coli	SI	Clinical_samples
One Day In Denmark	Human	2018	PRJEB37711	SAMEA6657260	ERR4014319	Escherichia coli	SI	Clinical_samples
One Day In Denmark	Human	2018	PRJEB37711	SAMEA6657261	ERR4014320	Escherichia coli	SI	Clinical_samples
One Day In Denmark	Human	2018	PRJEB37711	SAMEA6657262	ERR4014321	Proteus mirabilis	SI	Clinical_samples
One Day In Denmark	Human	2018	PRJEB37711	SAMEA6657263	ERR4014322	Klebsiella pneumoniae	SI	Clinical_samples
One Day In Denmark	Human	2018	PRJEB37711	SAMEA6657264	ERR4014323	Escherichia coli	SI	Clinical_samples
One Day In Denmark	Human	2018	PRJEB37711	SAMEA6657265	ERR4014324	Escherichia coli	SI	Clinical_samples
One Day In Denmark	Human	2018	PRJEB37711	SAMEA6657266	ERR4014325	Klebsiella pneumoniae	SI	Clinical_samples

One Day In Denmark	Human	2018	PRJEB37711	SAMEA6657267	ERR4014326	Escherichia coli	SI	Clinical_samples
One Day In Denmark	Human	2018	PRJEB37711	SAMEA6657268	ERR4014327	Escherichia coli	SI	Clinical_samples
One Day In Denmark	Human	2018	PRJEB37711	SAMEA6657269	ERR4014328	Escherichia coli	SI	Clinical_samples
One Day In Denmark	Human	2018	PRJEB37711	SAMEA6657270	ERR4014329	Escherichia coli	SI	Clinical_samples
One Day In Denmark	Human	2018	PRJEB37711	SAMEA6657271	ERR4014330	Escherichia coli	SI	Clinical_samples
One Day In Denmark	Human	2018	PRJEB37711	SAMEA6657272	ERR4014331	Escherichia coli	SI	Clinical_samples
One Day In Denmark	Human	2018	PRJEB37711	SAMEA6657273	ERR4014332	Klebsiella pneumoniae	SI	Clinical_samples
One Day In Denmark	Human	2018	PRJEB37711	SAMEA6657274	ERR4014333	Klebsiella pneumoniae	SI	Clinical_samples
One Day In Denmark	Human	2018	PRJEB37711	SAMEA6657275	ERR4014334	Escherichia coli	SI	Clinical_samples
One Day In Denmark	Human	2018	PRJEB37711	SAMEA6657276	ERR4014335	Escherichia coli	SI	Clinical_samples
One Day In Denmark	Human	2018	PRJEB37711	SAMEA6657277	ERR4014336	Escherichia coli	SI	Clinical_samples
One Day In Denmark	Human	2018	PRJEB37711	SAMEA6657278	ERR4014337	Escherichia coli	SI	Clinical_samples
One Day In Denmark	Human	2018	PRJEB37711	SAMEA6657279	ERR4014338	Escherichia coli	SI	Clinical_samples
One Day In Denmark	Human	2018	PRJEB37711	SAMEA6657280	ERR4014339	Escherichia coli	SI	Clinical_samples
One Day In Denmark	Human	2018	PRJEB37711	SAMEA6657281	ERR4014340	Escherichia coli	SI	Clinical_samples
One Day In Denmark	Human	2018	PRJEB37711	SAMEA6657282	ERR4014341	Klebsiella variicola	SI	Clinical_samples
One Day In Denmark	Human	2018	PRJEB37711	SAMEA6657283	ERR4014342	Klebsiella oxytoca	SI	Clinical_samples
One Day In Denmark	Human	2018	PRJEB37711	SAMEA6657284	ERR4014343	Proteus mirabilis	SI	Clinical_samples
One Day In Denmark	Human	2018	PRJEB37711	SAMEA6657285	ERR4014344	Escherichia coli	SI	Clinical_samples
One Day In Denmark	Human	2018	PRJEB37711	SAMEA6657286	ERR4014345	Escherichia coli	SI	Clinical_samples
One Day In Denmark	Human	2018	PRJEB37711	SAMEA6657287	ERR4014346	Escherichia coli	SI	Clinical_samples
One Day In Denmark	Human	2018	PRJEB37711	SAMEA6657288	ERR4014347	Escherichia coli	SI	Clinical_samples
One Day In Denmark	Human	2018	PRJEB37711	SAMEA6657289	ERR4014348	Escherichia coli	SI	Clinical_samples
One Day In Denmark	Human	2018	PRJEB37711	SAMEA6657290	ERR4014349	Klebsiella oxytoca	SI	Clinical_samples
One Day In Denmark	Human	2018	PRJEB37711	SAMEA6657291	ERR4014350	Citrobacter freundii	SI	Clinical_samples
One Day In Denmark	Human	2018	PRJEB37711	SAMEA6657292	ERR4014351	Klebsiella variicola	SI	Clinical_samples
One Day In Denmark	Human	2018	PRJEB37711	SAMEA6657293	ERR4014352	Escherichia coli	SI	Clinical_samples
One Day In Denmark	Human	2018	PRJEB37711	SAMEA6657294	ERR4014353	Escherichia coli	SI	Clinical_samples
One Day In Denmark	Human	2018	PRJEB37711	SAMEA6657295	ERR4014354	Escherichia coli	SI	Clinical_samples
One Day In Denmark	Human	2018	PRJEB37711	SAMEA6657296	ERR4014355	Escherichia coli	SI	Clinical_samples
One Day In Denmark	Human	2018	PRJEB37711	SAMEA6657297	ERR4014356	Escherichia coli	SI	Clinical_samples
One Day In Denmark	Human	2018	PRJEB37711	SAMEA6657298	ERR4014357	Escherichia coli	SI	Clinical_samples
One Day In Denmark	Human	2018	PRJEB37711	SAMEA6657299	ERR4014358	Escherichia coli	SI	Clinical_samples
One Day In Denmark	Human	2018	PRJEB37711	SAMEA6657300	ERR4014359	Escherichia coli	SI	Clinical_samples

One Day In Denmark	Human	2018	PRJEB37711	SAMEA6657301	ERR4014360	Escherichia coli	SI	Clinical_samples
One Day In Denmark	Human	2018	PRJEB37711	SAMEA6657302	ERR4014361	Citrobacter koseri	SI	Clinical_samples
One Day In Denmark	Human	2018	PRJEB37711	SAMEA6657303	ERR4014362	Klebsiella pneumoniae	SI	Clinical_samples
One Day In Denmark	Human	2018	PRJEB37711	SAMEA6657304	ERR4014363	Escherichia coli	SI	Clinical_samples
One Day In Denmark	Human	2018	PRJEB37711	SAMEA6657305	ERR4014364	Escherichia coli	SI	Clinical_samples
One Day In Denmark	Human	2018	PRJEB37711	SAMEA6657306	ERR4014365	Escherichia coli	SI	Clinical_samples
One Day In Denmark	Human	2018	PRJEB37711	SAMEA6657307	ERR4014366	Klebsiella pneumoniae	SI	Clinical_samples
One Day In Denmark	Human	2018	PRJEB37711	SAMEA6657308	ERR4014367	Proteus mirabilis	SI	Clinical_samples
One Day In Denmark	Human	2018	PRJEB37711	SAMEA6657310	ERR4014369	Escherichia coli	SI	Clinical_samples
One Day In Denmark	Human	2018	PRJEB37711	SAMEA6657311	ERR4014370	Klebsiella pneumoniae	SI	Clinical_samples
One Day In Denmark	Human	2018	PRJEB37711	SAMEA6657312	ERR4014371	Escherichia coli	SI	Clinical_samples
One Day In Denmark	Human	2018	PRJEB37711	SAMEA6657313	ERR4014372	Klebsiella pneumoniae	SI	Clinical_samples
One Day In Denmark	Human	2018	PRJEB37711	SAMEA6657314	ERR4014373	Escherichia coli	SI	Clinical_samples
One Day In Denmark	Human	2018	PRJEB37711	SAMEA6657315	ERR4014374	Escherichia coli	SI	Clinical_samples
One Day In Denmark	Human	2018	PRJEB37711	SAMEA6657316	ERR4014375	Escherichia coli	SI	Clinical_samples
One Day In Denmark	Human	2018	PRJEB37711	SAMEA6657317	ERR4014376	Escherichia coli	SI	Clinical_samples
One Day In Denmark	Human	2018	PRJEB37711	SAMEA6657318	ERR4014377	Escherichia coli	SI	Clinical_samples
One Day In Denmark	Human	2018	PRJEB37711	SAMEA6657319	ERR4014378	Escherichia coli	SI	Clinical_samples
One Day In Denmark	Human	2018	PRJEB37711	SAMEA6657320	ERR4014379	Proteus mirabilis	SI	Clinical_samples
One Day In Denmark	Human	2018	PRJEB37711	SAMEA6657321	ERR4014380	Klebsiella aerogenes	SI	Clinical_samples
One Day In Denmark	Human	2018	PRJEB37711	SAMEA6657322	ERR4014381	Escherichia coli	SI	Clinical_samples
One Day In Denmark	Human	2018	PRJEB37711	SAMEA6657323	ERR4014382	Escherichia coli	SI	Clinical_samples
One Day In Denmark	Human	2018	PRJEB37711	SAMEA6657324	ERR4014383	Proteus mirabilis	SI	Clinical_samples
One Day In Denmark	Human	2018	PRJEB37711	SAMEA6657325	ERR4014384	Klebsiella pneumoniae	SI	Clinical_samples
One Day In Denmark	Human	2018	PRJEB37711	SAMEA6657326	ERR4014385	Pseudomonas aeruginosa	SI	Clinical_samples
One Day In Denmark	Human	2018	PRJEB37711	SAMEA6657327	ERR4014386	Klebsiella pneumoniae	SI	Clinical_samples
One Day In Denmark	Human	2018	PRJEB37711	SAMEA6657328	ERR4014387	Escherichia coli	SI	Clinical_samples
One Day In Denmark	Human	2018	PRJEB37711	SAMEA6657329	ERR4014388	Escherichia coli	SI	Clinical_samples
One Day In Denmark	Human	2018	PRJEB37711	SAMEA6657330	ERR4014389	Klebsiella pneumoniae	SI	Clinical_samples
One Day In Denmark	Human	2018	PRJEB37711	SAMEA6657331	ERR4014390	Escherichia coli	SI	Clinical_samples
One Day In Denmark	Human	2018	PRJEB37711	SAMEA6657332	ERR4014391	Escherichia coli	SI	Clinical_samples
One Day In Denmark	Human	2018	PRJEB37711	SAMEA6657333	ERR4014392	Escherichia coli	SI	Clinical_samples
One Day In Denmark	Human	2018	PRJEB37711	SAMEA6657334	ERR4014393	Salmonella enterica	SI	Clinical_samples
One Day In Denmark	Human	2018	PRJEB37711	SAMEA6657335	ERR4014394	Escherichia coli	SI	Clinical_samples

One Day In Denmark	Human	2018	PRJEB37711	SAMEA6657336	ERR4014395	Escherichia coli	SI	Clinical_samples
One Day In Denmark	Human	2018	PRJEB37711	SAMEA6657337	ERR4014396	Escherichia coli	SI	Clinical_samples
One Day In Denmark	Human	2018	PRJEB37711	SAMEA6657338	ERR4014397	Escherichia coli	SI	Clinical_samples
One Day In Denmark	Human	2018	PRJEB37711	SAMEA6657339	ERR4014398	Escherichia coli	SI	Clinical_samples
One Day In Denmark	Human	2018	PRJEB37711	SAMEA6657340	ERR4014399	Escherichia coli	SI	Clinical_samples
One Day In Denmark	Human	2018	PRJEB37711	SAMEA6657341	ERR4014400	Escherichia coli	SI	Clinical_samples
One Day In Denmark	Human	2018	PRJEB37711	SAMEA6657342	ERR4014401	Escherichia coli	SI	Clinical_samples
One Day In Denmark	Human	2018	PRJEB37711	SAMEA6657343	ERR4014402	Escherichia coli	SI	Clinical_samples
One Day In Denmark	Human	2018	PRJEB37711	SAMEA6657344	ERR4014403	Escherichia coli	SI	Clinical_samples
One Day In Denmark	Human	2018	PRJEB37711	SAMEA6657345	ERR4014404	Escherichia coli	SI	Clinical_samples
One Day In Denmark	Human	2018	PRJEB37711	SAMEA6657346	ERR4014405	Escherichia coli	SI	Clinical_samples
One Day In Denmark	Human	2018	PRJEB37711	SAMEA6657347	ERR4014406	Escherichia coli	SI	Clinical_samples
One Day In Denmark	Human	2018	PRJEB37711	SAMEA6657348	ERR4014407	Escherichia coli	SI	Clinical_samples
One Day In Denmark	Human	2018	PRJEB37711	SAMEA6657349	ERR4014408	Escherichia coli	SI	Clinical_samples
One Day In Denmark	Human	2018	PRJEB37711	SAMEA6657350	ERR4014409	Escherichia coli	SI	Clinical_samples
One Day In Denmark	Human	2018	PRJEB37711	SAMEA6657351	ERR4014410	Proteus mirabilis	SI	Clinical_samples
One Day In Denmark	Human	2018	PRJEB37711	SAMEA6657352	ERR4014411	Klebsiella pneumoniae	SI	Clinical_samples
One Day In Denmark	Human	2018	PRJEB37711	SAMEA6657353	ERR4014412	Proteus mirabilis	SI	Clinical_samples
One Day In Denmark	Human	2018	PRJEB37711	SAMEA6657354	ERR4014413	Proteus mirabilis	SI	Clinical_samples
One Day In Denmark	Human	2018	PRJEB37711	SAMEA6657355	ERR4014414	Pseudomonas aeruginosa	SI	Clinical_samples
One Day In Denmark	Human	2018	PRJEB37711	SAMEA6657356	ERR4014415	Klebsiella pneumoniae	SI	Clinical_samples
One Day In Denmark	Human	2018	PRJEB37711	SAMEA6657357	ERR4014416	Escherichia coli	SI	Clinical_samples
One Day In Denmark	Human	2018	PRJEB37711	SAMEA6657358	ERR4014417	Escherichia coli	SI	Clinical_samples
One Day In Denmark	Human	2018	PRJEB37711	SAMEA6657359	ERR4014418	Escherichia coli	SI	Clinical_samples
One Day In Denmark	Human	2018	PRJEB37711	SAMEA6657360	ERR4014419	Escherichia coli	SI	Clinical_samples
One Day In Denmark	Human	2018	PRJEB37711	SAMEA6657361	ERR4014420	Escherichia coli	SI	Clinical_samples
One Day In Denmark	Human	2018	PRJEB37711	SAMEA6657362	ERR4014421	Escherichia coli	SI	Clinical_samples
One Day In Denmark	Human	2018	PRJEB37711	SAMEA6657363	ERR4014422	Escherichia coli	SI	Clinical_samples
One Day In Denmark	Human	2018	PRJEB37711	SAMEA6657364	ERR4014423	Escherichia coli	SI	Clinical_samples
One Day In Denmark	Human	2018	PRJEB37711	SAMEA6657365	ERR4014424	Escherichia coli	SI	Clinical_samples
One Day In Denmark	Human	2018	PRJEB37711	SAMEA6657366	ERR4014425	Escherichia coli	SI	Clinical_samples
One Day In Denmark	Human	2018	PRJEB37711	SAMEA6657367	ERR4014426	Escherichia coli	SI	Clinical_samples
One Day In Denmark	Human	2018	PRJEB37711	SAMEA6657368	ERR4014427	Escherichia coli	SI	Clinical_samples
One Day In Denmark	Human	2018	PRJEB37711	SAMEA6657369	ERR4014428	Escherichia coli	SI	Clinical_samples

One Day In Denmark	Human	2018	PRJEB37711	SAMEA6657370	ERR4014429	Escherichia coli	SI	Clinical_samples
One Day In Denmark	Human	2018	PRJEB37711	SAMEA6657371	ERR4014430	Escherichia coli	SI	Clinical_samples
One Day In Denmark	Human	2018	PRJEB37711	SAMEA6657372	ERR4014431	Proteus mirabilis	SI	Clinical_samples
One Day In Denmark	Human	2018	PRJEB37711	SAMEA6657373	ERR4014432	Escherichia coli	SI	Clinical_samples
One Day In Denmark	Human	2018	PRJEB37711	SAMEA6657374	ERR4014433	Escherichia coli	SI	Clinical_samples
One Day In Denmark	Human	2018	PRJEB37711	SAMEA6657375	ERR4014434	Escherichia coli	SI	Clinical_samples
One Day In Denmark	Human	2018	PRJEB37711	SAMEA6657376	ERR4014435	Escherichia coli	SI	Clinical_samples
One Day In Denmark	Human	2018	PRJEB37711	SAMEA6657377	ERR4014436	Proteus mirabilis	SI	Clinical_samples
One Day In Denmark	Human	2018	PRJEB37711	SAMEA6657378	ERR4014437	Klebsiella pneumoniae	SI	Clinical_samples
One Day In Denmark	Human	2018	PRJEB37711	SAMEA6657379	ERR4014438	Providencia rettgeri	SI	Clinical_samples
One Day In Denmark	Human	2018	PRJEB37711	SAMEA6657380	ERR4014439	Escherichia coli	SI	Clinical_samples
One Day In Denmark	Human	2018	PRJEB37711	SAMEA6657381	ERR4014440	Escherichia coli	SI	Clinical_samples
One Day In Denmark	Human	2018	PRJEB37711	SAMEA6657382	ERR4014441	Escherichia coli	SI	Clinical_samples
One Day In Denmark	Human	2018	PRJEB37711	SAMEA6657383	ERR4014442	Escherichia coli	SI	Clinical_samples
One Day In Denmark	Human	2018	PRJEB37711	SAMEA6657384	ERR4014443	Escherichia coli	SI	Clinical_samples
One Day In Denmark	Human	2018	PRJEB37711	SAMEA6657385	ERR4014444	Escherichia coli	SI	Clinical_samples
One Day In Denmark	Human	2018	PRJEB37711	SAMEA6657386	ERR4014445	Escherichia coli	SI	Clinical_samples
One Day In Denmark	Human	2018	PRJEB37711	SAMEA6657387	ERR4014446	Escherichia coli	SI	Clinical_samples
One Day In Denmark	Human	2018	PRJEB37711	SAMEA6657388	ERR4014447	Escherichia coli	SI	Clinical_samples
One Day In Denmark	Human	2018	PRJEB37711	SAMEA6657389	ERR4014448	Escherichia coli	SI	Clinical_samples
One Day In Denmark	Human	2018	PRJEB37711	SAMEA6657390	ERR4014449	Escherichia coli	SI	Clinical_samples
One Day In Denmark	Human	2018	PRJEB37711	SAMEA6657391	ERR4014450	Escherichia coli	SI	Clinical_samples
One Day In Denmark	Human	2018	PRJEB37711	SAMEA6657392	ERR4014451	Escherichia coli	SI	Clinical_samples
One Day In Denmark	Human	2018	PRJEB37711	SAMEA6657393	ERR4014452	Escherichia coli	SI	Clinical_samples
One Day In Denmark	Human	2018	PRJEB37711	SAMEA6657394	ERR4014453	Escherichia coli	SI	Clinical_samples
One Day In Denmark	Human	2018	PRJEB37711	SAMEA6657395	ERR4014454	Escherichia coli	SI	Clinical_samples
One Day In Denmark	Human	2018	PRJEB37711	SAMEA6657396	ERR4014455	Escherichia coli	SI	Clinical_samples
One Day In Denmark	Human	2018	PRJEB37711	SAMEA6657397	ERR4014456	Escherichia coli	SI	Clinical_samples
One Day In Denmark	Human	2018	PRJEB37711	SAMEA6657398	ERR4014457	Escherichia coli	SI	Clinical_samples
One Day In Denmark	Human	2018	PRJEB37711	SAMEA6657399	ERR4014458	Escherichia coli	SI	Clinical_samples
One Day In Denmark	Human	2018	PRJEB37711	SAMEA6657400	ERR4014459	Escherichia coli	SI	Clinical_samples
One Day In Denmark	Human	2018	PRJEB37711	SAMEA6657401	ERR4014460	Escherichia coli	SI	Clinical_samples
One Day In Denmark	Human	2018	PRJEB37711	SAMEA6657402	ERR4014461	Escherichia coli	SI	Clinical_samples
One Day In Denmark	Human	2018	PRJEB37711	SAMEA6657403	ERR4014462	Neisseria gonorrhoeae	SI	Clinical_samples

One Day In Denmark	Human	2018	PRJEB37711	SAMEA6657404	ERR4014463	Klebsiella pneumoniae	SI	Clinical_samples
One Day In Denmark	Human	2018	PRJEB37711	SAMEA6657405	ERR4014464	Escherichia coli	SI	Clinical_samples
One Day In Denmark	Human	2018	PRJEB37711	SAMEA6657406	ERR4014465	Escherichia coli	SI	Clinical_samples
One Day In Denmark	Human	2018	PRJEB37711	SAMEA6657407	ERR4014466	Escherichia coli	SI	Clinical_samples
One Day In Denmark	Human	2018	PRJEB37711	SAMEA6657408	ERR4014467	Escherichia coli	SI	Clinical_samples
One Day In Denmark	Human	2018	PRJEB37711	SAMEA6657409	ERR4014468	Proteus vulgaris	SI	Clinical_samples
One Day In Denmark	Human	2018	PRJEB37711	SAMEA6657410	ERR4014469	Klebsiella pneumoniae	SI	Clinical_samples
One Day In Denmark	Human	2018	PRJEB37711	SAMEA6657411	ERR4014470	Pseudomonas aeruginosa	SI	Clinical_samples
One Day In Denmark	Human	2018	PRJEB37711	SAMEA6657412	ERR4014471	Moraxella catarrhalis	SI	Clinical_samples
One Day In Denmark	Human	2018	PRJEB37711	SAMEA6657413	ERR4014472	Escherichia coli	SI	Clinical_samples
One Day In Denmark	Human	2018	PRJEB37711	SAMEA6657414	ERR4014473	Escherichia coli	SI	Clinical_samples
One Day In Denmark	Human	2018	PRJEB37711	SAMEA6657415	ERR4014474	Proteus vulgaris	SI	Clinical_samples
One Day In Denmark	Human	2018	PRJEB37711	SAMEA6657416	ERR4014475	Escherichia coli	SI	Clinical_samples
One Day In Denmark	Human	2018	PRJEB37711	SAMEA6657417	ERR4014476	Escherichia coli	SI	Clinical_samples
One Day In Denmark	Human	2018	PRJEB37711	SAMEA6657419	ERR4014477	Citrobacter koseri	SI	Clinical_samples
One Day In Denmark	Human	2018	PRJEB37711	SAMEA6657420	ERR4014478	Escherichia coli	SI	Clinical_samples
One Day In Denmark	Human	2018	PRJEB37711	SAMEA6657421	ERR4014479	Escherichia coli	SI	Clinical_samples
One Day In Denmark	Human	2018	PRJEB37711	SAMEA6657422	ERR4014480	Klebsiella pneumoniae	SI	Clinical_samples
One Day In Denmark	Human	2018	PRJEB37711	SAMEA6657423	ERR4014481	Escherichia coli	SI	Clinical_samples
One Day In Denmark	Human	2018	PRJEB37711	SAMEA6657424	ERR4014482	Escherichia coli	SI	Clinical_samples
One Day In Denmark	Human	2018	PRJEB37711	SAMEA6657425	ERR4014483	Klebsiella pneumoniae	SI	Clinical_samples
One Day In Denmark	Human	2018	PRJEB37711	SAMEA6657426	ERR4014484	Escherichia coli	SI	Clinical_samples
One Day In Denmark	Human	2018	PRJEB37711	SAMEA6657427	ERR4014485	Escherichia coli	SI	Clinical_samples
One Day In Denmark	Human	2018	PRJEB37711	SAMEA6657428	ERR4014486	Proteus mirabilis	SI	Clinical_samples
One Day In Denmark	Human	2018	PRJEB37711	SAMEA6657429	ERR4014487	Proteus mirabilis	SI	Clinical_samples
One Day In Denmark	Human	2018	PRJEB37711	SAMEA6657430	ERR4014488	Escherichia coli	SI	Clinical_samples
One Day In Denmark	Human	2018	PRJEB37711	SAMEA6657431	ERR4014489	Citrobacter koseri	SI	Clinical_samples
One Day In Denmark	Human	2018	PRJEB37711	SAMEA6657432	ERR4014490	Escherichia coli	SI	Clinical_samples
One Day In Denmark	Human	2018	PRJEB37711	SAMEA6657433	ERR4014491	Escherichia coli	SI	Clinical_samples
One Day In Denmark	Human	2018	PRJEB37711	SAMEA6657434	ERR4014492	Escherichia coli	SI	Clinical_samples
One Day In Denmark	Human	2018	PRJEB37711	SAMEA6657435	ERR4014493	Escherichia coli	SI	Clinical_samples
One Day In Denmark	Human	2018	PRJEB37711	SAMEA6657436	ERR4014494	Escherichia coli	SI	Clinical_samples
One Day In Denmark	Human	2018	PRJEB37711	SAMEA6657437	ERR4014495	Escherichia coli	SI	Clinical_samples
One Day In Denmark	Human	2018	PRJEB37711	SAMEA6657438	ERR4014496	Escherichia coli	SI	Clinical_samples

One Day In Denmark	Human	2018	PRJEB37711	SAMEA6657439	ERR4014497	Escherichia coli	SI	Clinical_samples
One Day In Denmark	Human	2018	PRJEB37711	SAMEA6657440	ERR4014498	Proteus vulgaris	SI	Clinical_samples
One Day In Denmark	Human	2018	PRJEB37711	SAMEA6657441	ERR4014499	Klebsiella oxytoca	SI	Clinical_samples
One Day In Denmark	Human	2018	PRJEB37711	SAMEA6657442	ERR4014500	Klebsiella pneumoniae	SI	Clinical_samples
One Day In Denmark	Human	2018	PRJEB37711	SAMEA6657443	ERR4014501	Klebsiella aerogenes	SI	Clinical_samples
One Day In Denmark	Human	2018	PRJEB37711	SAMEA6657444	ERR4014502	Moraxella catarrhalis	SI	Clinical_samples
One Day In Denmark	Human	2018	PRJEB37711	SAMEA6657445	ERR4014503	Haemophilus influenzae	SI	Clinical_samples
One Day In Denmark	Human	2018	PRJEB37711	SAMEA6657446	ERR4014504	Escherichia coli	SI	Clinical_samples
One Day In Denmark	Human	2018	PRJEB37711	SAMEA6657447	ERR4014505	Pseudomonas aeruginosa	SI	Clinical_samples
One Day In Denmark	Human	2018	PRJEB37711	SAMEA6657448	ERR4014506	Escherichia coli	SI	Clinical_samples
One Day In Denmark	Human	2018	PRJEB37711	SAMEA6657449	ERR4014507	Escherichia coli	SI	Clinical_samples
One Day In Denmark	Human	2018	PRJEB37711	SAMEA6657450	ERR4014508	Escherichia coli	SI	Clinical_samples
One Day In Denmark	Human	2018	PRJEB37711	SAMEA6657451	ERR4014509	Escherichia coli	SI	Clinical_samples
One Day In Denmark	Human	2018	PRJEB37711	SAMEA6657452	ERR4014510	Escherichia coli	SI	Clinical_samples
One Day In Denmark	Human	2018	PRJEB37711	SAMEA6657453	ERR4014511	Escherichia coli	SI	Clinical_samples
One Day In Denmark	Human	2018	PRJEB37711	SAMEA6657454	ERR4014512	Escherichia coli	SI	Clinical_samples
One Day In Denmark	Human	2018	PRJEB37711	SAMEA6657455	ERR4014513	Escherichia coli	SI	Clinical_samples
One Day In Denmark	Human	2018	PRJEB37711	SAMEA6657456	ERR4014514	Pseudomonas aeruginosa	SI	Clinical_samples
One Day In Denmark	Human	2018	PRJEB37711	SAMEA6657457	ERR4014515	Escherichia coli	SI	Clinical_samples
One Day In Denmark	Human	2018	PRJEB37711	SAMEA6657458	ERR4014516	Proteus mirabilis	SI	Clinical_samples
One Day In Denmark	Human	2018	PRJEB37711	SAMEA6657459	ERR4014517	Escherichia coli	SI	Clinical_samples
One Day In Denmark	Human	2018	PRJEB37711	SAMEA6657460	ERR4014518	Escherichia coli	SI	Clinical_samples
One Day In Denmark	Human	2018	PRJEB37711	SAMEA6657461	ERR4014519	Proteus mirabilis	SI	Clinical_samples
One Day In Denmark	Human	2018	PRJEB37711	SAMEA6657462	ERR4014520	Pseudomonas aeruginosa	SI	Clinical_samples
One Day In Denmark	Human	2018	PRJEB37711	SAMEA6657463	ERR4014521	Escherichia coli	SI	Clinical_samples
One Day In Denmark	Human	2018	PRJEB37711	SAMEA6657464	ERR4014522	Escherichia coli	SI	Clinical_samples
One Day In Denmark	Human	2018	PRJEB37711	SAMEA6657465	ERR4014523	Escherichia coli	SI	Clinical_samples
One Day In Denmark	Human	2018	PRJEB37711	SAMEA6657466	ERR4014524	Escherichia coli	SI	Clinical_samples
One Day In Denmark	Human	2018	PRJEB37711	SAMEA6657467	ERR4014525	Escherichia coli	SI	Clinical_samples
One Day In Denmark	Human	2018	PRJEB37711	SAMEA6657468	ERR4014526	Klebsiella variicola	SI	Clinical_samples
One Day In Denmark	Human	2018	PRJEB37711	SAMEA6657469	ERR4014527	Klebsiella pneumoniae	SI	Clinical_samples
One Day In Denmark	Human	2018	PRJEB37711	SAMEA6657470	ERR4014528	Escherichia coli	SI	Clinical_samples
One Day In Denmark	Human	2018	PRJEB37711	SAMEA6657471	ERR4014529	Escherichia coli	SI	Clinical_samples
One Day In Denmark	Human	2018	PRJEB37711	SAMEA6657472	ERR4014530	Escherichia coli	SI	Clinical_samples

One Day In Denmark	Human	2018	PRJEB37711	SAMEA6657473	ERR4014531	Escherichia coli	SI	Clinical_samples
One Day In Denmark	Human	2018	PRJEB37711	SAMEA6657474	ERR4014532	Escherichia coli	SI	Clinical_samples
One Day In Denmark	Human	2018	PRJEB37711	SAMEA6657475	ERR4014533	Escherichia coli	SI	Clinical_samples
One Day In Denmark	Human	2018	PRJEB37711	SAMEA6657476	ERR4014534	Escherichia coli	SI	Clinical_samples
One Day In Denmark	Human	2018	PRJEB37711	SAMEA6657477	ERR4014535	Enterobacter cloacae	SI	Clinical_samples
One Day In Denmark	Human	2018	PRJEB37711	SAMEA6657478	ERR4014536	Klebsiella oxytoca	SI	Clinical_samples
One Day In Denmark	Human	2018	PRJEB37711	SAMEA6657479	ERR4014537	Escherichia coli	SI	Clinical_samples
One Day In Denmark	Human	2018	PRJEB37711	SAMEA6657480	ERR4014538	Escherichia coli	SI	Clinical_samples
One Day In Denmark	Human	2018	PRJEB37711	SAMEA6657481	ERR4014539	Escherichia coli	SI	Clinical_samples
One Day In Denmark	Human	2018	PRJEB37711	SAMEA6657482	ERR4014540	Escherichia coli	SI	Clinical_samples
One Day In Denmark	Human	2018	PRJEB37711	SAMEA6657483	ERR4014541	Escherichia coli	SI	Clinical_samples
One Day In Denmark	Human	2018	PRJEB37711	SAMEA6657484	ERR4014542	Haemophilus influenzae	SI	Clinical_samples
One Day In Denmark	Human	2018	PRJEB37711	SAMEA6657485	ERR4014543	Acinetobacter bereziniae	SI	Clinical_samples
One Day In Denmark	Human	2018	PRJEB37711	SAMEA6657486	ERR4014544	Haemophilus influenzae	SI	Clinical_samples
One Day In Denmark	Human	2018	PRJEB37711	SAMEA6657487	ERR4014545	Escherichia coli	SI	Clinical_samples
One Day In Denmark	Human	2018	PRJEB37711	SAMEA6657488	ERR4014546	Escherichia coli	SI	Clinical_samples
One Day In Denmark	Human	2018	PRJEB37711	SAMEA6657489	ERR4014547	Escherichia coli	SI	Clinical_samples
One Day In Denmark	Human	2018	PRJEB37711	SAMEA6657490	ERR4014548	Escherichia coli	SI	Clinical_samples
One Day In Denmark	Human	2018	PRJEB37711	SAMEA6657491	ERR4014549	Escherichia coli	SI	Clinical_samples
One Day In Denmark	Human	2018	PRJEB37711	SAMEA6657492	ERR4014550	Escherichia coli	SI	Clinical_samples
One Day In Denmark	Human	2018	PRJEB37711	SAMEA6657493	ERR4014551	Escherichia coli	SI	Clinical_samples
One Day In Denmark	Human	2018	PRJEB37711	SAMEA6657494	ERR4014552	Escherichia coli	SI	Clinical_samples
One Day In Denmark	Human	2018	PRJEB37711	SAMEA6657495	ERR4014553	Campylobacter jejuni	SI	Clinical_samples
One Day In Denmark	Human	2018	PRJEB37711	SAMEA6657496	ERR4014554	Campylobacter jejuni	SI	Clinical_samples
One Day In Denmark	Human	2018	PRJEB37711	SAMEA6657497	ERR4014555	Bacteroides fragilis	SI	Clinical_samples
One Day In Denmark	Human	2018	PRJEB37711	SAMEA6657498	ERR4014556	Escherichia coli	SI	Clinical_samples
One Day In Denmark	Human	2018	PRJEB37711	SAMEA6657499	ERR4014557	Escherichia coli	SI	Clinical_samples
One Day In Denmark	Human	2018	PRJEB37711	SAMEA6657500	ERR4014558	Escherichia coli	SI	Clinical_samples
One Day In Denmark	Human	2018	PRJEB37711	SAMEA6657501	ERR4014559	Escherichia coli	SI	Clinical_samples
One Day In Denmark	Human	2018	PRJEB37711	SAMEA6657502	ERR4014560	Escherichia coli	SI	Clinical_samples
One Day In Denmark	Human	2018	PRJEB37711	SAMEA6657503	ERR4014561	Escherichia coli	SI	Clinical_samples
One Day In Denmark	Human	2018	PRJEB37711	SAMEA6657504	ERR4014562	Klebsiella pneumoniae	SI	Clinical_samples
One Day In Denmark	Human	2018	PRJEB37711	SAMEA6657505	ERR4014563	Haemophilus influenzae	SI	Clinical_samples
One Day In Denmark	Human	2018	PRJEB37711	SAMEA6657506	ERR4014564	Haemophilus influenzae	SI	Clinical_samples

One Day In Denmark	Human	2018	PRJEB37711	SAMEA6657507	ERR4014565	Escherichia coli	SI	Clinical_samples
One Day In Denmark	Human	2018	PRJEB37711	SAMEA6657508	ERR4014566	Citrobacter freundii	SI	Clinical_samples
One Day In Denmark	Human	2018	PRJEB37711	SAMEA6657509	ERR4014567	Citrobacter koseri	SI	Clinical_samples
One Day In Denmark	Human	2018	PRJEB37711	SAMEA6657510	ERR4014568	Proteus vulgaris	SI	Clinical_samples
One Day In Denmark	Human	2018	PRJEB37711	SAMEA6657511	ERR4014569	Escherichia coli	SI	Clinical_samples
One Day In Denmark	Human	2018	PRJEB37711	SAMEA6657512	ERR4014570	Escherichia coli	SI	Clinical_samples
One Day In Denmark	Human	2018	PRJEB37711	SAMEA6657513	ERR4014571	Escherichia coli	SI	Clinical_samples
One Day In Denmark	Human	2018	PRJEB37711	SAMEA6657514	ERR4014572	Escherichia coli	SI	Clinical_samples
One Day In Denmark	Human	2018	PRJEB37711	SAMEA6657515	ERR4014573	Escherichia coli	SI	Clinical_samples
One Day In Denmark	Human	2018	PRJEB37711	SAMEA6657516	ERR4014574	Escherichia coli	SI	Clinical_samples
One Day In Denmark	Human	2018	PRJEB37711	SAMEA6657517	ERR4014575	Escherichia coli	SI	Clinical_samples
One Day In Denmark	Human	2018	PRJEB37711	SAMEA6657518	ERR4014576	Escherichia coli	SI	Clinical_samples
One Day In Denmark	Human	2018	PRJEB37711	SAMEA6657519	ERR4014577	Escherichia coli	SI	Clinical_samples
One Day In Denmark	Human	2018	PRJEB37711	SAMEA6657520	ERR4014578	Proteus mirabilis	SI	Clinical_samples
One Day In Denmark	Human	2018	PRJEB37711	SAMEA6657521	ERR4014579	Escherichia coli	SI	Clinical_samples
One Day In Denmark	Human	2018	PRJEB37711	SAMEA6657522	ERR4014580	Escherichia coli	SI	Clinical_samples
One Day In Denmark	Human	2018	PRJEB37711	SAMEA6657523	ERR4014581	Escherichia coli	SI	Clinical_samples
One Day In Denmark	Human	2018	PRJEB37711	SAMEA6657524	ERR4014582	Escherichia coli	SI	Clinical_samples
One Day In Denmark	Human	2018	PRJEB37711	SAMEA6657525	ERR4014583	Escherichia coli	SI	Clinical_samples
One Day In Denmark	Human	2018	PRJEB37711	SAMEA6657526	ERR4014584	Escherichia coli	SI	Clinical_samples
One Day In Denmark	Human	2018	PRJEB37711	SAMEA6657527	ERR4014585	Escherichia coli	SI	Clinical_samples
One Day In Denmark	Human	2018	PRJEB37711	SAMEA6657528	ERR4014586	Escherichia coli	SI	Clinical_samples
One Day In Denmark	Human	2018	PRJEB37711	SAMEA6657529	ERR4014587	Escherichia coli	SI	Clinical_samples
One Day In Denmark	Human	2018	PRJEB37711	SAMEA6657530	ERR4014588	Escherichia coli	SI	Clinical_samples
One Day In Denmark	Human	2018	PRJEB37711	SAMEA6657531	ERR4014589	Pseudomonas aeruginosa	SI	Clinical_samples
One Day In Denmark	Human	2018	PRJEB37711	SAMEA6657532	ERR4014590	Escherichia coli	SI	Clinical_samples
One Day In Denmark	Human	2018	PRJEB37711	SAMEA6657533	ERR4014591	Klebsiella aerogenes	SI	Clinical_samples
One Day In Denmark	Human	2018	PRJEB37711	SAMEA6657534	ERR4014592	Escherichia coli	SI	Clinical_samples
One Day In Denmark	Human	2018	PRJEB37711	SAMEA6657535	ERR4014593	Escherichia coli	SI	Clinical_samples
One Day In Denmark	Human	2018	PRJEB37711	SAMEA6657536	ERR4014594	Escherichia coli	SI	Clinical_samples
One Day In Denmark	Human	2018	PRJEB37711	SAMEA6657537	ERR4014595	Escherichia coli	SI	Clinical_samples
One Day In Denmark	Human	2018	PRJEB37711	SAMEA6657538	ERR4014596	Escherichia coli	SI	Clinical_samples
One Day In Denmark	Human	2018	PRJEB37711	SAMEA6657539	ERR4014597	Escherichia coli	SI	Clinical_samples
One Day In Denmark	Human	2018	PRJEB37711	SAMEA6657540	ERR4014598	Klebsiella pneumoniae	SI	Clinical_samples

One Day In Denmark	Human	2018	PRJEB37711	SAMEA6657541	ERR4014599	Escherichia coli	SI	Clinical_samples
One Day In Denmark	Human	2018	PRJEB37711	SAMEA6657542	ERR4014600	Citrobacter koseri	SI	Clinical_samples
One Day In Denmark	Human	2018	PRJEB37711	SAMEA6657543	ERR4014601	Pseudomonas aeruginosa	SI	Clinical_samples
One Day In Denmark	Human	2018	PRJEB37711	SAMEA6657544	ERR4014602	Escherichia coli	SI	Clinical_samples
One Day In Denmark	Human	2018	PRJEB37711	SAMEA6657545	ERR4014603	Escherichia coli	SI	Clinical_samples
One Day In Denmark	Human	2018	PRJEB37711	SAMEA6657546	ERR4014604	Escherichia coli	SI	Clinical_samples
One Day In Denmark	Human	2018	PRJEB37711	SAMEA6657547	ERR4014605	Escherichia coli	SI	Clinical_samples
One Day In Denmark	Human	2018	PRJEB37711	SAMEA6657548	ERR4014606	Escherichia coli	SI	Clinical_samples
One Day In Denmark	Human	2018	PRJEB37711	SAMEA6657549	ERR4014607	Escherichia coli	SI	Clinical_samples
One Day In Denmark	Human	2018	PRJEB37711	SAMEA6657550	ERR4014608	Escherichia coli	SI	Clinical_samples
One Day In Denmark	Human	2018	PRJEB37711	SAMEA6657551	ERR4014609	Escherichia coli	SI	Clinical_samples
One Day In Denmark	Human	2018	PRJEB37711	SAMEA6657552	ERR4014610	Escherichia coli	SI	Clinical_samples
One Day In Denmark	Human	2018	PRJEB37711	SAMEA6657553	ERR4014611	Haemophilus influenzae	SI	Clinical_samples
One Day In Denmark	Human	2018	PRJEB37711	SAMEA6657554	ERR4014612	Haemophilus influenzae	SI	Clinical_samples
One Day In Denmark	Human	2018	PRJEB37711	SAMEA6657555	ERR4014613	Escherichia coli	SI	Clinical_samples
One Day In Denmark	Human	2018	PRJEB37711	SAMEA6657556	ERR4014614	Escherichia coli	SI	Clinical_samples
One Day In Denmark	Human	2018	PRJEB37711	SAMEA6657557	ERR4014615	Salmonella enterica	SI	Clinical_samples
One Day In Denmark	Human	2018	PRJEB37711	SAMEA6657558	ERR4014616	Escherichia coli	SI	Clinical_samples
One Day In Denmark	Human	2018	PRJEB37711	SAMEA6657559	ERR4014617	Pseudomonas aeruginosa	SI	Clinical_samples
One Day In Denmark	Human	2018	PRJEB37711	SAMEA6657560	ERR4014618	Escherichia coli	SI	Clinical_samples
One Day In Denmark	Human	2018	PRJEB37711	SAMEA6657561	ERR4014619	Klebsiella pneumoniae	SI	Clinical_samples
One Day In Denmark	Human	2018	PRJEB37711	SAMEA6657562	ERR4014620	Escherichia coli	SI	Clinical_samples
One Day In Denmark	Human	2018	PRJEB37711	SAMEA6657563	ERR4014621	Escherichia coli	SI	Clinical_samples
One Day In Denmark	Human	2018	PRJEB37711	SAMEA6657564	ERR4014622	Klebsiella pneumoniae	SI	Clinical_samples
One Day In Denmark	Human	2018	PRJEB37711	SAMEA6657565	ERR4014623	Klebsiella pneumoniae	SI	Clinical_samples
One Day In Denmark	Human	2018	PRJEB37711	SAMEA6657566	ERR4014624	Escherichia coli	SI	Clinical_samples
One Day In Denmark	Human	2018	PRJEB37711	SAMEA6657567	ERR4014625	Pseudomonas aeruginosa	SI	Clinical_samples
One Day In Denmark	Human	2018	PRJEB37711	SAMEA6657568	ERR4014626	Escherichia coli	SI	Clinical_samples
One Day In Denmark	Human	2018	PRJEB37711	SAMEA6657569	ERR4014627	Pseudomonas aeruginosa	SI	Clinical_samples
One Day In Denmark	Human	2018	PRJEB37711	SAMEA6657570	ERR4014628	Moraxella catarrhalis	SI	Clinical_samples
One Day In Denmark	Human	2018	PRJEB37711	SAMEA6657571	ERR4014629	Proteus vulgaris	SI	Clinical_samples
One Day In Denmark	Human	2018	PRJEB37711	SAMEA6657572	ERR4014630	Proteus vulgaris	SI	Clinical_samples
One Day In Denmark	Human	2018	PRJEB37711	SAMEA6657573	ERR4014631	Klebsiella oxytoca	SI	Clinical_samples
One Day In Denmark	Human	2018	PRJEB37711	SAMEA6657574	ERR4014632	Haemophilus influenzae	SI	Clinical_samples

One Day In Denmark	Human	2018	PRJEB37711	SAMEA6657575	ERR4014633	Pseudomonas aeruginosa	SI	Clinical_samples
One Day In Denmark	Human	2018	PRJEB37711	SAMEA6657576	ERR4014634	Escherichia coli	SI	Clinical_samples
One Day In Denmark	Human	2018	PRJEB37711	SAMEA6657577	ERR4014635	Proteus mirabilis	SI	Clinical_samples
One Day In Denmark	Human	2018	PRJEB37711	SAMEA6657578	ERR4014636	Citrobacter koseri	SI	Clinical_samples
One Day In Denmark	Human	2018	PRJEB37711	SAMEA6657579	ERR4014637	Escherichia coli	SI	Clinical_samples
One Day In Denmark	Human	2018	PRJEB37711	SAMEA6657580	ERR4014638	Prevotella baroniae	SI	Clinical_samples
One Day In Denmark	Human	2018	PRJEB37711	SAMEA6657581	ERR4014639	Enterobacter cloacae	SI	Clinical_samples
One Day In Denmark	Human	2018	PRJEB37711	SAMEA6657582	ERR4014640	Salmonella enterica	SI	Clinical_samples
One Day In Denmark	Human	2018	PRJEB37711	SAMEA6657583	ERR4014641	Salmonella enterica	SI	Clinical_samples
One Day In Denmark	Human	2018	PRJEB37711	SAMEA6657584	ERR4014642	Stenotrophomonas maltophilia	SI	Clinical_samples
One Day In Denmark	Human	2018	PRJEB37711	SAMEA6657585	ERR4014643	Stenotrophomonas maltophilia	SI	Clinical_samples
One Day In Denmark	Human	2018	PRJEB37711	SAMEA6657586	ERR4014644	Haemophilus influenzae	SI	Clinical_samples
One Day In Denmark	Human	2018	PRJEB37711	SAMEA6657587	ERR4014645	Serratia marcescens	SI	Clinical_samples
One Day In Denmark	Human	2018	PRJEB37711	SAMEA6657588	ERR4014646	Serratia marcescens	SI	Clinical_samples
One Day In Denmark	Human	2018	PRJEB37711	SAMEA6657589	ERR4014647	Klebsiella pneumoniae	SI	Clinical_samples
One Day In Denmark	Human	2018	PRJEB37711	SAMEA6657590	ERR4014648	Serratia marcescens	SI	Clinical_samples
One Day In Denmark	Human	2018	PRJEB37711	SAMEA6657591	ERR4014649	Serratia marcescens	SI	Clinical_samples
One Day In Denmark	Human	2018	PRJEB37711	SAMEA6657592	ERR4014650	Stenotrophomonas maltophilia	SI	Clinical_samples
One Day In Denmark	Human	2018	PRJEB37711	SAMEA6657593	ERR4014651	Escherichia coli	SI	Clinical_samples
One Day In Denmark	Human	2018	PRJEB37711	SAMEA6657594	ERR4014652	Escherichia coli	SI	Clinical_samples
One Day In Denmark	Human	2018	PRJEB37711	SAMEA6657595	ERR4014653	Escherichia coli	SI	Clinical_samples
One Day In Denmark	Human	2018	PRJEB37711	SAMEA6657596	ERR4014654	Escherichia coli	SI	Clinical_samples
One Day In Denmark	Human	2018	PRJEB37711	SAMEA6657597	ERR4014655	Escherichia coli	SI	Clinical_samples
One Day In Denmark	Human	2018	PRJEB37711	SAMEA6657598	ERR4014656	Escherichia coli	SI	Clinical_samples
One Day In Denmark	Human	2018	PRJEB37711	SAMEA6657599	ERR4014657	Escherichia coli	SI	Clinical_samples
One Day In Denmark	Human	2018	PRJEB37711	SAMEA6657600	ERR4014658	Escherichia coli	SI	Clinical_samples
One Day In Denmark	Human	2018	PRJEB37711	SAMEA6657601	ERR4014659	Haemophilus influenzae	SI	Clinical_samples
One Day In Denmark	Human	2018	PRJEB37711	SAMEA6657602	ERR4014660	Escherichia coli	SI	Clinical_samples
One Day In Denmark	Human	2018	PRJEB37711	SAMEA6657603	ERR4014661	Escherichia coli	SI	Clinical_samples
One Day In Denmark	Human	2018	PRJEB37711	SAMEA6657604	ERR4014662	Escherichia coli	SI	Clinical_samples
One Day In Denmark	Human	2018	PRJEB37711	SAMEA6657605	ERR4014663	Pasteurella dagmatis	SI	Clinical_samples
One Day In Denmark	Human	2018	PRJEB37711	SAMEA6657606	ERR4014664	Klebsiella pneumoniae	SI	Clinical_samples
One Day In Denmark	Human	2018	PRJEB37711	SAMEA6657607	ERR4014665	Raoultella ornithinolytica	SI	Clinical_samples
One Day In Denmark	Human	2018	PRJEB37711	SAMEA6657608	ERR4014666	Escherichia coli	SI	Clinical_samples

One Day In Denmark	Human	2018	PRJEB37711	SAMEA6657609	ERR4014667	Pseudomonas aeruginosa	SI	Clinical_samples
One Day In Denmark	Human	2018	PRJEB37711	SAMEA6657610	ERR4014668	Citrobacter freundii	SI	Clinical_samples
One Day In Denmark	Human	2018	PRJEB37711	SAMEA6657611	ERR4014669	Klebsiella aerogenes	SI	Clinical_samples
One Day In Denmark	Human	2018	PRJEB37711	SAMEA6657612	ERR4014670	Klebsiella pneumoniae	SI	Clinical_samples
One Day In Denmark	Human	2018	PRJEB37711	SAMEA6657613	ERR4014671	Klebsiella pneumoniae	SI	Clinical_samples
One Day In Denmark	Human	2018	PRJEB37711	SAMEA6657614	ERR4014672	Escherichia coli	SI	Clinical_samples
One Day In Denmark	Human	2018	PRJEB37711	SAMEA6657615	ERR4014673	Escherichia coli	SI	Clinical_samples
One Day In Denmark	Human	2018	PRJEB37711	SAMEA6657616	ERR4014674	Escherichia coli	SI	Clinical_samples
One Day In Denmark	Human	2018	PRJEB37711	SAMEA6657617	ERR4014675	Proteus mirabilis	SI	Clinical_samples
One Day In Denmark	Human	2018	PRJEB37711	SAMEA6657618	ERR4014676	Klebsiella pneumoniae	SI	Clinical_samples
One Day In Denmark	Human	2018	PRJEB37711	SAMEA6657619	ERR4014677	Escherichia coli	SI	Clinical_samples
One Day In Denmark	Human	2018	PRJEB37711	SAMEA6657620	ERR4014678	Haemophilus influenzae	SI	Clinical_samples
One Day In Denmark	Human	2018	PRJEB37711	SAMEA6657621	ERR4014679	Escherichia coli	SI	Clinical_samples
One Day In Denmark	Human	2018	PRJEB37711	SAMEA6657622	ERR4014680	Escherichia coli	SI	Clinical_samples
One Day In Denmark	Human	2018	PRJEB37711	SAMEA6657623	ERR4014681	Haemophilus influenzae	SI	Clinical_samples
One Day In Denmark	Human	2018	PRJEB37711	SAMEA6657624	ERR4014682	Escherichia coli	SI	Clinical_samples
One Day In Denmark	Human	2018	PRJEB37711	SAMEA6657625	ERR4014683	Pseudomonas aeruginosa	SI	Clinical_samples
One Day In Denmark	Human	2018	PRJEB37711	SAMEA6657626	ERR4014684	Escherichia coli	SI	Clinical_samples
One Day In Denmark	Human	2018	PRJEB37711	SAMEA6657627	ERR4014685	Escherichia coli	SI	Clinical_samples
One Day In Denmark	Human	2018	PRJEB37711	SAMEA6657628	ERR4014686	Escherichia coli	SI	Clinical_samples
One Day In Denmark	Human	2018	PRJEB37711	SAMEA6657629	ERR4014687	Acinetobacter pittii	SI	Clinical_samples
One Day In Denmark	Human	2018	PRJEB37711	SAMEA6657630	ERR4014688	Escherichia coli	SI	Clinical_samples
One Day In Denmark	Human	2018	PRJEB37711	SAMEA6657631	ERR4014689	Proteus vulgaris	SI	Clinical_samples
One Day In Denmark	Human	2018	PRJEB37711	SAMEA6657632	ERR4014690	Escherichia coli	SI	Clinical_samples
One Day In Denmark	Human	2018	PRJEB37711	SAMEA6657633	ERR4014691	Citrobacter koseri	SI	Clinical_samples
One Day In Denmark	Human	2018	PRJEB37711	SAMEA6657634	ERR4014692	Klebsiella oxytoca	SI	Clinical_samples
One Day In Denmark	Human	2018	PRJEB37711	SAMEA6657635	ERR4014693	Haemophilus influenzae	SI	Clinical_samples
One Day In Denmark	Human	2018	PRJEB37711	SAMEA6657636	ERR4014694	Klebsiella pneumoniae	SI	Clinical_samples
One Day In Denmark	Human	2018	PRJEB37711	SAMEA6657637	ERR4014695	Escherichia coli	SI	Clinical_samples
One Day In Denmark	Human	2018	PRJEB37711	SAMEA6657638	ERR4014696	Escherichia coli	SI	Clinical_samples
One Day In Denmark	Human	2018	PRJEB37711	SAMEA6657639	ERR4014697	Enterococcus faecalis	SI	Clinical_samples
One Day In Denmark	Human	2018	PRJEB37711	SAMEA6657640	ERR4014698	Escherichia coli	SI	Clinical_samples
One Day In Denmark	Human	2018	PRJEB37711	SAMEA6657641	ERR4014699	Escherichia coli	SI	Clinical_samples
One Day In Denmark	Human	2018	PRJEB37711	SAMEA6657642	ERR4014700	Escherichia coli	SI	Clinical_samples

One Day In Denmark	Human	2018	PRJEB37711	SAMEA6657643	ERR4014701	Klebsiella pneumoniae	SI	Clinical_samples
One Day In Denmark	Human	2018	PRJEB37711	SAMEA6657644	ERR4014702	Escherichia coli	SI	Clinical_samples
One Day In Denmark	Human	2018	PRJEB37711	SAMEA6657645	ERR4014703	Proteus mirabilis	SI	Clinical_samples
One Day In Denmark	Human	2018	PRJEB37711	SAMEA6657646	ERR4014704	Escherichia coli	SI	Clinical_samples
One Day In Denmark	Human	2018	PRJEB37711	SAMEA6657647	ERR4014705	Klebsiella pneumoniae	SI	Clinical_samples
One Day In Denmark	Human	2018	PRJEB37711	SAMEA6657648	ERR4014706	Proteus mirabilis	SI	Clinical_samples
One Day In Denmark	Human	2018	PRJEB37711	SAMEA6657649	ERR4014707	Escherichia coli	SI	Clinical_samples
One Day In Denmark	Human	2018	PRJEB37711	SAMEA6657650	ERR4014708	Proteus vulgaris	SI	Clinical_samples
One Day In Denmark	Human	2018	PRJEB37711	SAMEA6657651	ERR4014709	Moraxella catarrhalis	SI	Clinical_samples
One Day In Denmark	Human	2018	PRJEB37711	SAMEA6657652	ERR4014710	Escherichia coli	SI	Clinical_samples
One Day In Denmark	Human	2018	PRJEB37711	SAMEA6657653	ERR4014711	Proteus mirabilis	SI	Clinical_samples
One Day In Denmark	Human	2018	PRJEB37711	SAMEA6657654	ERR4014712	Pseudomonas aeruginosa	SI	Clinical_samples
One Day In Denmark	Human	2018	PRJEB37711	SAMEA6657655	ERR4014713	Klebsiella pneumoniae	SI	Clinical_samples
One Day In Denmark	Human	2018	PRJEB37711	SAMEA6657656	ERR4014714	Escherichia coli	SI	Clinical_samples
One Day In Denmark	Human	2018	PRJEB37711	SAMEA6657657	ERR4014715	Escherichia coli	SI	Clinical_samples
One Day In Denmark	Human	2018	PRJEB37711	SAMEA6657658	ERR4014716	Escherichia coli	SI	Clinical_samples
One Day In Denmark	Human	2018	PRJEB37711	SAMEA6657659	ERR4014717	Escherichia coli	SI	Clinical_samples
One Day In Denmark	Human	2018	PRJEB37711	SAMEA6657660	ERR4014718	Escherichia coli	SI	Clinical_samples
One Day In Denmark	Human	2018	PRJEB37711	SAMEA6657661	ERR4014719	Escherichia coli	SI	Clinical_samples
One Day In Denmark	Human	2018	PRJEB37711	SAMEA6657662	ERR4014720	Escherichia coli	SI	Clinical_samples
One Day In Denmark	Human	2018	PRJEB37711	SAMEA6657663	ERR4014721	Escherichia coli	SI	Clinical_samples
One Day In Denmark	Human	2018	PRJEB37711	SAMEA6657664	ERR4014722	Klebsiella variicola	SI	Clinical_samples
One Day In Denmark	Human	2018	PRJEB37711	SAMEA6657665	ERR4014723	Escherichia coli	SI	Clinical_samples
One Day In Denmark	Human	2018	PRJEB37711	SAMEA6657666	ERR4014724	Klebsiella pneumoniae	SI	Clinical_samples
One Day In Denmark	Human	2018	PRJEB37711	SAMEA6657667	ERR4014725	Klebsiella pneumoniae	SI	Clinical_samples
One Day In Denmark	Human	2018	PRJEB37711	SAMEA6657668	ERR4014726	Escherichia coli	SI	Clinical_samples
One Day In Denmark	Human	2018	PRJEB37711	SAMEA6657669	ERR4014727	Escherichia coli	SI	Clinical_samples
One Day In Denmark	Human	2018	PRJEB37711	SAMEA6657670	ERR4014728	Escherichia coli	SI	Clinical_samples
One Day In Denmark	Human	2018	PRJEB37711	SAMEA6657671	ERR4014729	Enterococcus faecalis	SI	Clinical_samples
One Day In Denmark	Human	2018	PRJEB37711	SAMEA6657672	ERR4014730	Escherichia coli	SI	Clinical_samples
One Day In Denmark	Human	2018	PRJEB37711	SAMEA6657673	ERR4014731	Escherichia coli	SI	Clinical_samples
One Day In Denmark	Human	2018	PRJEB37711	SAMEA6657674	ERR4014732	Escherichia coli	SI	Clinical_samples
One Day In Denmark	Human	2018	PRJEB37711	SAMEA6657675	ERR4014733	Escherichia coli	SI	Clinical_samples
One Day In Denmark	Human	2018	PRJEB37711	SAMEA6657676	ERR4014734	Escherichia coli	SI	Clinical_samples

One Day In Denmark	Human	2018	PRJEB37711	SAMEA6657677	ERR4014735	Escherichia coli	SI	Clinical_samples
One Day In Denmark	Human	2018	PRJEB37711	SAMEA6657678	ERR4014736	Escherichia coli	SI	Clinical_samples
One Day In Denmark	Human	2018	PRJEB37711	SAMEA6657679	ERR4014737	Escherichia coli	SI	Clinical_samples
One Day In Denmark	Human	2018	PRJEB37711	SAMEA6657680	ERR4014738	Escherichia coli	SI	Clinical_samples
One Day In Denmark	Human	2018	PRJEB37711	SAMEA6657681	ERR4014739	Escherichia coli	SI	Clinical_samples
One Day In Denmark	Human	2018	PRJEB37711	SAMEA6657682	ERR4014740	Escherichia coli	SI	Clinical_samples
One Day In Denmark	Human	2018	PRJEB37711	SAMEA6657683	ERR4014741	Klebsiella pneumoniae	SI	Clinical_samples
One Day In Denmark	Human	2018	PRJEB37711	SAMEA6657684	ERR4014742	Cutibacterium acnes	SI	Clinical_samples
One Day In Denmark	Human	2018	PRJEB37711	SAMEA6657685	ERR4014743	Bacteroides fragilis	SI	Clinical_samples
One Day In Denmark	Human	2018	PRJEB37711	SAMEA6657686	ERR4014744	Escherichia coli	SI	Clinical_samples
One Day In Denmark	Human	2018	PRJEB37711	SAMEA6657687	ERR4014745	Enterococcus faecalis	SI	Clinical_samples
One Day In Denmark	Human	2018	PRJEB37711	SAMEA6657688	ERR4014746	Streptococcus dysgalactiae	SI	Clinical_samples
One Day In Denmark	Human	2018	PRJEB37711	SAMEA6657689	ERR4014747	Staphylococcus aureus	SI	Clinical_samples
One Day In Denmark	Human	2018	PRJEB37711	SAMEA6657690	ERR4014748	Staphylococcus aureus	SI	Clinical_samples
One Day In Denmark	Human	2018	PRJEB37711	SAMEA6657691	ERR4014749	Staphylococcus aureus	SI	Clinical_samples
One Day In Denmark	Human	2018	PRJEB37711	SAMEA6657692	ERR4014750	Staphylococcus aureus	SI	Clinical_samples
One Day In Denmark	Human	2018	PRJEB37711	SAMEA6657693	ERR4014751	Streptococcus gordonii	SI	Clinical_samples
One Day In Denmark	Human	2018	PRJEB37711	SAMEA6657694	ERR4014752	Enterococcus faecium	SI	Clinical_samples
One Day In Denmark	Human	2018	PRJEB37711	SAMEA6657695	ERR4014753	Streptococcus agalactiae	SI	Clinical_samples
One Day In Denmark	Human	2018	PRJEB37711	SAMEA6657696	ERR4014754	Staphylococcus aureus	SI	Clinical_samples
One Day In Denmark	Human	2018	PRJEB37711	SAMEA6657697	ERR4014755	Staphylococcus aureus	SI	Clinical_samples
One Day In Denmark	Human	2018	PRJEB37711	SAMEA6657698	ERR4014756	Streptococcus constellatus	SI	Clinical_samples
One Day In Denmark	Human	2018	PRJEB37711	SAMEA6657699	ERR4014757	Staphylococcus aureus	SI	Clinical_samples
One Day In Denmark	Human	2018	PRJEB37711	SAMEA6657700	ERR4014758	Staphylococcus aureus	SI	Clinical_samples
One Day In Denmark	Human	2018	PRJEB37711	SAMEA6657701	ERR4014759	Staphylococcus aureus	SI	Clinical_samples
One Day In Denmark	Human	2018	PRJEB37711	SAMEA6657702	ERR4014760	Staphylococcus aureus	SI	Clinical_samples
One Day In Denmark	Human	2018	PRJEB37711	SAMEA6657703	ERR4014761	Streptococcus agalactiae	SI	Clinical_samples
One Day In Denmark	Human	2018	PRJEB37711	SAMEA6657704	ERR4014762	Staphylococcus aureus	SI	Clinical_samples
One Day In Denmark	Human	2018	PRJEB37711	SAMEA6657705	ERR4014763	Streptococcus pyogenes	SI	Clinical_samples
One Day In Denmark	Human	2018	PRJEB37711	SAMEA6657706	ERR4014764	Streptococcus pyogenes	SI	Clinical_samples
One Day In Denmark	Human	2018	PRJEB37711	SAMEA6657707	ERR4014765	Streptococcus dysgalactiae	SI	Clinical_samples
One Day In Denmark	Human	2018	PRJEB37711	SAMEA6657708	ERR4014766	Streptococcus dysgalactiae	SI	Clinical_samples
One Day In Denmark	Human	2018	PRJEB37711	SAMEA6657709	ERR4014767	Streptococcus agalactiae	SI	Clinical_samples
One Day In Denmark	Human	2018	PRJEB37711	SAMEA6657710	ERR4014768	Streptococcus pyogenes	SI	Clinical_samples

One Day In Denmark	Human	2018	PRJEB37711	SAMEA6657711	ERR4014769	Streptococcus agalactiae	SI	Clinical_samples
One Day In Denmark	Human	2018	PRJEB37711	SAMEA6657712	ERR4014770	Staphylococcus aureus	SI	Clinical_samples
One Day In Denmark	Human	2018	PRJEB37711	SAMEA6657713	ERR4014771	Streptococcus agalactiae	SI	Clinical_samples
One Day In Denmark	Human	2018	PRJEB37711	SAMEA6657714	ERR4014772	Staphylococcus aureus	SI	Clinical_samples
One Day In Denmark	Human	2018	PRJEB37711	SAMEA6657715	ERR4014773	Staphylococcus aureus	SI	Clinical_samples
One Day In Denmark	Human	2018	PRJEB37711	SAMEA6657716	ERR4014774	Streptococcus dysgalactiae	SI	Clinical_samples
One Day In Denmark	Human	2018	PRJEB37711	SAMEA6657717	ERR4014775	Staphylococcus aureus	SI	Clinical_samples
One Day In Denmark	Human	2018	PRJEB37711	SAMEA6657718	ERR4014776	Streptococcus dysgalactiae	SI	Clinical_samples
One Day In Denmark	Human	2018	PRJEB37711	SAMEA6657719	ERR4014777	Streptococcus pyogenes	SI	Clinical_samples
One Day In Denmark	Human	2018	PRJEB37711	SAMEA6657720	ERR4014778	Streptococcus agalactiae	SI	Clinical_samples
One Day In Denmark	Human	2018	PRJEB37711	SAMEA6657721	ERR4014779	Staphylococcus aureus	SI	Clinical_samples
One Day In Denmark	Human	2018	PRJEB37711	SAMEA6657722	ERR4014780	Staphylococcus argenteus	SI	Clinical_samples
One Day In Denmark	Human	2018	PRJEB37711	SAMEA6657723	ERR4014781	Staphylococcus aureus	SI	Clinical_samples
One Day In Denmark	Human	2018	PRJEB37711	SAMEA6657724	ERR4014782	Staphylococcus aureus	SI	Clinical_samples
One Day In Denmark	Human	2018	PRJEB37711	SAMEA6657725	ERR4014783	Staphylococcus aureus	SI	Clinical_samples
One Day In Denmark	Human	2018	PRJEB37711	SAMEA6657726	ERR4014784	Streptococcus dysgalactiae	SI	Clinical_samples
One Day In Denmark	Human	2018	PRJEB37711	SAMEA6657727	ERR4014785	Staphylococcus aureus	SI	Clinical_samples
One Day In Denmark	Human	2018	PRJEB37711	SAMEA6657728	ERR4014786	Staphylococcus aureus	SI	Clinical_samples
One Day In Denmark	Human	2018	PRJEB37711	SAMEA6657729	ERR4014787	Staphylococcus aureus	SI	Clinical_samples
One Day In Denmark	Human	2018	PRJEB37711	SAMEA6657730	ERR4014788	Staphylococcus aureus	SI	Clinical_samples
One Day In Denmark	Human	2018	PRJEB37711	SAMEA6657731	ERR4014789	Streptococcus agalactiae	SI	Clinical_samples
One Day In Denmark	Human	2018	PRJEB37711	SAMEA6657732	ERR4014790	Streptococcus agalactiae	SI	Clinical_samples
One Day In Denmark	Human	2018	PRJEB37711	SAMEA6657733	ERR4014791	Streptococcus agalactiae	SI	Clinical_samples
One Day In Denmark	Human	2018	PRJEB37711	SAMEA6657734	ERR4014792	Streptococcus agalactiae	SI	Clinical_samples
One Day In Denmark	Human	2018	PRJEB37711	SAMEA6657735	ERR4014793	Staphylococcus aureus	SI	Clinical_samples
One Day In Denmark	Human	2018	PRJEB37711	SAMEA6657736	ERR4014794	Staphylococcus aureus	SI	Clinical_samples
One Day In Denmark	Human	2018	PRJEB37711	SAMEA6657737	ERR4014795	Streptococcus agalactiae	SI	Clinical_samples
One Day In Denmark	Human	2018	PRJEB37711	SAMEA6657738	ERR4014796	Streptococcus dysgalactiae	SI	Clinical_samples
One Day In Denmark	Human	2018	PRJEB37711	SAMEA6657739	ERR4014797	Staphylococcus aureus	SI	Clinical_samples
One Day In Denmark	Human	2018	PRJEB37711	SAMEA6657740	ERR4014798	Streptococcus dysgalactiae	SI	Clinical_samples
One Day In Denmark	Human	2018	PRJEB37711	SAMEA6657741	ERR4014799	Streptococcus pyogenes	SI	Clinical_samples
One Day In Denmark	Human	2018	PRJEB37711	SAMEA6657742	ERR4014800	Streptococcus pyogenes	SI	Clinical_samples
One Day In Denmark	Human	2018	PRJEB37711	SAMEA6657743	ERR4014801	Staphylococcus lugdunensis	SI	Clinical_samples
One Day In Denmark	Human	2018	PRJEB37711	SAMEA6657744	ERR4014802	Streptococcus agalactiae	SI	Clinical_samples

One Day In Denmark	Human	2018	PRJEB37711	SAMEA6657745	ERR4014803	Streptococcus agalactiae	SI	Clinical_samples
One Day In Denmark	Human	2018	PRJEB37711	SAMEA6657746	ERR4014804	Streptococcus agalactiae	SI	Clinical_samples
One Day In Denmark	Human	2018	PRJEB37711	SAMEA6657747	ERR4014805	Streptococcus agalactiae	SI	Clinical_samples
One Day In Denmark	Human	2018	PRJEB37711	SAMEA6657748	ERR4014806	Streptococcus pyogenes	SI	Clinical_samples
One Day In Denmark	Human	2018	PRJEB37711	SAMEA6657749	ERR4014807	Staphylococcus aureus	SI	Clinical_samples
One Day In Denmark	Human	2018	PRJEB37711	SAMEA6657750	ERR4014808	Staphylococcus aureus	SI	Clinical_samples
One Day In Denmark	Human	2018	PRJEB37711	SAMEA6657751	ERR4014809	Staphylococcus aureus	SI	Clinical_samples
One Day In Denmark	Human	2018	PRJEB37711	SAMEA6657752	ERR4014810	Streptococcus pyogenes	SI	Clinical_samples
One Day In Denmark	Human	2018	PRJEB37711	SAMEA6657753	ERR4014811	Enterococcus faecium	SI	Clinical_samples
One Day In Denmark	Human	2018	PRJEB37711	SAMEA6657754	ERR4014812	Staphylococcus epidermidis	SI	Clinical_samples
One Day In Denmark	Human	2018	PRJEB37711	SAMEA6657755	ERR4014813	Staphylococcus hominis	SI	Clinical_samples
One Day In Denmark	Human	2018	PRJEB37711	SAMEA6657756	ERR4014814	Enterococcus faecium	SI	Clinical_samples
One Day In Denmark	Human	2018	PRJEB37711	SAMEA6657757	ERR4014815	Enterococcus faecium	SI	Clinical_samples
One Day In Denmark	Human	2018	PRJEB37711	SAMEA6657758	ERR4014816	Enterococcus faecium	SI	Clinical_samples
One Day In Denmark	Human	2018	PRJEB37711	SAMEA6657759	ERR4014817	Staphylococcus cohnii	SI	Clinical_samples
One Day In Denmark	Human	2018	PRJEB37711	SAMEA6657760	ERR4014818	Streptococcus pneumoniae	SI	Clinical_samples
One Day In Denmark	Human	2018	PRJEB37711	SAMEA6657761	ERR4014819	Staphylococcus aureus	SI	Clinical_samples
One Day In Denmark	Human	2018	PRJEB37711	SAMEA6657762	ERR4014820	Staphylococcus capitis	SI	Clinical_samples
One Day In Denmark	Human	2018	PRJEB37711	SAMEA6657763	ERR4014821	Streptococcus gordonii	SI	Clinical_samples
One Day In Denmark	Human	2018	PRJEB37711	SAMEA6657764	ERR4014822	Staphylococcus aureus	SI	Clinical_samples
One Day In Denmark	Human	2018	PRJEB37711	SAMEA6657765	ERR4014823	Staphylococcus aureus	SI	Clinical_samples
One Day In Denmark	Human	2018	PRJEB37711	SAMEA6657766	ERR4014824	Staphylococcus aureus	SI	Clinical_samples
One Day In Denmark	Human	2018	PRJEB37711	SAMEA6657767	ERR4014825	Staphylococcus aureus	SI	Clinical_samples
One Day In Denmark	Human	2018	PRJEB37711	SAMEA6657768	ERR4014826	Staphylococcus aureus	SI	Clinical_samples
One Day In Denmark	Human	2018	PRJEB37711	SAMEA6657769	ERR4014827	Staphylococcus aureus	SI	Clinical_samples
One Day In Denmark	Human	2018	PRJEB37711	SAMEA6657770	ERR4014828	Aerococcus urinae	SI	Clinical_samples
One Day In Denmark	Human	2018	PRJEB37711	SAMEA6657771	ERR4014829	Enterococcus faecium	SI	Clinical_samples
One Day In Denmark	Human	2018	PRJEB37711	SAMEA6657772	ERR4014830	Streptococcus agalactiae	SI	Clinical_samples
One Day In Denmark	Human	2018	PRJEB37711	SAMEA6657773	ERR4014831	Enterococcus faecalis	SI	Clinical_samples
One Day In Denmark	Human	2018	PRJEB37711	SAMEA6657774	ERR4014832	Staphylococcus epidermidis	SI	Clinical_samples
One Day In Denmark	Human	2018	PRJEB37711	SAMEA6657775	ERR4014833	Enterococcus faecium	SI	Clinical_samples
One Day In Denmark	Human	2018	PRJEB37711	SAMEA6657776	ERR4014834	Enterococcus faecium	SI	Clinical_samples
One Day In Denmark	Human	2018	PRJEB37711	SAMEA6657777	ERR4014835	Enterococcus faecalis	SI	Clinical_samples
One Day In Denmark	Human	2018	PRJEB37711	SAMEA6657778	ERR4014836	Enterococcus faecium	SI	Clinical_samples

One Day In Denmark	Human	2018	PRJEB37711	SAMEA6657779	ERR4014837	Streptococcus agalactiae	SI	Clinical_samples
One Day In Denmark	Human	2018	PRJEB37711	SAMEA6657780	ERR4014838	Enterococcus faecium	SI	Clinical_samples
One Day In Denmark	Human	2018	PRJEB37711	SAMEA6657781	ERR4014839	Enterococcus faecium	SI	Clinical_samples
One Day In Denmark	Human	2018	PRJEB37711	SAMEA6657782	ERR4014840	Schaalia turicensis	SI	Clinical_samples
One Day In Denmark	Human	2018	PRJEB37711	SAMEA6657783	ERR4014841	Staphylococcus epidermidis	SI	Clinical_samples
One Day In Denmark	Human	2018	PRJEB37711	SAMEA6657784	ERR4014842	Enterococcus faecalis	SI	Clinical_samples
One Day In Denmark	Human	2018	PRJEB37711	SAMEA6657785	ERR4014843	Enterococcus faecalis	SI	Clinical_samples
One Day In Denmark	Human	2018	PRJEB37711	SAMEA6657786	ERR4014844	Streptococcus dysgalactiae	SI	Clinical_samples
One Day In Denmark	Human	2018	PRJEB37711	SAMEA6657787	ERR4014845	Enterococcus faecalis	SI	Clinical_samples
One Day In Denmark	Human	2018	PRJEB37711	SAMEA6657788	ERR4014846	Enterococcus faecalis	SI	Clinical_samples
One Day In Denmark	Human	2018	PRJEB37711	SAMEA6657789	ERR4014847	Enterococcus faecalis	SI	Clinical_samples
One Day In Denmark	Human	2018	PRJEB37711	SAMEA6657790	ERR4014848	Streptococcus agalactiae	SI	Clinical_samples
One Day In Denmark	Human	2018	PRJEB37711	SAMEA6657791	ERR4014849	Enterococcus faecalis	SI	Clinical_samples
One Day In Denmark	Human	2018	PRJEB37711	SAMEA6657792	ERR4014850	Staphylococcus epidermidis	SI	Clinical_samples
One Day In Denmark	Human	2018	PRJEB37711	SAMEA6657793	ERR4014851	Streptococcus pneumoniae	SI	Clinical_samples
One Day In Denmark	Human	2018	PRJEB37711	SAMEA6657794	ERR4014852	Staphylococcus aureus	SI	Clinical_samples
One Day In Denmark	Human	2018	PRJEB37711	SAMEA6657795	ERR4014853	Streptococcus agalactiae	SI	Clinical_samples
One Day In Denmark	Human	2018	PRJEB37711	SAMEA6657796	ERR4014854	Streptococcus dysgalactiae	SI	Clinical_samples
One Day In Denmark	Human	2018	PRJEB37711	SAMEA6657797	ERR4014855	Streptococcus agalactiae	SI	Clinical_samples
One Day In Denmark	Human	2018	PRJEB37711	SAMEA6657798	ERR4014856	Streptococcus agalactiae	SI	Clinical_samples
One Day In Denmark	Human	2018	PRJEB37711	SAMEA6657799	ERR4014857	Streptococcus dysgalactiae	SI	Clinical_samples
One Day In Denmark	Human	2018	PRJEB37711	SAMEA6657800	ERR4014858	Streptococcus dysgalactiae	SI	Clinical_samples
One Day In Denmark	Human	2018	PRJEB37711	SAMEA6657801	ERR4014859	Staphylococcus aureus	SI	Clinical_samples
One Day In Denmark	Human	2018	PRJEB37711	SAMEA6657802	ERR4014860	Staphylococcus aureus	SI	Clinical_samples
One Day In Denmark	Human	2018	PRJEB37711	SAMEA6657803	ERR4014861	Staphylococcus aureus	SI	Clinical_samples
One Day In Denmark	Human	2018	PRJEB37711	SAMEA6657804	ERR4014862	Staphylococcus aureus	SI	Clinical_samples
One Day In Denmark	Human	2018	PRJEB37711	SAMEA6657805	ERR4014863	Streptococcus pyogenes	SI	Clinical_samples
One Day In Denmark	Human	2018	PRJEB37711	SAMEA6657806	ERR4014864	Staphylococcus aureus	SI	Clinical_samples
One Day In Denmark	Human	2018	PRJEB37711	SAMEA6657807	ERR4014865	Streptococcus pyogenes	SI	Clinical_samples
One Day In Denmark	Human	2018	PRJEB37711	SAMEA6657808	ERR4014866	Staphylococcus aureus	SI	Clinical_samples
One Day In Denmark	Human	2018	PRJEB37711	SAMEA6657809	ERR4014867	Streptococcus pyogenes	SI	Clinical_samples
One Day In Denmark	Human	2018	PRJEB37711	SAMEA6657810	ERR4014868	Staphylococcus aureus	SI	Clinical_samples
One Day In Denmark	Human	2018	PRJEB37711	SAMEA6657811	ERR4014869	Staphylococcus aureus	SI	Clinical_samples
One Day In Denmark	Human	2018	PRJEB37711	SAMEA6657812	ERR4014870	Staphylococcus saprophyticus	SI	Clinical_samples

One Day In Denmark	Human	2018	PRJEB37711	SAMEA6657813	ERR4014871	Enterococcus faecalis	SI	Clinical_samples
One Day In Denmark	Human	2018	PRJEB37711	SAMEA6657814	ERR4014872	Aerococcus urinae	SI	Clinical_samples
One Day In Denmark	Human	2018	PRJEB37711	SAMEA6657815	ERR4014873	Staphylococcus saprophyticus	SI	Clinical_samples
One Day In Denmark	Human	2018	PRJEB37711	SAMEA6657816	ERR4014874	Enterococcus faecalis	SI	Clinical_samples
One Day In Denmark	Human	2018	PRJEB37711	SAMEA6657817	ERR4014875	Staphylococcus saprophyticus	SI	Clinical_samples
One Day In Denmark	Human	2018	PRJEB37711	SAMEA6657818	ERR4014876	Streptococcus agalactiae	SI	Clinical_samples
One Day In Denmark	Human	2018	PRJEB37711	SAMEA6657819	ERR4014877	Enterococcus faecalis	SI	Clinical_samples
One Day In Denmark	Human	2018	PRJEB37711	SAMEA6657820	ERR4014878	Streptococcus agalactiae	SI	Clinical_samples
One Day In Denmark	Human	2018	PRJEB37711	SAMEA6657821	ERR4014879	Streptococcus agalactiae	SI	Clinical_samples
One Day In Denmark	Human	2018	PRJEB37711	SAMEA6657822	ERR4014880	Streptococcus pseudopneumoniae	SI	Clinical_samples
One Day In Denmark	Human	2018	PRJEB37711	SAMEA6657823	ERR4014881	Enterococcus faecalis	SI	Clinical_samples
One Day In Denmark	Human	2018	PRJEB37711	SAMEA6657824	ERR4014882	Enterococcus faecalis	SI	Clinical_samples
One Day In Denmark	Human	2018	PRJEB37711	SAMEA6657825	ERR4014883	Staphylococcus saprophyticus	SI	Clinical_samples
One Day In Denmark	Human	2018	PRJEB37711	SAMEA6657826	ERR4014884	Staphylococcus aureus	SI	Clinical_samples
One Day In Denmark	Human	2018	PRJEB37711	SAMEA6657827	ERR4014885	Staphylococcus aureus	SI	Clinical_samples
One Day In Denmark	Human	2018	PRJEB37711	SAMEA6657828	ERR4014886	Streptococcus pyogenes	SI	Clinical_samples
One Day In Denmark	Human	2018	PRJEB37711	SAMEA6657829	ERR4014887	Streptococcus pyogenes	SI	Clinical_samples
One Day In Denmark	Human	2018	PRJEB37711	SAMEA6657830	ERR4014888	Staphylococcus aureus	SI	Clinical_samples
One Day In Denmark	Human	2018	PRJEB37711	SAMEA6657831	ERR4014889	Staphylococcus aureus	SI	Clinical_samples
One Day In Denmark	Human	2018	PRJEB37711	SAMEA6657832	ERR4014890	Staphylococcus aureus	SI	Clinical_samples
One Day In Denmark	Human	2018	PRJEB37711	SAMEA6657833	ERR4014891	Streptococcus agalactiae	SI	Clinical_samples
One Day In Denmark	Human	2018	PRJEB37711	SAMEA6657834	ERR4014892	Streptococcus agalactiae	SI	Clinical_samples
One Day In Denmark	Human	2018	PRJEB37711	SAMEA6657835	ERR4014893	Streptococcus pyogenes	SI	Clinical_samples
One Day In Denmark	Human	2018	PRJEB37711	SAMEA6657836	ERR4014894	Staphylococcus aureus	SI	Clinical_samples
One Day In Denmark	Human	2018	PRJEB37711	SAMEA6657837	ERR4014895	Staphylococcus aureus	SI	Clinical_samples
One Day In Denmark	Human	2018	PRJEB37711	SAMEA6657838	ERR4014896	Staphylococcus aureus	SI	Clinical_samples
One Day In Denmark	Human	2018	PRJEB37711	SAMEA6657839	ERR4014897	Staphylococcus aureus	SI	Clinical_samples
One Day In Denmark	Human	2018	PRJEB37711	SAMEA6657840	ERR4014898	Enterococcus faecalis	SI	Clinical_samples
One Day In Denmark	Human	2018	PRJEB37711	SAMEA6657841	ERR4014899	Streptococcus pyogenes	SI	Clinical_samples
One Day In Denmark	Human	2018	PRJEB37711	SAMEA6657842	ERR4014900	Staphylococcus aureus	SI	Clinical_samples
One Day In Denmark	Human	2018	PRJEB37711	SAMEA6657843	ERR4014901	Staphylococcus aureus	SI	Clinical_samples
One Day In Denmark	Human	2018	PRJEB37711	SAMEA6657844	ERR4014902	Staphylococcus aureus	SI	Clinical_samples
One Day In Denmark	Human	2018	PRJEB37711	SAMEA6657845	ERR4014903	Staphylococcus aureus	SI	Clinical_samples
One Day In Denmark	Human	2018	PRJEB37711	SAMEA6657846	ERR4014904	Staphylococcus aureus	SI	Clinical_samples

One Day In Denmark	Human	2018	PRJEB37711	SAMEA6657847	ERR4014905	Staphylococcus aureus	SI	Clinical_samples
One Day In Denmark	Human	2018	PRJEB37711	SAMEA6657848	ERR4014906	Streptococcus dysgalactiae	SI	Clinical_samples
One Day In Denmark	Human	2018	PRJEB37711	SAMEA6657849	ERR4014907	Staphylococcus aureus	SI	Clinical_samples
One Day In Denmark	Human	2018	PRJEB37711	SAMEA6657850	ERR4014908	Streptococcus dysgalactiae	SI	Clinical_samples
One Day In Denmark	Human	2018	PRJEB37711	SAMEA6657851	ERR4014909	Staphylococcus aureus	SI	Clinical_samples
One Day In Denmark	Human	2018	PRJEB37711	SAMEA6657852	ERR4014910	Aerococcus urinae	SI	Clinical_samples
One Day In Denmark	Human	2018	PRJEB37711	SAMEA6657853	ERR4014911	Streptococcus agalactiae	SI	Clinical_samples
One Day In Denmark	Human	2018	PRJEB37711	SAMEA6657854	ERR4014912	Staphylococcus aureus	SI	Clinical_samples
One Day In Denmark	Human	2018	PRJEB37711	SAMEA6657855	ERR4014913	Enterococcus faecalis	SI	Clinical_samples
One Day In Denmark	Human	2018	PRJEB37711	SAMEA6657856	ERR4014914	Streptococcus pyogenes	SI	Clinical_samples
One Day In Denmark	Human	2018	PRJEB37711	SAMEA6657857	ERR4014915	Staphylococcus aureus	SI	Clinical_samples
One Day In Denmark	Human	2018	PRJEB37711	SAMEA6657858	ERR4014916	Streptococcus dysgalactiae	SI	Clinical_samples
One Day In Denmark	Human	2018	PRJEB37711	SAMEA6657859	ERR4014917	Streptococcus agalactiae	SI	Clinical_samples
One Day In Denmark	Human	2018	PRJEB37711	SAMEA6657860	ERR4014918	Staphylococcus aureus	SI	Clinical_samples
One Day In Denmark	Human	2018	PRJEB37711	SAMEA6657861	ERR4014919	Staphylococcus aureus	SI	Clinical_samples
One Day In Denmark	Human	2018	PRJEB37711	SAMEA6657862	ERR4014920	Staphylococcus aureus	SI	Clinical_samples
One Day In Denmark	Human	2018	PRJEB37711	SAMEA6657863	ERR4014921	Staphylococcus aureus	SI	Clinical_samples
One Day In Denmark	Human	2018	PRJEB37711	SAMEA6657864	ERR4014922	Staphylococcus aureus	SI	Clinical_samples
One Day In Denmark	Human	2018	PRJEB37711	SAMEA6657865	ERR4014923	Staphylococcus aureus	SI	Clinical_samples
One Day In Denmark	Human	2018	PRJEB37711	SAMEA6657866	ERR4014924	Staphylococcus aureus	SI	Clinical_samples
One Day In Denmark	Human	2018	PRJEB37711	SAMEA6657867	ERR4014925	Staphylococcus aureus	SI	Clinical_samples
One Day In Denmark	Human	2018	PRJEB37711	SAMEA6657868	ERR4014926	Enterococcus faecalis	SI	Clinical_samples
One Day In Denmark	Human	2018	PRJEB37711	SAMEA6657869	ERR4014927	Streptococcus agalactiae	SI	Clinical_samples
One Day In Denmark	Human	2018	PRJEB37711	SAMEA6657870	ERR4014928	Staphylococcus aureus	SI	Clinical_samples
One Day In Denmark	Human	2018	PRJEB37711	SAMEA6657871	ERR4014929	Staphylococcus aureus	SI	Clinical_samples
One Day In Denmark	Human	2018	PRJEB37711	SAMEA6657872	ERR4014930	Staphylococcus aureus	SI	Clinical_samples
One Day In Denmark	Human	2018	PRJEB37711	SAMEA6657873	ERR4014931	Staphylococcus aureus	SI	Clinical_samples
One Day In Denmark	Human	2018	PRJEB37711	SAMEA6657874	ERR4014932	Staphylococcus aureus	SI	Clinical_samples
One Day In Denmark	Human	2018	PRJEB37711	SAMEA6657875	ERR4014933	Staphylococcus aureus	SI	Clinical_samples
One Day In Denmark	Human	2018	PRJEB37711	SAMEA6657876	ERR4014934	Staphylococcus aureus	SI	Clinical_samples
One Day In Denmark	Human	2018	PRJEB37711	SAMEA6657877	ERR4014935	Streptococcus pyogenes	SI	Clinical_samples
One Day In Denmark	Human	2018	PRJEB37711	SAMEA6657878	ERR4014936	Streptococcus pyogenes	SI	Clinical_samples
One Day In Denmark	Human	2018	PRJEB37711	SAMEA6657879	ERR4014937	Staphylococcus aureus	SI	Clinical_samples
One Day In Denmark	Human	2018	PRJEB37711	SAMEA6657880	ERR4014938	Staphylococcus aureus	SI	Clinical_samples

One Day In Denmark	Human	2018	PRJEB37711	SAMEA6657881	ERR4014939	Staphylococcus aureus	SI	Clinical_samples
One Day In Denmark	Human	2018	PRJEB37711	SAMEA6657882	ERR4014940	Staphylococcus aureus	SI	Clinical_samples
One Day In Denmark	Human	2018	PRJEB37711	SAMEA6657883	ERR4014941	Streptococcus agalactiae	SI	Clinical_samples
One Day In Denmark	Human	2018	PRJEB37711	SAMEA6657884	ERR4014942	Staphylococcus aureus	SI	Clinical_samples
One Day In Denmark	Human	2018	PRJEB37711	SAMEA6657885	ERR4014943	Streptococcus pyogenes	SI	Clinical_samples
One Day In Denmark	Human	2018	PRJEB37711	SAMEA6657886	ERR4014944	Staphylococcus aureus	SI	Clinical_samples
One Day In Denmark	Human	2018	PRJEB37711	SAMEA6657887	ERR4014945	Enterococcus faecium	SI	Clinical_samples
One Day In Denmark	Human	2018	PRJEB37711	SAMEA6657888	ERR4014946	Staphylococcus aureus	SI	Clinical_samples
One Day In Denmark	Human	2018	PRJEB37711	SAMEA6657889	ERR4014947	Staphylococcus aureus	SI	Clinical_samples
One Day In Denmark	Human	2018	PRJEB37711	SAMEA6657890	ERR4014948	Staphylococcus aureus	SI	Clinical_samples
One Day In Denmark	Human	2018	PRJEB37711	SAMEA6657891	ERR4014949	Streptococcus pyogenes	SI	Clinical_samples
One Day In Denmark	Human	2018	PRJEB37711	SAMEA6657892	ERR4014950	Staphylococcus aureus	SI	Clinical_samples
One Day In Denmark	Human	2018	PRJEB37711	SAMEA6657893	ERR4014951	Staphylococcus aureus	SI	Clinical_samples
One Day In Denmark	Human	2018	PRJEB37711	SAMEA6657894	ERR4014952	Staphylococcus aureus	SI	Clinical_samples
One Day In Denmark	Human	2018	PRJEB37711	SAMEA6657895	ERR4014953	Streptococcus dysgalactiae	SI	Clinical_samples
One Day In Denmark	Human	2018	PRJEB37711	SAMEA6657896	ERR4014954	Staphylococcus aureus	SI	Clinical_samples
One Day In Denmark	Human	2018	PRJEB37711	SAMEA6657897	ERR4014955	Streptococcus agalactiae	SI	Clinical_samples
One Day In Denmark	Human	2018	PRJEB37711	SAMEA6657898	ERR4014956	Staphylococcus aureus	SI	Clinical_samples
One Day In Denmark	Human	2018	PRJEB37711	SAMEA6657899	ERR4014957	Enterococcus faecalis	SI	Clinical_samples
One Day In Denmark	Human	2018	PRJEB37711	SAMEA6657900	ERR4014958	Staphylococcus aureus	SI	Clinical_samples
One Day In Denmark	Human	2018	PRJEB37711	SAMEA6657901	ERR4014959	Streptococcus pyogenes	SI	Clinical_samples
One Day In Denmark	Human	2018	PRJEB37711	SAMEA6657902	ERR4014960	Staphylococcus aureus	SI	Clinical_samples
One Day In Denmark	Human	2018	PRJEB37711	SAMEA6657903	ERR4014961	Streptococcus agalactiae	SI	Clinical_samples
One Day In Denmark	Human	2018	PRJEB37711	SAMEA6657904	ERR4014962	Staphylococcus aureus	SI	Clinical_samples
One Day In Denmark	Human	2018	PRJEB37711	SAMEA6657905	ERR4014963	Staphylococcus aureus	SI	Clinical_samples
One Day In Denmark	Human	2018	PRJEB37711	SAMEA6657906	ERR4014964	Staphylococcus aureus	SI	Clinical_samples
One Day In Denmark	Human	2018	PRJEB37711	SAMEA6657907	ERR4014965	Staphylococcus aureus	SI	Clinical_samples
One Day In Denmark	Human	2018	PRJEB37711	SAMEA6657908	ERR4014966	Staphylococcus aureus	SI	Clinical_samples
One Day In Denmark	Human	2018	PRJEB37711	SAMEA6657909	ERR4014967	Streptococcus pyogenes	SI	Clinical_samples
One Day In Denmark	Human	2018	PRJEB37711	SAMEA6657910	ERR4014968	Staphylococcus aureus	SI	Clinical_samples
One Day In Denmark	Human	2018	PRJEB37711	SAMEA6657911	ERR4014969	Staphylococcus aureus	SI	Clinical_samples
One Day In Denmark	Human	2018	PRJEB37711	SAMEA6657912	ERR4014970	Staphylococcus aureus	SI	Clinical_samples
One Day In Denmark	Human	2018	PRJEB37711	SAMEA6657913	ERR4014971	Staphylococcus aureus	SI	Clinical_samples
One Day In Denmark	Human	2018	PRJEB37711	SAMEA6657914	ERR4014972	Enterococcus faecium	SI	Clinical_samples

One Day In Denmark	Human	2018	PRJEB37711	SAMEA6657915	ERR4014973	Staphylococcus aureus	SI	Clinical_samples
One Day In Denmark	Human	2018	PRJEB37711	SAMEA6657916	ERR4014974	Streptococcus pneumoniae	SI	Clinical_samples
One Day In Denmark	Human	2018	PRJEB37711	SAMEA6657917	ERR4014975	Staphylococcus aureus	SI	Clinical_samples
One Day In Denmark	Human	2018	PRJEB37711	SAMEA6657918	ERR4014976	Streptococcus dysgalactiae	SI	Clinical_samples
One Day In Denmark	Human	2018	PRJEB37711	SAMEA6657919	ERR4014977	Streptococcus dysgalactiae	SI	Clinical_samples
One Day In Denmark	Human	2018	PRJEB37711	SAMEA6657920	ERR4014978	Streptococcus pyogenes	SI	Clinical_samples
One Day In Denmark	Human	2018	PRJEB37711	SAMEA6657921	ERR4014979	Enterococcus faecalis	SI	Clinical_samples
One Day In Denmark	Human	2018	PRJEB37711	SAMEA6657922	ERR4014980	Enterococcus faecium	SI	Clinical_samples
One Day In Denmark	Human	2018	PRJEB37711	SAMEA6657923	ERR4014981	Enterococcus faecalis	SI	Clinical_samples
One Day In Denmark	Human	2018	PRJEB37711	SAMEA6657924	ERR4014982	Micrococcus luteus	SI	Clinical_samples
One Day In Denmark	Human	2018	PRJEB37711	SAMEA6657925	ERR4014983	Staphylococcus saprophyticus	SI	Clinical_samples
One Day In Denmark	Human	2018	PRJEB37711	SAMEA6657926	ERR4014984	Enterococcus faecium	SI	Clinical_samples
One Day In Denmark	Human	2018	PRJEB37711	SAMEA6657927	ERR4014985	Enterococcus faecium	SI	Clinical_samples
One Day In Denmark	Human	2018	PRJEB37711	SAMEA6657928	ERR4014986	Staphylococcus aureus	SI	Clinical_samples
One Day In Denmark	Human	2018	PRJEB37711	SAMEA6657929	ERR4014987	Enterococcus faecalis	SI	Clinical_samples
One Day In Denmark	Human	2018	PRJEB37711	SAMEA6657930	ERR4014988	Enterococcus faecalis	SI	Clinical_samples
One Day In Denmark	Human	2018	PRJEB37711	SAMEA6657931	ERR4014989	Enterococcus faecium	SI	Clinical_samples
One Day In Denmark	Human	2018	PRJEB37711	SAMEA6657932	ERR4014990	Streptococcus agalactiae	SI	Clinical_samples
One Day In Denmark	Human	2018	PRJEB37711	SAMEA6657933	ERR4014991	Enterococcus faecalis	SI	Clinical_samples
One Day In Denmark	Human	2018	PRJEB37711	SAMEA6657934	ERR4014992	Staphylococcus haemolyticus	SI	Clinical_samples
One Day In Denmark	Human	2018	PRJEB37711	SAMEA6657935	ERR4014993	Staphylococcus warneri	SI	Clinical_samples
One Day In Denmark	Human	2018	PRJEB37711	SAMEA6657936	ERR4014994	Staphylococcus warneri	SI	Clinical_samples
One Day In Denmark	Human	2018	PRJEB37711	SAMEA6657937	ERR4014995	Streptococcus pneumoniae	SI	Clinical_samples
One Day In Denmark	Human	2018	PRJEB37711	SAMEA6657938	ERR4014996	Staphylococcus hominis	SI	Clinical_samples
One Day In Denmark	Human	2018	PRJEB37711	SAMEA6657939	ERR4014997	Streptococcus agalactiae	SI	Clinical_samples
One Day In Denmark	Human	2018	PRJEB37711	SAMEA6657940	ERR4014998	Enterococcus faecalis	SI	Clinical_samples
One Day In Denmark	Human	2018	PRJEB37711	SAMEA6657941	ERR4014999	Staphylococcus aureus	SI	Clinical_samples
One Day In Denmark	Human	2018	PRJEB37711	SAMEA6657942	ERR4015000	Staphylococcus saprophyticus	SI	Clinical_samples
One Day In Denmark	Human	2018	PRJEB37711	SAMEA6657943	ERR4015001	Streptococcus agalactiae	SI	Clinical_samples
One Day In Denmark	Human	2018	PRJEB37711	SAMEA6657944	ERR4015002	Staphylococcus aureus	SI	Clinical_samples
One Day In Denmark	Human	2018	PRJEB37711	SAMEA6657945	ERR4015003	Streptococcus agalactiae	SI	Clinical_samples
One Day In Denmark	Human	2018	PRJEB37711	SAMEA6657946	ERR4015004	Streptococcus agalactiae	SI	Clinical_samples
One Day In Denmark	Human	2018	PRJEB37711	SAMEA6657947	ERR4015005	Streptococcus agalactiae	SI	Clinical_samples
One Day In Denmark	Human	2018	PRJEB37711	SAMEA6657948	ERR4015006	Streptococcus agalactiae	SI	Clinical_samples

One Day In Denmark	Human	2018	PRJEB37711	SAMEA6657949	ERR4015007	Streptococcus agalactiae	SI	Clinical_samples
One Day In Denmark	Human	2018	PRJEB37711	SAMEA6657950	ERR4015008	Streptococcus agalactiae	SI	Clinical_samples
One Day In Denmark	Human	2018	PRJEB37711	SAMEA6657951	ERR4015009	Streptococcus agalactiae	SI	Clinical_samples
One Day In Denmark	Human	2018	PRJEB37711	SAMEA6657952	ERR4015010	Streptococcus agalactiae	SI	Clinical_samples
One Day In Denmark	Human	2018	PRJEB37711	SAMEA6657953	ERR4015011	Staphylococcus aureus	SI	Clinical_samples
One Day In Denmark	Human	2018	PRJEB37711	SAMEA6657954	ERR4015012	Streptococcus dysgalactiae	SI	Clinical_samples
One Day In Denmark	Human	2018	PRJEB37711	SAMEA6657955	ERR4015013	Staphylococcus cohnii	SI	Clinical_samples
One Day In Denmark	Human	2018	PRJEB37711	SAMEA6657956	ERR4015014	Streptococcus dysgalactiae	SI	Clinical_samples
One Day In Denmark	Human	2018	PRJEB37711	SAMEA6657957	ERR4015015	Staphylococcus aureus	SI	Clinical_samples
One Day In Denmark	Human	2018	PRJEB37711	SAMEA6657958	ERR4015016	Streptococcus pyogenes	SI	Clinical_samples
One Day In Denmark	Human	2018	PRJEB37711	SAMEA6657959	ERR4015017	Staphylococcus aureus	SI	Clinical_samples
One Day In Denmark	Human	2018	PRJEB37711	SAMEA6657960	ERR4015018	Streptococcus dysgalactiae	SI	Clinical_samples
One Day In Denmark	Human	2018	PRJEB37711	SAMEA6657961	ERR4015019	Streptococcus dysgalactiae	SI	Clinical_samples
One Day In Denmark	Human	2018	PRJEB37711	SAMEA6657962	ERR4015020	Staphylococcus aureus	SI	Clinical_samples
One Day In Denmark	Human	2018	PRJEB37711	SAMEA6657963	ERR4015021	Streptococcus dysgalactiae	SI	Clinical_samples
One Day In Denmark	Human	2018	PRJEB37711	SAMEA6657964	ERR4015022	Haemophilus influenzae	SI	Clinical_samples
One Day In Denmark	Human	2018	PRJEB37711	SAMEA6657965	ERR4015023	Streptococcus agalactiae	SI	Clinical_samples
One Day In Denmark	Human	2018	PRJEB37711	SAMEA6657966	ERR4015024	Streptococcus dysgalactiae	SI	Clinical_samples
One Day In Denmark	Human	2018	PRJEB37711	SAMEA6657967	ERR4015025	Enterococcus faecium	SI	Clinical_samples
One Day In Denmark	Human	2018	PRJEB37711	SAMEA6657968	ERR4015026	Streptococcus agalactiae	SI	Clinical_samples
One Day In Denmark	Human	2018	PRJEB37711	SAMEA6657969	ERR4015027	Streptococcus agalactiae	SI	Clinical_samples
One Day In Denmark	Human	2018	PRJEB37711	SAMEA6657970	ERR4015028	Staphylococcus aureus	SI	Clinical_samples
One Day In Denmark	Human	2018	PRJEB37711	SAMEA6657971	ERR4015029	Streptococcus agalactiae	SI	Clinical_samples
One Day In Denmark	Human	2018	PRJEB37711	SAMEA6657972	ERR4015030	Streptococcus dysgalactiae	SI	Clinical_samples
One Day In Denmark	Human	2018	PRJEB37711	SAMEA6657973	ERR4015031	Streptococcus agalactiae	SI	Clinical_samples
One Day In Denmark	Human	2018	PRJEB37711	SAMEA6657974	ERR4015032	Streptococcus dysgalactiae	SI	Clinical_samples
One Day In Denmark	Human	2018	PRJEB37711	SAMEA6657975	ERR4015033	Staphylococcus aureus	SI	Clinical_samples
One Day In Denmark	Human	2018	PRJEB37711	SAMEA6657976	ERR4015034	Streptococcus dysgalactiae	SI	Clinical_samples
One Day In Denmark	Human	2018	PRJEB37711	SAMEA6657977	ERR4015035	Staphylococcus aureus	SI	Clinical_samples
One Day In Denmark	Human	2018	PRJEB37711	SAMEA6657978	ERR4015036	Streptococcus dysgalactiae	SI	Clinical_samples
One Day In Denmark	Human	2018	PRJEB37711	SAMEA6657979	ERR4015037	Staphylococcus aureus	SI	Clinical_samples
One Day In Denmark	Human	2018	PRJEB37711	SAMEA6657980	ERR4015038	Streptococcus dysgalactiae	SI	Clinical_samples
One Day In Denmark	Human	2018	PRJEB37711	SAMEA6657981	ERR4015039	Staphylococcus lugdunensis	SI	Clinical_samples
One Day In Denmark	Human	2018	PRJEB37711	SAMEA6657982	ERR4015040	Finegoldia magna	SI	Clinical_samples

One Day In Denmark	Human	2018	PRJEB37711	SAMEA6657983	ERR4015041	Aerococcus urinae	SI	Clinical_samples
One Day In Denmark	Human	2018	PRJEB37711	SAMEA6657984	ERR4015042	Pseudomonas aeruginosa	SI	Clinical_samples
One Day In Denmark	Human	2018	PRJEB37711	SAMEA6657985	ERR4015043	Staphylococcus aureus	SI	Clinical_samples
One Day In Denmark	Human	2018	PRJEB37711	SAMEA6657986	ERR4015044	Streptococcus agalactiae	SI	Clinical_samples
One Day In Denmark	Human	2018	PRJEB37711	SAMEA6657987	ERR4015045	Streptococcus dysgalactiae	SI	Clinical_samples
One Day In Denmark	Human	2018	PRJEB37711	SAMEA6657988	ERR4015046	Streptococcus agalactiae	SI	Clinical_samples
One Day In Denmark	Human	2018	PRJEB37711	SAMEA6657989	ERR4015047	Aerococcus sanguinicola	SI	Clinical_samples
One Day In Denmark	Human	2018	PRJEB37711	SAMEA6657990	ERR4015048	Staphylococcus aureus	SI	Clinical_samples
One Day In Denmark	Human	2018	PRJEB37711	SAMEA6657991	ERR4015049	Staphylococcus aureus	SI	Clinical_samples
One Day In Denmark	Human	2018	PRJEB37711	SAMEA6657992	ERR4015050	Streptococcus dysgalactiae	SI	Clinical_samples
One Day In Denmark	Human	2018	PRJEB37711	SAMEA6657993	ERR4015051	Streptococcus mitis	SI	Clinical_samples
One Day In Denmark	Human	2018	PRJEB37711	SAMEA6657994	ERR4015052	Staphylococcus aureus	SI	Clinical_samples
One Day In Denmark	Human	2018	PRJEB37711	SAMEA6657995	ERR4015053	Streptococcus dysgalactiae	SI	Clinical_samples
One Day In Denmark	Human	2018	PRJEB37711	SAMEA6657996	ERR4015054	Aerococcus urinae	SI	Clinical_samples
One Day In Denmark	Human	2018	PRJEB37711	SAMEA6657997	ERR4015055	Streptococcus pseudopneumoniae	SI	Clinical_samples
One Day In Denmark	Human	2018	PRJEB37711	SAMEA6657998	ERR4015056	Staphylococcus aureus	SI	Clinical_samples
One Day In Denmark	Human	2018	PRJEB37711	SAMEA6657999	ERR4015057	Streptococcus pyogenes	SI	Clinical_samples
One Day In Denmark	Human	2018	PRJEB37711	SAMEA6658000	ERR4015058	Staphylococcus aureus	SI	Clinical_samples
One Day In Denmark	Human	2018	PRJEB37711	SAMEA6658001	ERR4015059	Enterococcus faecalis	SI	Clinical_samples
One Day In Denmark	Human	2018	PRJEB37711	SAMEA6658002	ERR4015060	Staphylococcus aureus	SI	Clinical_samples
One Day In Denmark	Human	2018	PRJEB37711	SAMEA6658003	ERR4015061	Staphylococcus epidermidis	SI	Clinical_samples
One Day In Denmark	Human	2018	PRJEB37711	SAMEA6658004	ERR4015062	Staphylococcus aureus	SI	Clinical_samples
One Day In Denmark	Human	2018	PRJEB37711	SAMEA6658005	ERR4015063	Staphylococcus aureus	SI	Clinical_samples
One Day In Denmark	Human	2018	PRJEB37711	SAMEA6658006	ERR4015064	Streptococcus dysgalactiae	SI	Clinical_samples
One Day In Denmark	Human	2018	PRJEB37711	SAMEA6658007	ERR4015065	Staphylococcus aureus	SI	Clinical_samples
One Day In Denmark	Human	2018	PRJEB37711	SAMEA6658008	ERR4015066	Staphylococcus aureus	SI	Clinical_samples
One Day In Denmark	Human	2018	PRJEB37711	SAMEA6658009	ERR4015067	Staphylococcus aureus	SI	Clinical_samples
One Day In Denmark	Human	2018	PRJEB37711	SAMEA6658010	ERR4015068	Staphylococcus aureus	SI	Clinical_samples
One Day In Denmark	Human	2018	PRJEB37711	SAMEA6658011	ERR4015069	Streptococcus dysgalactiae	SI	Clinical_samples
One Day In Denmark	Human	2018	PRJEB37711	SAMEA6658012	ERR4015070	Staphylococcus epidermidis	SI	Clinical_samples
One Day In Denmark	Human	2018	PRJEB37711	SAMEA6658013	ERR4015071	Staphylococcus aureus	SI	Clinical_samples
One Day In Denmark	Human	2018	PRJEB37711	SAMEA6658014	ERR4015072	Streptococcus dysgalactiae	SI	Clinical_samples
One Day In Denmark	Human	2018	PRJEB37711	SAMEA6658015	ERR4015073	Staphylococcus aureus	SI	Clinical_samples
One Day In Denmark	Human	2018	PRJEB37711	SAMEA6658016	ERR4015074	Staphylococcus aureus	SI	Clinical_samples

One Day In Denmark	Human	2018	PRJEB37711	SAMEA6658017	ERR4015075	Staphylococcus aureus	SI	Clinical_samples
One Day In Denmark	Human	2018	PRJEB37711	SAMEA6658018	ERR4015076	Staphylococcus aureus	SI	Clinical_samples
One Day In Denmark	Human	2018	PRJEB37711	SAMEA6658019	ERR4015077	Staphylococcus aureus	SI	Clinical_samples
One Day In Denmark	Human	2018	PRJEB37711	SAMEA6658020	ERR4015078	Staphylococcus aureus	SI	Clinical_samples
One Day In Denmark	Human	2018	PRJEB37711	SAMEA6658021	ERR4015079	Staphylococcus aureus	SI	Clinical_samples
One Day In Denmark	Human	2018	PRJEB37711	SAMEA6658022	ERR4015080	Staphylococcus saprophyticus	SI	Clinical_samples
One Day In Denmark	Human	2018	PRJEB37711	SAMEA6658023	ERR4015081	Aerococcus sanguinicola	SI	Clinical_samples
One Day In Denmark	Human	2018	PRJEB37711	SAMEA6658024	ERR4015082	Staphylococcus aureus	SI	Clinical_samples
One Day In Denmark	Human	2018	PRJEB37711	SAMEA6658025	ERR4015083	Klebsiella variicola	SI	Clinical_samples
One Day In Denmark	Human	2018	PRJEB37711	SAMEA6658026	ERR4015084	Enterococcus faecalis	SI	Clinical_samples
One Day In Denmark	Human	2018	PRJEB37711	SAMEA6658027	ERR4015085	Enterococcus faecalis	SI	Clinical_samples
One Day In Denmark	Human	2018	PRJEB37711	SAMEA6658028	ERR4015086	Enterococcus faecalis	SI	Clinical_samples
One Day In Denmark	Human	2018	PRJEB37711	SAMEA6658029	ERR4015087	Streptococcus pyogenes	SI	Clinical_samples
One Day In Denmark	Human	2018	PRJEB37711	SAMEA6658030	ERR4015088	Streptococcus pyogenes	SI	Clinical_samples
One Day In Denmark	Human	2018	PRJEB37711	SAMEA6658031	ERR4015089	Staphylococcus aureus	SI	Clinical_samples
One Day In Denmark	Human	2018	PRJEB37711	SAMEA6658032	ERR4015090	Staphylococcus aureus	SI	Clinical_samples
One Day In Denmark	Human	2018	PRJEB37711	SAMEA6658033	ERR4015091	Staphylococcus aureus	SI	Clinical_samples
One Day In Denmark	Human	2018	PRJEB37711	SAMEA6658034	ERR4015092	Staphylococcus aureus	SI	Clinical_samples
One Day In Denmark	Human	2018	PRJEB37711	SAMEA6658035	ERR4015093	Staphylococcus aureus	SI	Clinical_samples
One Day In Denmark	Human	2018	PRJEB37711	SAMEA6658036	ERR4015094	Staphylococcus aureus	SI	Clinical_samples
One Day In Denmark	Human	2018	PRJEB37711	SAMEA6658037	ERR4015095	Enterococcus faecalis	SI	Clinical_samples
One Day In Denmark	Human	2018	PRJEB37711	SAMEA6658038	ERR4015096	Staphylococcus epidermidis	SI	Clinical_samples
One Day In Denmark	Human	2018	PRJEB37711	SAMEA6658039	ERR4015097	Staphylococcus hominis	SI	Clinical_samples
One Day In Denmark	Human	2018	PRJEB37711	SAMEA6658040	ERR4015098	Staphylococcus hominis	SI	Clinical_samples
One Day In Denmark	Human	2018	PRJEB37711	SAMEA6658041	ERR4015099	Staphylococcus capitis	SI	Clinical_samples
One Day In Denmark	Human	2018	PRJEB37711	SAMEA6658042	ERR4015100	Streptococcus anginosus	SI	Clinical_samples
One Day In Denmark	Human	2018	PRJEB37711	SAMEA6658043	ERR4015101	Enterococcus faecalis	SI	Clinical_samples
One Day In Denmark	Human	2018	PRJEB37711	SAMEA6658044	ERR4015102	Staphylococcus aureus	SI	Clinical_samples
One Day In Denmark	Human	2018	PRJEB37711	SAMEA6658045	ERR4015103	Staphylococcus epidermidis	SI	Clinical_samples
One Day In Denmark	Human	2018	PRJEB37711	SAMEA6658046	ERR4015104	Staphylococcus aureus	SI	Clinical_samples
One Day In Denmark	Human	2018	PRJEB37711	SAMEA6658047	ERR4015105	Staphylococcus aureus	SI	Clinical_samples
One Day In Denmark	Human	2018	PRJEB37711	SAMEA6658048	ERR4015106	Staphylococcus aureus	SI	Clinical_samples
One Day In Denmark	Human	2018	PRJEB37711	SAMEA6658049	ERR4015107	Enterococcus faecalis	SI	Clinical_samples
One Day In Denmark	Human	2018	PRJEB37711	SAMEA6658050	ERR4015108	Staphylococcus aureus	SI	Clinical_samples

One Day In Denmark	Human	2018	PRJEB37711	SAMEA6658051	ERR4015109	Enterococcus faecalis	SI	Clinical_samples
One Day In Denmark	Human	2018	PRJEB37711	SAMEA6658052	ERR4015110	Enterococcus faecalis	SI	Clinical_samples
One Day In Denmark	Human	2018	PRJEB37711	SAMEA6658053	ERR4015111	Enterococcus faecalis	SI	Clinical_samples
One Day In Denmark	Human	2018	PRJEB37711	SAMEA6658054	ERR4015112	Enterococcus faecalis	SI	Clinical_samples
One Day In Denmark	Human	2018	PRJEB37711	SAMEA6658055	ERR4015113	Enterococcus faecalis	SI	Clinical_samples
One Day In Denmark	Human	2018	PRJEB37711	SAMEA6658056	ERR4015114	Enterococcus faecalis	SI	Clinical_samples
One Day In Denmark	Human	2018	PRJEB37711	SAMEA6658057	ERR4015115	Enterococcus faecalis	SI	Clinical_samples
One Day In Denmark	Human	2018	PRJEB37711	SAMEA6658058	ERR4015116	Enterococcus faecalis	SI	Clinical_samples
One Day In Denmark	Human	2018	PRJEB37711	SAMEA6658059	ERR4015117	Enterococcus faecalis	SI	Clinical_samples
One Day In Denmark	Human	2018	PRJEB37711	SAMEA6658060	ERR4015118	Staphylococcus aureus	SI	Clinical_samples
One Day In Denmark	Human	2018	PRJEB37711	SAMEA6658061	ERR4015119	Streptococcus agalactiae	SI	Clinical_samples
One Day In Denmark	Human	2018	PRJEB37711	SAMEA6658062	ERR4015120	Enterococcus faecium	SI	Clinical_samples
One Day In Denmark	Human	2018	PRJEB37711	SAMEA6658063	ERR4015121	Streptococcus agalactiae	SI	Clinical_samples
One Day In Denmark	Human	2018	PRJEB37711	SAMEA6658064	ERR4015122	Aerococcus urinae	SI	Clinical_samples
One Day In Denmark	Human	2018	PRJEB37711	SAMEA6658065	ERR4015123	Enterococcus faecium	SI	Clinical_samples
One Day In Denmark	Human	2018	PRJEB37711	SAMEA6658066	ERR4015124	Enterococcus faecium	SI	Clinical_samples
One Day In Denmark	Human	2018	PRJEB37711	SAMEA6658067	ERR4015125	Enterococcus faecium	SI	Clinical_samples
One Day In Denmark	Human	2018	PRJEB37711	SAMEA6658068	ERR4015126	Enterococcus faecalis	SI	Clinical_samples
One Day In Denmark	Human	2018	PRJEB37711	SAMEA6658069	ERR4015127	Staphylococcus aureus	SI	Clinical_samples
One Day In Denmark	Human	2018	PRJEB37711	SAMEA6658070	ERR4015128	Staphylococcus aureus	SI	Clinical_samples
One Day In Denmark	Human	2018	PRJEB37711	SAMEA6658071	ERR4015129	Staphylococcus aureus	SI	Clinical_samples
One Day In Denmark	Human	2018	PRJEB37711	SAMEA6658072	ERR4015130	Enterococcus faecalis	SI	Clinical_samples
One Day In Denmark	Human	2018	PRJEB37711	SAMEA6658073	ERR4015131	Enterococcus faecalis	SI	Clinical_samples
One Day In Denmark	Human	2018	PRJEB37711	SAMEA6658074	ERR4015132	Staphylococcus haemolyticus	SI	Clinical_samples
One Day In Denmark	Human	2018	PRJEB37711	SAMEA6658075	ERR4015133	Enterococcus gallinarum	SI	Clinical_samples
One Day In Denmark	Human	2018	PRJEB37711	SAMEA6658076	ERR4015134	Aerococcus urinae	SI	Clinical_samples
One Day In Denmark	Human	2018	PRJEB37711	SAMEA6658077	ERR4015135	Enterococcus faecalis	SI	Clinical_samples
One Day In Denmark	Human	2018	PRJEB37711	SAMEA6658078	ERR4015136	Enterococcus faecalis	SI	Clinical_samples
One Day In Denmark	Human	2018	PRJEB37711	SAMEA6658079	ERR4015137	Enterococcus faecalis	SI	Clinical_samples
One Day In Denmark	Human	2018	PRJEB37711	SAMEA6658080	ERR4015138	Enterococcus faecalis	SI	Clinical_samples
One Day In Denmark	Human	2018	PRJEB37711	SAMEA6658081	ERR4015139	Streptococcus pyogenes	SI	Clinical_samples
One Day In Denmark	Human	2018	PRJEB37711	SAMEA6658082	ERR4015140	Staphylococcus aureus	SI	Clinical_samples
One Day In Denmark	Human	2018	PRJEB37711	SAMEA6658083	ERR4015141	Enterococcus faecium	SI	Clinical_samples
One Day In Denmark	Human	2018	PRJEB37711	SAMEA6658084	ERR4015142	Staphylococcus haemolyticus	SI	Clinical_samples

One Day In Denmark	Human	2018	PRJEB37711	SAMEA6658085	ERR4015143	Enterococcus faecalis	SI	Clinical_samples
One Day In Denmark	Human	2018	PRJEB37711	SAMEA6658086	ERR4015144	Staphylococcus aureus	SI	Clinical_samples
One Day In Denmark	Human	2018	PRJEB37711	SAMEA6658087	ERR4015145	Enterococcus faecalis	SI	Clinical_samples
One Day In Denmark	Human	2018	PRJEB37711	SAMEA6658088	ERR4015146	Enterococcus faecalis	SI	Clinical_samples
One Day In Denmark	Human	2018	PRJEB37711	SAMEA6658089	ERR4015147	Staphylococcus aureus	SI	Clinical_samples
One Day In Denmark	Human	2018	PRJEB37711	SAMEA6658090	ERR4015148	Staphylococcus aureus	SI	Clinical_samples
One Day In Denmark	Human	2018	PRJEB37711	SAMEA6658091	ERR4015149	Halomonas hydrothermalis	SI	Clinical_samples
One Day In Denmark	Human	2018	PRJEB37711	SAMEA6658092	ERR4015150	Staphylococcus aureus	SI	Clinical_samples
One Day In Denmark	Human	2018	PRJEB37711	SAMEA6658093	ERR4015151	Streptococcus pyogenes	SI	Clinical_samples
One Day In Denmark	Human	2018	PRJEB37711	SAMEA6658094	ERR4015152	Staphylococcus aureus	SI	Clinical_samples
One Day In Denmark	Human	2018	PRJEB37711	SAMEA6658095	ERR4015153	Streptococcus pyogenes	SI	Clinical_samples
One Day In Denmark	Human	2018	PRJEB37711	SAMEA6658096	ERR4015154	Streptococcus agalactiae	SI	Clinical_samples
One Day In Denmark	Human	2018	PRJEB37711	SAMEA6658097	ERR4015155	Staphylococcus aureus	SI	Clinical_samples
One Day In Denmark	Human	2018	PRJEB37711	SAMEA6658098	ERR4015156	Staphylococcus aureus	SI	Clinical_samples
One Day In Denmark	Human	2018	PRJEB37711	SAMEA6658099	ERR4015157	Staphylococcus aureus	SI	Clinical_samples
One Day In Denmark	Human	2018	PRJEB37711	SAMEA6658100	ERR4015158	Staphylococcus aureus	SI	Clinical_samples
One Day In Denmark	Human	2018	PRJEB37711	SAMEA6658101	ERR4015159	Enterococcus faecium	SI	Clinical_samples
One Day In Denmark	Human	2018	PRJEB37711	SAMEA6658102	ERR4015160	Streptococcus agalactiae	SI	Clinical_samples
One Day In Denmark	Human	2018	PRJEB37711	SAMEA6658103	ERR4015161	Staphylococcus aureus	SI	Clinical_samples
One Day In Denmark	Human	2018	PRJEB37711	SAMEA6658104	ERR4015162	Staphylococcus aureus	SI	Clinical_samples
One Day In Denmark	Human	2018	PRJEB37711	SAMEA6658105	ERR4015163	Staphylococcus aureus	SI	Clinical_samples
One Day In Denmark	Human	2018	PRJEB37711	SAMEA6658106	ERR4015164	Staphylococcus aureus	SI	Clinical_samples
One Day In Denmark	Human	2018	PRJEB37711	SAMEA6658107	ERR4015165	Staphylococcus aureus	SI	Clinical_samples
One Day In Denmark	Human	2018	PRJEB37711	SAMEA6658108	ERR4015166	Staphylococcus aureus	SI	Clinical_samples
One Day In Denmark	Human	2018	PRJEB37711	SAMEA6658109	ERR4015167	Staphylococcus aureus	SI	Clinical_samples
One Day In Denmark	Human	2018	PRJEB37711	SAMEA6658110	ERR4015168	Aerococcus urinae	SI	Clinical_samples
One Day In Denmark	Human	2018	PRJEB37711	SAMEA6658111	ERR4015169	Staphylococcus aureus	SI	Clinical_samples
One Day In Denmark	Human	2018	PRJEB37711	SAMEA6658112	ERR4015170	Streptococcus mitis	SI	Clinical_samples
One Day In Denmark	Human	2018	PRJEB37711	SAMEA6658113	ERR4015171	Staphylococcus aureus	SI	Clinical_samples
One Day In Denmark	Human	2018	PRJEB37711	SAMEA6658114	ERR4015172	Staphylococcus aureus	SI	Clinical_samples
One Day In Denmark	Human	2018	PRJEB37711	SAMEA6658115	ERR4015173	Enterococcus faecalis	SI	Clinical_samples
One Day In Denmark	Human	2018	PRJEB37711	SAMEA6658116	ERR4015174	Staphylococcus aureus	SI	Clinical_samples
One Day In Denmark	Human	2018	PRJEB37711	SAMEA6658117	ERR4015175	Staphylococcus aureus	SI	Clinical_samples
One Day In Denmark	Human	2018	PRJEB37711	SAMEA6658118	ERR4015176	Streptococcus agalactiae	SI	Clinical_samples

One Day In Denmark	Human	2018	PRJEB37711	SAMEA6658119	ERR4015177	Staphylococcus aureus	SI	Clinical_samples
One Day In Denmark	Human	2018	PRJEB37711	SAMEA6658120	ERR4015178	Staphylococcus aureus	SI	Clinical_samples
One Day In Denmark	Human	2018	PRJEB37711	SAMEA6658121	ERR4015179	Enterococcus faecalis	SI	Clinical_samples
One Day In Denmark	Human	2018	PRJEB37711	SAMEA6658122	ERR4015180	Staphylococcus aureus	SI	Clinical_samples
One Day In Denmark	Human	2018	PRJEB37711	SAMEA6658123	ERR4015181	Staphylococcus aureus	SI	Clinical_samples
One Day In Denmark	Human	2018	PRJEB37711	SAMEA6658124	ERR4015182	Staphylococcus aureus	SI	Clinical_samples
One Day In Denmark	Human	2018	PRJEB37711	SAMEA6658125	ERR4015183	Staphylococcus epidermidis	SI	Clinical_samples
One Day In Denmark	Human	2018	PRJEB37711	SAMEA6658126	ERR4015184	Staphylococcus aureus	SI	Clinical_samples
One Day In Denmark	Human	2018	PRJEB37711	SAMEA6658127	ERR4015185	Enterococcus faecium	SI	Clinical_samples
One Day In Denmark	Human	2018	PRJEB37711	SAMEA6658128	ERR4015186	Enterococcus faecium	SI	Clinical_samples
One Day In Denmark	Human	2018	PRJEB37711	SAMEA6658129	ERR4015187	Streptococcus pneumoniae	SI	Clinical_samples
One Day In Denmark	Human	2018	PRJEB37711	SAMEA6658130	ERR4015188	Streptococcus pneumoniae	SI	Clinical_samples
One Day In Denmark	Human	2018	PRJEB37711	SAMEA6658131	ERR4015189	Phocaeicola vulgatus	SI	Clinical_samples
One Day In Denmark	Human	2018	PRJEB37711	SAMEA6658132	ERR4015190	Staphylococcus aureus	SI	Clinical_samples
One Day In Denmark	Human	2018	PRJEB37711	SAMEA6658133	ERR4015191	Staphylococcus aureus	SI	Clinical_samples
One Day In Denmark	Human	2018	PRJEB37711	SAMEA6658134	ERR4015192	Staphylococcus aureus	SI	Clinical_samples
One Day In Denmark	Human	2018	PRJEB37711	SAMEA6658135	ERR4015193	Enterococcus faecalis	SI	Clinical_samples
One Day In Denmark	Human	2018	PRJEB37711	SAMEA6658136	ERR4015194	Staphylococcus aureus	SI	Clinical_samples
One Day In Denmark	Human	2018	PRJEB37711	SAMEA6658137	ERR4015195	Streptococcus pyogenes	SI	Clinical_samples
One Day In Denmark	Human	2018	PRJEB37711	SAMEA6658138	ERR4015196	Streptococcus pyogenes	SI	Clinical_samples
One Day In Denmark	Human	2018	PRJEB37711	SAMEA6658139	ERR4015197	Staphylococcus aureus	SI	Clinical_samples
One Day In Denmark	Human	2018	PRJEB37711	SAMEA6658140	ERR4015198	Streptococcus agalactiae	SI	Clinical_samples
One Day In Denmark	Human	2018	PRJEB37711	SAMEA6658141	ERR4015199	Staphylococcus aureus	SI	Clinical_samples
One Day In Denmark	Human	2018	PRJEB37711	SAMEA6658142	ERR4015200	Enterococcus faecalis	SI	Clinical_samples
One Day In Denmark	Human	2018	PRJEB37711	SAMEA6658143	ERR4015201	Enterococcus faecalis	SI	Clinical_samples
One Day In Denmark	Human	2018	PRJEB37711	SAMEA6658144	ERR4015202	Aerococcus urinae	SI	Clinical_samples
One Day In Denmark	Human	2018	PRJEB37711	SAMEA6658145	ERR4015203	Enterococcus faecalis	SI	Clinical_samples
One Day In Denmark	Human	2018	PRJEB37711	SAMEA6658146	ERR4015204	Streptococcus dysgalactiae	SI	Clinical_samples
One Day In Denmark	Human	2018	PRJEB37711	SAMEA6658147	ERR4015205	Staphylococcus aureus	SI	Clinical_samples
One Day In Denmark	Human	2018	PRJEB37711	SAMEA6658148	ERR4015206	Streptococcus dysgalactiae	SI	Clinical_samples
One Day In Denmark	Human	2018	PRJEB37711	SAMEA6658149	ERR4015207	Staphylococcus aureus	SI	Clinical_samples
One Day In Denmark	Human	2018	PRJEB37711	SAMEA6658150	ERR4015208	Aerococcus sanguinicola	SI	Clinical_samples
One Day In Denmark	Human	2018	PRJEB37711	SAMEA6658151	ERR4015209	Enterococcus faecalis	SI	Clinical_samples
One Day In Denmark	Human	2018	PRJEB37711	SAMEA6658152	ERR4015210	Staphylococcus epidermidis	SI	Clinical_samples

One Day In Denmark	Human	2018	PRJEB37711	SAMEA6658153	ERR4015211	Staphylococcus aureus	SI	Clinical_samples
One Day In Denmark	Human	2018	PRJEB37711	SAMEA6658154	ERR4015212	Staphylococcus aureus	SI	Clinical_samples
One Day In Denmark	Human	2018	PRJEB37711	SAMEA6658155	ERR4015213	Staphylococcus aureus	SI	Clinical_samples
One Day In Denmark	Human	2018	PRJEB37711	SAMEA6658156	ERR4015214	Staphylococcus aureus	SI	Clinical_samples
One Day In Denmark	Human	2018	PRJEB37711	SAMEA6658157	ERR4015215	Streptococcus dysgalactiae	SI	Clinical_samples
One Day In Denmark	Human	2018	PRJEB37711	SAMEA6658158	ERR4015216	Streptococcus dysgalactiae	SI	Clinical_samples
One Day In Denmark	Human	2018	PRJEB37711	SAMEA6658159	ERR4015217	Staphylococcus lugdunensis	SI	Clinical_samples
One Day In Denmark	Human	2018	PRJEB37711	SAMEA6658160	ERR4015218	Streptococcus pyogenes	SI	Clinical_samples
One Day In Denmark	Human	2018	PRJEB37711	SAMEA6658161	ERR4015219	Streptococcus pyogenes	SI	Clinical_samples
One Day In Denmark	Human	2018	PRJEB37711	SAMEA6658162	ERR4015220	Staphylococcus aureus	SI	Clinical_samples
One Day In Denmark	Human	2018	PRJEB37711	SAMEA6658163	ERR4015221	Staphylococcus aureus	SI	Clinical_samples
One Day In Denmark	Human	2018	PRJEB37711	SAMEA6658164	ERR4015222	Staphylococcus aureus	SI	Clinical_samples
One Day In Denmark	Human	2018	PRJEB37711	SAMEA6658165	ERR4015223	Staphylococcus aureus	SI	Clinical_samples
One Day In Denmark	Human	2018	PRJEB37711	SAMEA6658166	ERR4015224	Staphylococcus aureus	SI	Clinical_samples
One Day In Denmark	Human	2018	PRJEB37711	SAMEA6658167	ERR4015225	Staphylococcus aureus	SI	Clinical_samples
One Day In Denmark	Human	2018	PRJEB37711	SAMEA6658169	ERR4015226	Staphylococcus aureus	SI	Clinical_samples
One Day In Denmark	Human	2018	PRJEB37711	SAMEA6658170	ERR4015227	Streptococcus dysgalactiae	SI	Clinical_samples
One Day In Denmark	Human	2018	PRJEB37711	SAMEA6658171	ERR4015228	Staphylococcus aureus	SI	Clinical_samples
One Day In Denmark	Human	2018	PRJEB37711	SAMEA6658172	ERR4015229	Staphylococcus aureus	SI	Clinical_samples
One Day In Denmark	Human	2018	PRJEB37711	SAMEA6658173	ERR4015230	Staphylococcus argenteus	SI	Clinical_samples
One Day In Denmark	Human	2018	PRJEB37711	SAMEA6658174	ERR4015231	Staphylococcus aureus	SI	Clinical_samples
One Day In Denmark	Human	2018	PRJEB37711	SAMEA6658175	ERR4015232	Staphylococcus aureus	SI	Clinical_samples
One Day In Denmark	Human	2018	PRJEB37711	SAMEA6658176	ERR4015233	Staphylococcus aureus	SI	Clinical_samples
One Day In Denmark	Human	2018	PRJEB37711	SAMEA6658177	ERR4015234	Staphylococcus aureus	SI	Clinical_samples
One Day In Denmark	Human	2018	PRJEB37711	SAMEA6658178	ERR4015235	Aerococcus urinae	SI	Clinical_samples
One Day In Denmark	Human	2018	PRJEB37711	SAMEA6658179	ERR4015236	Staphylococcus aureus	SI	Clinical_samples
One Day In Denmark	Human	2018	PRJEB37711	SAMEA6658180	ERR4015237	Staphylococcus aureus	SI	Clinical_samples
One Day In Denmark	Human	2018	PRJEB37711	SAMEA6658181	ERR4015238	Staphylococcus aureus	SI	Clinical_samples
One Day In Denmark	Human	2018	PRJEB37711	SAMEA6658182	ERR4015239	Staphylococcus aureus	SI	Clinical_samples
One Day In Denmark	Human	2018	PRJEB37711	SAMEA6658183	ERR4015240	Streptococcus agalactiae	SI	Clinical_samples
One Day In Denmark	Human	2018	PRJEB37711	SAMEA6658184	ERR4015241	Staphylococcus aureus	SI	Clinical_samples
One Day In Denmark	Human	2018	PRJEB37711	SAMEA6658185	ERR4015242	Streptococcus dysgalactiae	SI	Clinical_samples
One Day In Denmark	Human	2018	PRJEB37711	SAMEA6658186	ERR4015243	Staphylococcus hominis	SI	Clinical_samples
One Day In Denmark	Human	2018	PRJEB37711	SAMEA6658187	ERR4015244	Enterococcus faecalis	SI	Clinical_samples

One Day In Denmark	Human	2018	PRJEB37711	SAMEA6658188	ERR4015245	Enterococcus faecalis	SI	Clinical_samples
One Day In Denmark	Human	2018	PRJEB37711	SAMEA6658189	ERR4015246	Staphylococcus aureus	SI	Clinical_samples
One Day In Denmark	Human	2018	PRJEB37711	SAMEA6658190	ERR4015247	Enterococcus faecalis	SI	Clinical_samples
One Day In Denmark	Human	2018	PRJEB37711	SAMEA6658191	ERR4015248	Enterococcus faecium	SI	Clinical_samples
One Day In Denmark	Human	2018	PRJEB37711	SAMEA6658192	ERR4015249	Staphylococcus aureus	SI	Clinical_samples
One Day In Denmark	Human	2018	PRJEB37711	SAMEA6658193	ERR4015250	Staphylococcus epidermidis	SI	Clinical_samples
One Day In Denmark	Human	2018	PRJEB37711	SAMEA6658194	ERR4015251	Streptococcus agalactiae	SI	Clinical_samples
One Day In Denmark	Human	2018	PRJEB37711	SAMEA6658195	ERR4015252	Staphylococcus aureus	SI	Clinical_samples
One Day In Denmark	Human	2018	PRJEB37711	SAMEA6658196	ERR4015253	Staphylococcus aureus	SI	Clinical_samples
One Day In Denmark	Human	2018	PRJEB37711	SAMEA6658197	ERR4015254	Staphylococcus aureus	SI	Clinical_samples
One Day In Denmark	Human	2018	PRJEB37711	SAMEA6658198	ERR4015255	Staphylococcus aureus	SI	Clinical_samples
One Day In Denmark	Human	2018	PRJEB37711	SAMEA6658199	ERR4015256	Streptococcus agalactiae	SI	Clinical_samples
One Day In Denmark	Human	2018	PRJEB37711	SAMEA6658200	ERR4015257	Staphylococcus aureus	SI	Clinical_samples
One Day In Denmark	Human	2018	PRJEB37711	SAMEA6658201	ERR4015258	Staphylococcus aureus	SI	Clinical_samples
One Day In Denmark	Human	2018	PRJEB37711	SAMEA6658202	ERR4015259	Staphylococcus aureus	SI	Clinical_samples
One Day In Denmark	Human	2018	PRJEB37711	SAMEA6658203	ERR4015260	Staphylococcus epidermidis	SI	Clinical_samples
One Day In Denmark	Human	2018	PRJEB37711	SAMEA6658204	ERR4015261	Enterococcus faecium	SI	Clinical_samples
One Day In Denmark	Human	2018	PRJEB37711	SAMEA6658205	ERR4015262	Staphylococcus aureus	SI	Clinical_samples
One Day In Denmark	Human	2018	PRJEB37711	SAMEA6658206	ERR4015263	Staphylococcus epidermidis	SI	Clinical_samples
One Day In Denmark	Human	2018	PRJEB37711	SAMEA6658207	ERR4015264	Staphylococcus epidermidis	SI	Clinical_samples
One Day In Denmark	Human	2018	PRJEB37711	SAMEA6658208	ERR4015265	Streptococcus pyogenes	SI	Clinical_samples
One Day In Denmark	Human	2018	PRJEB37711	SAMEA6658209	ERR4015266	Enterococcus faecium	SI	Clinical_samples
One Day In Denmark	Human	2018	PRJEB37711	SAMEA6658210	ERR4015267	Haemophilus influenzae	SI	Clinical_samples
One Day In Denmark	Human	2018	PRJEB37711	SAMEA6658211	ERR4015268	Staphylococcus epidermidis	SI	Clinical_samples
One Day In Denmark	Human	2018	PRJEB37711	SAMEA6658212	ERR4015269	Cutibacterium acnes	SI	Clinical_samples
One Day In Denmark	Human	2018	PRJEB37711	SAMEA6658213	ERR4015270	Cutibacterium acnes	SI	Clinical_samples
One Day In Denmark	Human	2018	PRJEB37711	SAMEA6658214	ERR4015271	Cutibacterium acnes	SI	Clinical_samples
One Day In Denmark	Human	2018	PRJEB37711	SAMEA6658215	ERR4015272	Cutibacterium avidum	SI	Clinical_samples
One Day In Denmark	Human	2018	PRJEB37711	SAMEA6658216	ERR4015273	Cutibacterium acnes	SI	Clinical_samples
One Day In Denmark	Human	2018	PRJEB37711	SAMEA6658217	ERR4015274	Staphylococcus aureus	SI	Clinical_samples
One Day In Denmark	Human	2018	PRJEB37711	SAMEA6658218	ERR4015275	Staphylococcus epidermidis	SI	Clinical_samples
One Day In Denmark	Human	2018	PRJEB37711	SAMEA6658219	ERR4015276	Staphylococcus epidermidis	SI	Clinical_samples
One Day In Denmark	Human	2018	PRJEB37711	SAMEA6658220	ERR4015277	Staphylococcus epidermidis	SI	Clinical_samples
One Day In Denmark	Human	2018	PRJEB37711	SAMEA6658221	ERR4015278	Staphylococcus epidermidis	SI	Clinical_samples

One Day In Denmark	Human	2018	PRJEB37711	SAMEA6658222	ERR4015279	Enterococcus casseliflavus	SI	Clinical_samples
One Day In Denmark	Human	2018	PRJEB37711	SAMEA6658223	ERR4015280	Staphylococcus epidermidis	SI	Clinical_samples
One Day In Denmark	Human	2018	PRJEB37711	SAMEA6658224	ERR4015281	Streptococcus oralis	SI	Clinical_samples
One Day In Denmark	Human	2018	PRJEB37711	SAMEA6658225	ERR4015282	Staphylococcus aureus	SI	Clinical_samples
One Day In Denmark	Human	2018	PRJEB37711	SAMEA6658226	ERR4015283	Staphylococcus aureus	SI	Clinical_samples
One Day In Denmark	Human	2018	PRJEB37711	SAMEA6658227	ERR4015284	Staphylococcus aureus	SI	Clinical_samples
One Day In Denmark	Human	2018	PRJEB37711	SAMEA6658228	ERR4015285	Streptococcus pneumoniae	SI	Clinical_samples
One Day In Denmark	Human	2018	PRJEB37711	SAMEA6658229	ERR4015286	Staphylococcus aureus	SI	Clinical_samples
One Day In Denmark	Human	2018	PRJEB37711	SAMEA6658230	ERR4015287	Staphylococcus hominis	SI	Clinical_samples
One Day In Denmark	Human	2018	PRJEB37711	SAMEA6658231	ERR4015288	Streptococcus pneumoniae	SI	Clinical_samples
One Day In Denmark	Human	2018	PRJEB37711	SAMEA6658232	ERR4015289	Enterococcus faecalis	SI	Clinical_samples
One Day In Denmark	Human	2018	PRJEB37711	SAMEA6658233	ERR4015290	Streptococcus dysgalactiae	SI	Clinical_samples
One Day In Denmark	Human	2018	PRJEB37711	SAMEA6658234	ERR4015291	Streptococcus agalactiae	SI	Clinical_samples
One Day In Denmark	Human	2018	PRJEB37711	SAMEA6658235	ERR4015292	Enterococcus faecium	SI	Clinical_samples
One Day In Denmark	Human	2018	PRJEB37711	SAMEA6658236	ERR4015293	Enterococcus faecalis	SI	Clinical_samples
One Day In Denmark	Human	2018	PRJEB37711	SAMEA6658237	ERR4015294	Streptococcus pneumoniae	SI	Clinical_samples
One Day In Denmark	Human	2018	PRJEB37711	SAMEA6658238	ERR4015295	Streptococcus pneumoniae	SI	Clinical_samples
One Day In Denmark	Human	2018	PRJEB37711	SAMEA6658239	ERR4015296	Staphylococcus aureus	SI	Clinical_samples
One Day In Denmark	Human	2018	PRJEB37711	SAMEA6658240	ERR4015297	Staphylococcus aureus	SI	Clinical_samples
One Day In Denmark	Human	2018	PRJEB37711	SAMEA6658241	ERR4015298	Staphylococcus aureus	SI	Clinical_samples
One Day In Denmark	Human	2018	PRJEB37711	SAMEA6658242	ERR4015299	Streptococcus agalactiae	SI	Clinical_samples
One Day In Denmark	Human	2018	PRJEB37711	SAMEA6658243	ERR4015300	Enterococcus faecalis	SI	Clinical_samples
One Day In Denmark	Human	2018	PRJEB37711	SAMEA6658244	ERR4015301	Aerococcus urinae	SI	Clinical_samples
One Day In Denmark	Human	2018	PRJEB37711	SAMEA6658245	ERR4015302	Staphylococcus aureus	SI	Clinical_samples
One Day In Denmark	Human	2018	PRJEB37711	SAMEA6658246	ERR4015303	Staphylococcus aureus	SI	Clinical_samples
One Day In Denmark	Human	2018	PRJEB37711	SAMEA6658247	ERR4015304	Streptococcus dysgalactiae	SI	Clinical_samples
One Day In Denmark	Human	2018	PRJEB37711	SAMEA6658248	ERR4015305	Staphylococcus aureus	SI	Clinical_samples
One Day In Denmark	Human	2018	PRJEB37711	SAMEA6658249	ERR4015306	Streptococcus agalactiae	SI	Clinical_samples
One Day In Denmark	Human	2018	PRJEB37711	SAMEA6658250	ERR4015307	Enterococcus faecalis	SI	Clinical_samples
One Day In Denmark	Human	2018	PRJEB37711	SAMEA6658251	ERR4015308	Enterococcus faecalis	SI	Clinical_samples
One Day In Denmark	Human	2018	PRJEB37711	SAMEA6658252	ERR4015309	Enterococcus faecalis	SI	Clinical_samples
One Day In Denmark	Human	2018	PRJEB37711	SAMEA6658253	ERR4015310	Staphylococcus aureus	SI	Clinical_samples
One Day In Denmark	Human	2018	PRJEB37711	SAMEA6658254	ERR4015311	Staphylococcus aureus	SI	Clinical_samples
One Day In Denmark	Human	2018	PRJEB37711	SAMEA6658255	ERR4015312	Staphylococcus aureus	SI	Clinical_samples

One Day In Denmark	Human	2018	PRJEB37711	SAMEA6658256	ERR4015313	Staphylococcus lugdunensis	SI	Clinical_samples
One Day In Denmark	Human	2018	PRJEB37711	SAMEA6658257	ERR4015314	Staphylococcus aureus	SI	Clinical_samples
One Day In Denmark	Human	2018	PRJEB37711	SAMEA6658258	ERR4015315	Staphylococcus aureus	SI	Clinical_samples
One Day In Denmark	Human	2018	PRJEB37711	SAMEA6658259	ERR4015316	Staphylococcus aureus	SI	Clinical_samples
One Day In Denmark	Human	2018	PRJEB37711	SAMEA6658260	ERR4015317	Streptococcus pneumoniae	SI	Clinical_samples
One Day In Denmark	Human	2018	PRJEB37711	SAMEA6658261	ERR4015318	Staphylococcus saprophyticus	SI	Clinical_samples
One Day In Denmark	Human	2018	PRJEB37711	SAMEA6658262	ERR4015319	Pseudomonas aeruginosa	SI	Clinical_samples
One Day In Denmark	Human	2018	PRJEB37711	SAMEA6658263	ERR4015320	Staphylococcus aureus	SI	Clinical_samples
One Day In Denmark	Human	2018	PRJEB37711	SAMEA6658264	ERR4015321	Staphylococcus aureus	SI	Clinical_samples
One Day In Denmark	Human	2018	PRJEB37711	SAMEA6658265	ERR4015322	Staphylococcus aureus	SI	Clinical_samples
One Day In Denmark	Human	2018	PRJEB37711	SAMEA6658266	ERR4015323	Staphylococcus lugdunensis	SI	Clinical_samples
One Day In Denmark	Human	2018	PRJEB37711	SAMEA6658267	ERR4015324	Streptococcus dysgalactiae	SI	Clinical_samples
One Day In Denmark	Human	2018	PRJEB37711	SAMEA6658268	ERR4015325	Streptococcus pneumoniae	SI	Clinical_samples
One Day In Denmark	Human	2018	PRJEB37711	SAMEA6658269	ERR4015326	Staphylococcus aureus	SI	Clinical_samples
One Day In Denmark	Human	2018	PRJEB37711	SAMEA6658270	ERR4015327	Staphylococcus aureus	SI	Clinical_samples
One Day In Denmark	Human	2018	PRJEB37711	SAMEA6658271	ERR4015328	Staphylococcus aureus	SI	Clinical_samples
One Day In Denmark	Human	2018	PRJEB37711	SAMEA6658272	ERR4015329	Staphylococcus aureus	SI	Clinical_samples
One Day In Denmark	Human	2018	PRJEB37711	SAMEA6658273	ERR4015330	Staphylococcus aureus	SI	Clinical_samples
One Day In Denmark	Human	2018	PRJEB37711	SAMEA6658274	ERR4015331	Corynebacterium striatum	SI	Clinical_samples
One Day In Denmark	Human	2018	PRJEB37711	SAMEA6658275	ERR4015332	Streptococcus pyogenes	SI	Clinical_samples
One Day In Denmark	Human	2018	PRJEB37711	SAMEA6658276	ERR4015333	Staphylococcus aureus	SI	Clinical_samples
One Day In Denmark	Human	2018	PRJEB37711	SAMEA6658277	ERR4015334	Staphylococcus aureus	SI	Clinical_samples
One Day In Denmark	Human	2018	PRJEB37711	SAMEA6658278	ERR4015335	Staphylococcus aureus	SI	Clinical_samples
One Day In Denmark	Human	2018	PRJEB37711	SAMEA6658279	ERR4015336	Staphylococcus aureus	SI	Clinical_samples
One Day In Denmark	Human	2018	PRJEB37711	SAMEA6658280	ERR4015337	Staphylococcus aureus	SI	Clinical_samples
One Day In Denmark	Human	2018	PRJEB37711	SAMEA6658281	ERR4015338	Streptococcus pyogenes	SI	Clinical_samples
One Day In Denmark	Human	2018	PRJEB37711	SAMEA6658282	ERR4015339	Staphylococcus aureus	SI	Clinical_samples
One Day In Denmark	Human	2018	PRJEB37711	SAMEA6658283	ERR4015340	Streptococcus dysgalactiae	SI	Clinical_samples
One Day In Denmark	Human	2018	PRJEB37711	SAMEA6658284	ERR4015341	Streptococcus agalactiae	SI	Clinical_samples
One Day In Denmark	Human	2018	PRJEB37711	SAMEA6658285	ERR4015342	Staphylococcus aureus	SI	Clinical_samples
One Day In Denmark	Human	2018	PRJEB37711	SAMEA6658286	ERR4015343	Streptococcus pneumoniae	SI	Clinical_samples
One Day In Denmark	Human	2018	PRJEB37711	SAMEA6658287	ERR4015344	Streptococcus pneumoniae	SI	Clinical_samples
One Day In Denmark	Human	2018	PRJEB37711	SAMEA6658288	ERR4015345	Streptococcus pneumoniae	SI	Clinical_samples
One Day In Denmark	Human	2018	PRJEB37711	SAMEA6658289	ERR4015346	Streptococcus pneumoniae	SI	Clinical_samples

One Day In Denmark	Human	2018	PRJEB37711	SAMEA6658290	ERR4015347	Streptococcus pneumoniae	SI	Clinical_samples
One Day In Denmark	Human	2018	PRJEB37711	SAMEA6658291	ERR4015348	Streptococcus pneumoniae	SI	Clinical_samples
One Day In Denmark	Human	2018	PRJEB37711	SAMEA6806105	ERR4076735	Klebsiella sp. 1D-196	SI	Clinical_samples
One Day In Denmark	Human	2018	PRJEB37711	SAMEA6806106	ERR4076736	Streptococcus sp. 1D-394	SI	Clinical_samples
One Day In Denmark	Human	2018	PRJEB37711	SAMEA6806107	ERR4076737	Klebsiella sp. 1D-203	SI	Clinical_samples
One Day In Denmark	Human	2018	PRJEB37711	SAMEA6806108	ERR4076738	Corynebacterium sp. 1D-458	SI	Clinical_samples
One Day In Denmark	Human	2018	PRJEB37711	SAMEA6806109	ERR4076739	Aerococcus sp. 1D-463	SI	Clinical_samples
One Day In Denmark	Human	2018	PRJEB37711	SAMEA6806110	ERR4076740	Aerococcus sp. 1D-488	SI	Clinical_samples
One Day In Denmark	Human	2018	PRJEB37711	SAMEA6806111	ERR4076741	Pasteurella sp. 1D-532	SI	Clinical_samples
One Day In Denmark	Human	2018	PRJEB37711	SAMEA6806112	ERR4076742	Klebsiella sp. 1D-558	SI	Clinical_samples
One Day In Denmark	Human	2018	PRJEB37711	SAMEA6806113	ERR4076743	Enterobacter sp. 1D-573	SI	Clinical_samples
One Day In Denmark	Human	2018	PRJEB37711	SAMEA6806114	ERR4076744	Enterobacter sp. 1D-589	SI	Clinical_samples
One Day In Denmark	Human	2018	PRJEB37711	SAMEA6806115	ERR4076745	Enterobacter sp. 1D-616	SI	Clinical_samples
One Day In Denmark	Human	2018	PRJEB37711	SAMEA6806116	ERR4076746	Enterobacter sp. 1D-649	SI	Clinical_samples
One Day In Denmark	Human	2018	PRJEB37711	SAMEA6806117	ERR4076747	Klebsiella sp. 1D-673	SI	Clinical_samples
One Day In Denmark	Human	2018	PRJEB37711	SAMEA6806118	ERR4076748	Klebsiella sp. 1D-933	SI	Clinical_samples
One Day In Denmark	Human	2018	PRJEB37711	SAMEA6806119	ERR4076749	Pseudomonas sp. 1D-967	SI	Clinical_samples
One Day In Denmark	Human	2018	PRJEB37711	SAMEA6806120	ERR4076750	Klebsiella sp. 1D-976	SI	Clinical_samples
One Day In Denmark	Human	2018	PRJEB37711	SAMEA6806121	ERR4076751	Klebsiella sp. 1D-995	SI	Clinical_samples
One Day In Denmark	Human	2018	PRJEB37711	SAMEA6806122	ERR4076752	Klebsiella sp. 1D-1084	SI	Clinical_samples
One Day In Denmark	Human	2018	PRJEB37711	SAMEA6806123	ERR4076753	Enterobacter sp. 1D-1140	SI	Clinical_samples
One Day In Denmark	Human	2018	PRJEB37711	SAMEA6806124	ERR4076754	Klebsiella sp. 1D-1159	SI	Clinical_samples
One Day In Denmark	Human	2018	PRJEB37711	SAMEA6806125	ERR4076755	Enterobacter sp. 1D-1170	SI	Clinical_samples
One Day In Denmark	Human	2018	PRJEB37711	SAMEA6806126	ERR4076756	Enterobacter sp. 1D-1220	SI	Clinical_samples
One Day In Denmark	Human	2018	PRJEB37711	SAMEA6806127	ERR4076757	Klebsiella sp. 1D-1264	SI	Clinical_samples
One Day In Denmark	Human	2018	PRJEB37711	SAMEA6806128	ERR4076758	Klebsiella sp. 1D-1275	SI	Clinical_samples
One Day In Denmark	Human	2018	PRJEB37711	SAMEA6806129	ERR4076759	Enterobacter sp. 1D-1306	SI	Clinical_samples
One Day In Denmark	Human	2018	PRJEB37711	SAMEA6806130	ERR4076760	Klebsiella sp. 1D-1399	SI	Clinical_samples
One Day In Denmark	Human	2018	PRJEB37711	SAMEA6806131	ERR4076761	Anaerococcus sp. 1D-1404	SI	Clinical_samples
One Day In Denmark	Human	2018	PRJEB37711	SAMEA6806132	ERR4076762	Propionibacterium sp. 1D-1660	SI	Clinical_samples
One Day In Denmark	Human	2018	PRJEB37711	SAMEA6806133	ERR4076763	Aerococcus sp. 1D-1724	SI	Clinical_samples
One Day In Denmark	Human	2018	PRJEB37711	SAMEA6806134	ERR4076764	Streptococcus sp. 1D-1870	SI	Clinical_samples
One Day In Denmark	Human	2018	PRJEB37711	SAMEA6806135	ERR4076765	Corynebacterium sp. 1D-1933	SI	Clinical_samples
One Day In Denmark	Human	2018	PRJEB37711	SAMEA6806136	ERR4076766	Corynebacterium sp. 1D-1935	SI	Clinical_samples

One Day In Denmark	Human	2018	PRJEB37711	SAMEA6806137	ERR4076767	Streptococcus sp. 1D-1961	SI	Clinical_samples
One Day In Denmark	Human	2018	PRJEB37711	SAMEA6806138	ERR4076768	Actinotignum sp. 1D-1971	SI	Clinical_samples
One Day In Denmark	Human	2018	PRJEB37711	SAMEA6806139	ERR4076769	Klebsiella sp. 1D-182	SI	Clinical_samples
One Day In Denmark	Human	2018	PRJEB37711	SAMEA6806140	ERR4076770	Bacterium 1D-500	SI	Clinical_samples
One Day In Denmark	Human	2018	PRJEB37711	SAMEA6806141	ERR4076771	Bacterium 1D-1110	SI	Clinical_samples
One Day In Denmark	Human	2018	PRJEB37711	SAMEA6819210	ERR4129095	Escherichia coli	SI	Clinical_samples
Danmap	Pig	2017	PRJEB50613	SAMEA12813959	ERR8314729	pig gut metagenome	MG	Faecal_samples
Danmap	Pig	2017	PRJEB50613	SAMEA12813960	ERR8314730	pig gut metagenome	MG	Faecal_samples
Danmap	Pig	2017	PRJEB50613	SAMEA12813961	ERR8314731	pig gut metagenome	MG	Faecal_samples
Danmap	Pig	2017	PRJEB50613	SAMEA12813962	ERR8314732	pig gut metagenome	MG	Faecal_samples
Danmap	Pig	2018	PRJEB50613	SAMEA12813963	ERR8314733	pig gut metagenome	MG	Faecal_samples
Danmap	Pig	2018	PRJEB50613	SAMEA12813964	ERR8314734	pig gut metagenome	MG	Faecal_samples
Danmap	Pig	2018	PRJEB50613	SAMEA12813965	ERR8314735	pig gut metagenome	MG	Faecal_samples
Danmap	Pig	2018	PRJEB50613	SAMEA12813966	ERR8314736	pig gut metagenome	MG	Faecal_samples
Danmap	Pig	2018	PRJEB50613	SAMEA12813967	ERR8314737	pig gut metagenome	MG	Faecal_samples
Danmap	Pig	2018	PRJEB50613	SAMEA12813969	ERR8314739	pig gut metagenome	MG	Faecal_samples
Danmap	Pig	2018	PRJEB50613	SAMEA12813970	ERR8314740	pig gut metagenome	MG	Faecal_samples
Danmap	Pig	2018	PRJEB50613	SAMEA12813971	ERR8314741	pig gut metagenome	MG	Faecal_samples
Danmap	Pig	2018	PRJEB50613	SAMEA12813972	ERR8314742	pig gut metagenome	MG	Faecal_samples
Danmap	Pig	2018	PRJEB50613	SAMEA12813973	ERR8314743	pig gut metagenome	MG	Faecal_samples
Danmap	Pig	2018	PRJEB50613	SAMEA12813974	ERR8314744	pig gut metagenome	MG	Faecal_samples
Danmap	Pig	2018	PRJEB50613	SAMEA12813975	ERR8314745	pig gut metagenome	MG	Faecal_samples
Danmap	Pig	2015	PRJEB50613	SAMEA12813992	ERR8314762	pig gut metagenome	MG	Faecal_samples
Danmap	Pig	2015	PRJEB50613	SAMEA12813993	ERR8314763	pig gut metagenome	MG	Faecal_samples
Danmap	Pig	2015	PRJEB50613	SAMEA12813994	ERR8314764	pig gut metagenome	MG	Faecal_samples
Danmap	Pig	2015	PRJEB50613	SAMEA12813995	ERR8314765	pig gut metagenome	MG	Faecal_samples
Danmap	Pig	2015	PRJEB50613	SAMEA12813996	ERR8314766	pig gut metagenome	MG	Faecal_samples
Danmap	Pig	2015	PRJEB50613	SAMEA12813997	ERR8314767	pig gut metagenome	MG	Faecal_samples
Danmap	Pig	2015	PRJEB50613	SAMEA12813998	ERR8314768	pig gut metagenome	MG	Faecal_samples
Danmap	Pig	2015	PRJEB50613	SAMEA12813999	ERR8314769	pig gut metagenome	MG	Faecal_samples
Danmap	Pig	2015	PRJEB50613	SAMEA12814001	ERR8314771	pig gut metagenome	MG	Faecal_samples
Danmap	Pig	2016	PRJEB50613	SAMEA12814002	ERR8314772	pig gut metagenome	MG	Faecal_samples
Danmap	Pig	2016	PRJEB50613	SAMEA12814003	ERR8314773	pig gut metagenome	MG	Faecal_samples
Danmap	Pig	2016	PRJEB50613	SAMEA12814004	ERR8314774	pig gut metagenome	MG	Faecal_samples

Danmap	Pig	2016	PRJEB50613	SAMEA12814005	ERR8314775	pig gut metagenome	MG	Faecal_samples
Danmap	Pig	2016	PRJEB50613	SAMEA12814006	ERR8314776	pig gut metagenome	MG	Faecal_samples
Danmap	Pig	2016	PRJEB50613	SAMEA12814007	ERR8314777	pig gut metagenome	MG	Faecal_samples
Danmap	Pig	2016	PRJEB50613	SAMEA12814008	ERR8314778	pig gut metagenome	MG	Faecal_samples
Danmap	Pig	2016	PRJEB50613	SAMEA12814009	ERR8314779	pig gut metagenome	MG	Faecal_samples
Danmap	Pig	2016	PRJEB50613	SAMEA12814011	ERR8314781	pig gut metagenome	MG	Faecal_samples
Danmap	Pig	2017	PRJEB50613	SAMEA12814012	ERR8314782	pig gut metagenome	MG	Faecal_samples
Danmap	Pig	2017	PRJEB50613	SAMEA12814013	ERR8314783	pig gut metagenome	MG	Faecal_samples
Danmap	Pig	2017	PRJEB50613	SAMEA12814014	ERR8314784	pig gut metagenome	MG	Faecal_samples
Danmap	Pig	2017	PRJEB50613	SAMEA12814015	ERR8314785	pig gut metagenome	MG	Faecal_samples
Danmap	Pig	2017	PRJEB50613	SAMEA12814016	ERR8314786	pig gut metagenome	MG	Faecal_samples
Danmap	Pig	2017	PRJEB50613	SAMEA12814017	ERR8314787	pig gut metagenome	MG	Faecal_samples
Danmap	Pig	2017	PRJEB50613	SAMEA12814018	ERR8314788	pig gut metagenome	MG	Faecal_samples
Danmap	Pig	2017	PRJEB50613	SAMEA12814019	ERR8314789	pig gut metagenome	MG	Faecal_samples
Danish VETII project	Pig	2014	PRJEB26961	SAMEA4688837	ERR2597323	gut metagenome	MG	Faecal_samples
Danish VETII project	Pig	2015	PRJEB26961	SAMEA4688838	ERR2597324	gut metagenome	MG	Faecal_samples
Danish VETII project	Pig	2014	PRJEB26961	SAMEA4688839	ERR2597325	gut metagenome	MG	Faecal_samples
Danish VETII project	Pig	2015	PRJEB26961	SAMEA4688840	ERR2597326	gut metagenome	MG	Faecal_samples
Danish VETII project	Pig	2015	PRJEB26961	SAMEA4688841	ERR2597327	gut metagenome	MG	Faecal_samples
Danish VETII project	Pig	2015	PRJEB26961	SAMEA4688842	ERR2597328	gut metagenome	MG	Faecal_samples
Danish VETII project	Pig	2015	PRJEB26961	SAMEA4688843	ERR2597329	gut metagenome	MG	Faecal_samples
Danish VETII project	Pig	2015	PRJEB26961	SAMEA4688844	ERR2597330	gut metagenome	MG	Faecal_samples
Danish VETII project	Pig	2015	PRJEB26961	SAMEA4688845	ERR2597331	gut metagenome	MG	Faecal_samples
Danish VETII project	Pig	2015	PRJEB26961	SAMEA4688846	ERR2597332	gut metagenome	MG	Faecal_samples
Danish VETII project	Pig	2015	PRJEB26961	SAMEA4688847	ERR2597333	gut metagenome	MG	Faecal_samples
Danish VETII project	Pig	2015	PRJEB26961	SAMEA4688848	ERR2597334	gut metagenome	MG	Faecal_samples
Danish VETII project	Pig	2014	PRJEB26961	SAMEA4688849	ERR2597335	gut metagenome	MG	Faecal_samples
Danish VETII project	Pig	2015	PRJEB26961	SAMEA4688850	ERR2597336	gut metagenome	MG	Faecal_samples
Danish VETII project	Pig	2015	PRJEB26961	SAMEA4688851	ERR2597337	gut metagenome	MG	Faecal_samples
Danish VETII project	Pig	2015	PRJEB26961	SAMEA4688852	ERR2597338	gut metagenome	MG	Faecal_samples
Danish VETII project	Pig	2015	PRJEB26961	SAMEA4688853	ERR2597339	gut metagenome	MG	Faecal_samples
Danish VETII project	Pig	2015	PRJEB26961	SAMEA4688854	ERR2597340	gut metagenome	MG	Faecal_samples
Danish VETII project	Pig	2015	PRJEB26961	SAMEA4688855	ERR2597341	gut metagenome	MG	Faecal_samples
Danish VETII project	Pig	2015	PRJEB26961	SAMEA4688856	ERR2597342	gut metagenome	MG	Faecal_samples

Danish VETII project	Pig	2015	PRJEB26961	SAMEA4688857	ERR2597343	gut metagenome	MG	Faecal_samples
Danish VETII project	Pig	2015	PRJEB26961	SAMEA4688858	ERR2597344	gut metagenome	MG	Faecal_samples
Danish VETII project	Pig	2015	PRJEB26961	SAMEA4688859	ERR2597345	gut metagenome	MG	Faecal_samples
Danish VETII project	Pig	2014	PRJEB26961	SAMEA4688860	ERR2597346	gut metagenome	MG	Faecal_samples
Danish VETII project	Pig	2014	PRJEB26961	SAMEA4688861	ERR2597347	gut metagenome	MG	Faecal_samples
Danish VETII project	Pig	2014	PRJEB26961	SAMEA4688862	ERR2597348	gut metagenome	MG	Faecal_samples
Danish VETII project	Pig	2014	PRJEB26961	SAMEA4688863	ERR2597349	gut metagenome	MG	Faecal_samples
Danish VETII project	Pig	2014	PRJEB26961	SAMEA4688864	ERR2597350	gut metagenome	MG	Faecal_samples
Danish VETII project	Pig	2014	PRJEB26961	SAMEA4688865	ERR2597351	gut metagenome	MG	Faecal_samples
Danish VETII project	Pig	2014	PRJEB26961	SAMEA4688866	ERR2597352	gut metagenome	MG	Faecal_samples
Danish VETII project	Pig	2014	PRJEB26961	SAMEA4688867	ERR2597353	gut metagenome	MG	Faecal_samples
Danish VETII project	Pig	2014	PRJEB26961	SAMEA4688868	ERR2597354	gut metagenome	MG	Faecal_samples
Danish VETII project	Pig	2014	PRJEB26961	SAMEA4688869	ERR2597355	gut metagenome	MG	Faecal_samples
Danish VETII project	Pig	2015	PRJEB26961	SAMEA4688870	ERR2597356	gut metagenome	MG	Faecal_samples
Danish VETII project	Pig	2016	PRJEB26961	SAMEA4688871	ERR2597357	gut metagenome	MG	Faecal_samples
Danish VETII project	Pig	2016	PRJEB26961	SAMEA4688872	ERR2597358	gut metagenome	MG	Faecal_samples
Danish VETII project	Pig	2016	PRJEB26961	SAMEA4688873	ERR2597359	gut metagenome	MG	Faecal_samples
Danish VETII project	Pig	2016	PRJEB26961	SAMEA4688874	ERR2597360	gut metagenome	MG	Faecal_samples
Danish VETII project	Pig	2016	PRJEB26961	SAMEA4688875	ERR2597361	gut metagenome	MG	Faecal_samples
Danish VETII project	Pig	2016	PRJEB26961	SAMEA4688876	ERR2597362	gut metagenome	MG	Faecal_samples
Danish VETII project	Pig	2016	PRJEB26961	SAMEA4688877	ERR2597363	gut metagenome	MG	Faecal_samples
Danish VETII project	Pig	2016	PRJEB26961	SAMEA4688878	ERR2597364	gut metagenome	MG	Faecal_samples
Danish VETII project	Pig	2016	PRJEB26961	SAMEA4688879	ERR2597365	gut metagenome	MG	Faecal_samples
Danish VETII project	Pig	2016	PRJEB26961	SAMEA4688880	ERR2597366	gut metagenome	MG	Faecal_samples
Danish VETII project	Pig	2016	PRJEB26961	SAMEA4688881	ERR2597367	gut metagenome	MG	Faecal_samples
Danish VETII project	Pig	2016	PRJEB26961	SAMEA4688882	ERR2597368	gut metagenome	MG	Faecal_samples
Danish VETII project	Pig	2016	PRJEB26961	SAMEA4688883	ERR2597369	gut metagenome	MG	Faecal_samples
Danish VETII project	Pig	2016	PRJEB26961	SAMEA4688884	ERR2597370	gut metagenome	MG	Faecal_samples
Danish VETII project	Pig	2016	PRJEB26961	SAMEA4688885	ERR2597371	gut metagenome	MG	Faecal_samples
Danish VETII project	Pig	2016	PRJEB26961	SAMEA4688886	ERR2597372	gut metagenome	MG	Faecal_samples
Danish VETII project	Pig	2016	PRJEB26961	SAMEA4688887	ERR2597373	gut metagenome	MG	Faecal_samples
Danish VETII project	Pig	2016	PRJEB26961	SAMEA4688888	ERR2597374	gut metagenome	MG	Faecal_samples
Danish VETII project	Pig	2016	PRJEB26961	SAMEA4688889	ERR2597375	gut metagenome	MG	Faecal_samples
Danish VETII project	Pig	2016	PRJEB26961	SAMEA4688890	ERR2597376	gut metagenome	MG	Faecal_samples

Danish VETII project	Pig	2016	PRJEB26961	SAMEA4688891	ERR2597377	gut metagenome	MG	Faecal_samples
Danish VETII project	Pig	2016	PRJEB26961	SAMEA4688892	ERR2597378	gut metagenome	MG	Faecal_samples
Danish VETII project	Pig	2016	PRJEB26961	SAMEA4688893	ERR2597379	gut metagenome	MG	Faecal_samples
Danish VETII project	Pig	2016	PRJEB26961	SAMEA4688894	ERR2597380	gut metagenome	MG	Faecal_samples
Danish VETII project	Pig	2016	PRJEB26961	SAMEA4688895	ERR2597381	gut metagenome	MG	Faecal_samples
Danish VETII project	Pig	2016	PRJEB26961	SAMEA4688896	ERR2597382	gut metagenome	MG	Faecal_samples
Danish VETII project	Pig	2016	PRJEB26961	SAMEA4688897	ERR2597383	gut metagenome	MG	Faecal_samples
Danish VETII project	Pig	2016	PRJEB26961	SAMEA4688898	ERR2597384	gut metagenome	MG	Faecal_samples
Danish VETII project	Pig	2016	PRJEB26961	SAMEA4688899	ERR2597385	gut metagenome	MG	Faecal_samples
Danish VETII project	Pig	2016	PRJEB26961	SAMEA4688900	ERR2597386	gut metagenome	MG	Faecal_samples
Danish VETII project	Pig	2016	PRJEB26961	SAMEA4688901	ERR2597387	gut metagenome	MG	Faecal_samples
Danish VETII project	Pig	2016	PRJEB26961	SAMEA4688902	ERR2597388	gut metagenome	MG	Faecal_samples
Danish VETII project	Pig	2016	PRJEB26961	SAMEA4688903	ERR2597389	gut metagenome	MG	Faecal_samples
Danish VETII project	Pig	2016	PRJEB26961	SAMEA4688904	ERR2597390	gut metagenome	MG	Faecal_samples
Danish VETII project	Pig	2016	PRJEB26961	SAMEA4688905	ERR2597391	gut metagenome	MG	Faecal_samples
Danish VETII project	Pig	2016	PRJEB26961	SAMEA4688906	ERR2597392	gut metagenome	MG	Faecal_samples
Danish VETII project	Pig	2016	PRJEB26961	SAMEA4688907	ERR2597393	gut metagenome	MG	Faecal_samples
Danish VETII project	Pig	2016	PRJEB26961	SAMEA4688908	ERR2597394	gut metagenome	MG	Faecal_samples
Danish VETII project	Pig	2016	PRJEB26961	SAMEA4688909	ERR2597395	gut metagenome	MG	Faecal_samples
Danish VETII project	Pig	2016	PRJEB26961	SAMEA4688910	ERR2597396	gut metagenome	MG	Faecal_samples
Danish VETII project	Pig	2016	PRJEB26961	SAMEA4688911	ERR2597397	gut metagenome	MG	Faecal_samples
Danish VETII project	Pig	2016	PRJEB26961	SAMEA4688912	ERR2597398	gut metagenome	MG	Faecal_samples
Danish VETII project	Pig	2016	PRJEB26961	SAMEA4688913	ERR2597399	gut metagenome	MG	Faecal_samples
Danish VETII project	Pig	2016	PRJEB26961	SAMEA4688914	ERR2597400	gut metagenome	MG	Faecal_samples
Danish VETII project	Pig	2016	PRJEB26961	SAMEA4688915	ERR2597401	gut metagenome	MG	Faecal_samples
Danish VETII project	Pig	2016	PRJEB26961	SAMEA4688916	ERR2597402	gut metagenome	MG	Faecal_samples
Danish VETII project	Pig	2016	PRJEB26961	SAMEA4688917	ERR2597403	gut metagenome	MG	Faecal_samples
Danish VETII project	Pig	2016	PRJEB26961	SAMEA4688918	ERR2597404	gut metagenome	MG	Faecal_samples
Danish VETII project	Pig	2016	PRJEB26961	SAMEA4688919	ERR2597405	gut metagenome	MG	Faecal_samples
Danish VETII project	Pig	2016	PRJEB26961	SAMEA4688920	ERR2597406	gut metagenome	MG	Faecal_samples
Danish VETII project	Pig	2016	PRJEB26961	SAMEA4688921	ERR2597407	gut metagenome	MG	Faecal_samples
Danish VETII project	Pig	2016	PRJEB26961	SAMEA4688922	ERR2597408	gut metagenome	MG	Faecal_samples
Danish VETII project	Pig	2016	PRJEB26961	SAMEA4688923	ERR2597409	gut metagenome	MG	Faecal_samples
Danish VETII project	Pig	2016	PRJEB26961	SAMEA4688924	ERR2597410	gut metagenome	MG	Faecal_samples

Danish VETII project	Pig	2015	PRJEB26961	SAMEA4688925	ERR2597411	gut metagenome	MG	Faecal_samples
Danish VETII project	Pig	2016	PRJEB26961	SAMEA4688926	ERR2597412	gut metagenome	MG	Faecal_samples
Danish VETII project	Pig	2015	PRJEB26961	SAMEA4688927	ERR2597413	gut metagenome	MG	Faecal_samples
Danish VETII project	Pig	2015	PRJEB26961	SAMEA4688928	ERR2597414	gut metagenome	MG	Faecal_samples
Danish VETII project	Pig	2015	PRJEB26961	SAMEA4688929	ERR2597415	gut metagenome	MG	Faecal_samples
Danish VETII project	Pig	2015	PRJEB26961	SAMEA4688930	ERR2597416	gut metagenome	MG	Faecal_samples
Danish VETII project	Pig	2015	PRJEB26961	SAMEA4688932	ERR2597418	gut metagenome	MG	Faecal_samples
Danish VETII project	Pig	2016	PRJEB26961	SAMEA4688933	ERR2597419	gut metagenome	MG	Faecal_samples
Danish VETII project	Pig	2016	PRJEB26961	SAMEA4688934	ERR2597420	gut metagenome	MG	Faecal_samples
Danish VETII project	Pig	2016	PRJEB26961	SAMEA4688935	ERR2597421	gut metagenome	MG	Faecal_samples
Danish VETII project	Pig	2016	PRJEB26961	SAMEA4688936	ERR2597422	gut metagenome	MG	Faecal_samples
Danish VETII project	Pig	2016	PRJEB26961	SAMEA4688937	ERR2597423	gut metagenome	MG	Faecal_samples
Danish VETII project	Pig	2016	PRJEB26961	SAMEA4688938	ERR2597424	gut metagenome	MG	Faecal_samples
Danish VETII project	Pig	2016	PRJEB26961	SAMEA4688939	ERR2597425	gut metagenome	MG	Faecal_samples
Danish VETII project	Pig	2016	PRJEB26961	SAMEA4688940	ERR2597426	gut metagenome	MG	Faecal_samples
Danish VETII project	Pig	2016	PRJEB26961	SAMEA4688941	ERR2597427	gut metagenome	MG	Faecal_samples
Danish VETII project	Pig	2016	PRJEB26961	SAMEA4688942	ERR2597428	gut metagenome	MG	Faecal_samples
Danish VETII project	Pig	2016	PRJEB26961	SAMEA4688943	ERR2597429	gut metagenome	MG	Faecal_samples
Danish VETII project	Pig	2016	PRJEB26961	SAMEA4688944	ERR2597430	gut metagenome	MG	Faecal_samples
Danish VETII project	Pig	2016	PRJEB26961	SAMEA4688945	ERR2597431	gut metagenome	MG	Faecal_samples
Danish VETII project	Pig	2016	PRJEB26961	SAMEA4688946	ERR2597432	gut metagenome	MG	Faecal_samples
Danish VETII project	Pig	2016	PRJEB26961	SAMEA4688947	ERR2597433	gut metagenome	MG	Faecal_samples
Danish VETII project	Pig	2016	PRJEB26961	SAMEA4688948	ERR2597434	gut metagenome	MG	Faecal_samples
Danish VETII project	Pig	2016	PRJEB26961	SAMEA4688949	ERR2597435	gut metagenome	MG	Faecal_samples
Danish VETII project	Pig	2016	PRJEB26961	SAMEA4688950	ERR2597436	gut metagenome	MG	Faecal_samples
Danish VETII project	Pig	2016	PRJEB26961	SAMEA4688952	ERR2597438	gut metagenome	MG	Faecal_samples
Danish VETII project	Pig	2016	PRJEB26961	SAMEA4688954	ERR2597440	gut metagenome	MG	Faecal_samples
Danish VETII project	Pig	2016	PRJEB26961	SAMEA4688955	ERR2597441	gut metagenome	MG	Faecal_samples
Danish VETII project	Pig	2016	PRJEB26961	SAMEA4688956	ERR2597442	gut metagenome	MG	Faecal_samples
Danish VETII project	Pig	2016	PRJEB26961	SAMEA4688957	ERR2597443	gut metagenome	MG	Faecal_samples
Danish VETII project	Pig	2016	PRJEB26961	SAMEA4688958	ERR2597444	gut metagenome	MG	Faecal_samples
Danish VETII project	Pig	2016	PRJEB26961	SAMEA4688959	ERR2597445	gut metagenome	MG	Faecal_samples
Danish VETII project	Pig	2016	PRJEB26961	SAMEA4688960	ERR2597446	gut metagenome	MG	Faecal_samples
Danish VETII project	Pig	2016	PRJEB26961	SAMEA4688961	ERR2597447	gut metagenome	MG	Faecal_samples

Danish VETII project	Pig	2016	PRJEB26961	SAMEA4688962	ERR2597448	gut metagenome	MG	Faecal_samples
Danish VETII project	Pig	2016	PRJEB26961	SAMEA4688963	ERR2597449	gut metagenome	MG	Faecal_samples
Danish VETII project	Pig	2016	PRJEB26961	SAMEA4688964	ERR2597450	gut metagenome	MG	Faecal_samples
Danish VETII project	Pig	2016	PRJEB26961	SAMEA4688965	ERR2597451	gut metagenome	MG	Faecal_samples
Danish VETII project	Pig	2016	PRJEB26961	SAMEA4688966	ERR2597452	gut metagenome	MG	Faecal_samples
Danish VETII project	Pig	2016	PRJEB26961	SAMEA4688967	ERR2597453	gut metagenome	MG	Faecal_samples
Danish VETII project	Pig	2016	PRJEB26961	SAMEA4688968	ERR2597454	gut metagenome	MG	Faecal_samples
Danish VETII project	Pig	2016	PRJEB26961	SAMEA4688969	ERR2597455	gut metagenome	MG	Faecal_samples
Danish VETII project	Pig	2016	PRJEB26961	SAMEA4688970	ERR2597456	gut metagenome	MG	Faecal_samples
Danish VETII project	Pig	2016	PRJEB26961	SAMEA4688971	ERR2597457	gut metagenome	MG	Faecal_samples
Danish VETII project	Pig	2016	PRJEB26961	SAMEA4688972	ERR2597458	gut metagenome	MG	Faecal_samples
Danish VETII project	Pig	2016	PRJEB26961	SAMEA4688973	ERR2597459	gut metagenome	MG	Faecal_samples
Danish VETII project	Pig	2016	PRJEB26961	SAMEA4688974	ERR2597460	gut metagenome	MG	Faecal_samples
Danish VETII project	Pig	2016	PRJEB26961	SAMEA4688975	ERR2597461	gut metagenome	MG	Faecal_samples
Danish VETII project	Pig	2016	PRJEB26961	SAMEA4688976	ERR2597462	gut metagenome	MG	Faecal_samples
Danish VETII project	Pig	2016	PRJEB26961	SAMEA4688977	ERR2597463	gut metagenome	MG	Faecal_samples
Danish VETII project	Pig	2016	PRJEB26961	SAMEA4688978	ERR2597464	gut metagenome	MG	Faecal_samples
Danish VETII project	Pig	2016	PRJEB26961	SAMEA4688979	ERR2597465	gut metagenome	MG	Faecal_samples
Danish VETII project	Pig	2016	PRJEB26961	SAMEA4688980	ERR2597466	gut metagenome	MG	Faecal_samples
Danish VETII project	Pig	2016	PRJEB26961	SAMEA4688981	ERR2597467	gut metagenome	MG	Faecal_samples
Danish VETII project	Pig	2016	PRJEB26961	SAMEA4688982	ERR2597468	gut metagenome	MG	Faecal_samples
Danish VETII project	Pig	2016	PRJEB26961	SAMEA4688983	ERR2597469	gut metagenome	MG	Faecal_samples
Danish VETII project	Pig	2016	PRJEB26961	SAMEA4688984	ERR2597470	gut metagenome	MG	Faecal_samples
Danish VETII project	Pig	2016	PRJEB26961	SAMEA4688985	ERR2597471	gut metagenome	MG	Faecal_samples
Danish VETII project	Pig	2016	PRJEB26961	SAMEA4688986	ERR2597472	gut metagenome	MG	Faecal_samples
Danish VETII project	Pig	2016	PRJEB26961	SAMEA4688987	ERR2597473	gut metagenome	MG	Faecal_samples
Danish VETII project	Pig	2016	PRJEB26961	SAMEA4688988	ERR2597474	gut metagenome	MG	Faecal_samples
Danish VETII project	Pig	2016	PRJEB26961	SAMEA4688989	ERR2597475	gut metagenome	MG	Faecal_samples
Danish VETII project	Pig	2016	PRJEB26961	SAMEA4688990	ERR2597476	gut metagenome	MG	Faecal_samples
Danish VETII project	Pig	2015	PRJEB26961	SAMEA4688991	ERR2597477	gut metagenome	MG	Faecal_samples
Danish VETII project	Pig	2015	PRJEB26961	SAMEA4688992	ERR2597478	gut metagenome	MG	Faecal_samples
Danish VETII project	Pig	2015	PRJEB26961	SAMEA4688994	ERR2597480	gut metagenome	MG	Faecal_samples
Danish VETII project	Pig	2015	PRJEB26961	SAMEA4688995	ERR2597481	gut metagenome	MG	Faecal_samples
Danish VETII project	Pig	2015	PRJEB26961	SAMEA4688998	ERR2597484	gut metagenome	MG	Faecal_samples

Danish VETII project	Pig	2015	PRJEB26961	SAMEA4688999	ERR2597485	gut metagenome	MG	Faecal_samples
Danish VETII project	Pig	2015	PRJEB26961	SAMEA4689000	ERR2597486	gut metagenome	MG	Faecal_samples
Danish VETII project	Pig	2015	PRJEB26961	SAMEA4689001	ERR2597487	gut metagenome	MG	Faecal_samples
Danish VETII project	Pig	2015	PRJEB26961	SAMEA4689002	ERR2597488	gut metagenome	MG	Faecal_samples
Danish VETII project	Pig	2015	PRJEB26961	SAMEA4689004	ERR2597490	gut metagenome	MG	Faecal_samples
Danish VETII project	Pig	2015	PRJEB26961	SAMEA4689005	ERR2597491	gut metagenome	MG	Faecal_samples
Danish VETII project	Pig	2015	PRJEB26961	SAMEA4689006	ERR2597492	gut metagenome	MG	Faecal_samples
Danish VETII project	Pig	2015	PRJEB26961	SAMEA4689007	ERR2597493	gut metagenome	MG	Faecal_samples
Danish VETII project	Pig	2015	PRJEB26961	SAMEA4689009	ERR2597495	gut metagenome	MG	Faecal_samples
Danish VETII project	Pig	2015	PRJEB26961	SAMEA4689010	ERR2597496	gut metagenome	MG	Faecal_samples
Danish VETII project	Pig	2015	PRJEB26961	SAMEA4689011	ERR2597497	gut metagenome	MG	Faecal_samples
Danish VETII project	Pig	2015	PRJEB26961	SAMEA4689012	ERR2597498	gut metagenome	MG	Faecal_samples
Danish VETII project	Pig	2015	PRJEB26961	SAMEA4689013	ERR2597499	gut metagenome	MG	Faecal_samples
Danish VETII project	Pig	2015	PRJEB26961	SAMEA4689014	ERR2597500	gut metagenome	MG	Faecal_samples
Danish VETII project	Pig	2015	PRJEB26961	SAMEA4689015	ERR2597501	gut metagenome	MG	Faecal_samples
Danish VETII project	Pig	2015	PRJEB26961	SAMEA4689016	ERR2597502	gut metagenome	MG	Faecal_samples
Danish VETII project	Pig	2015	PRJEB26961	SAMEA4689017	ERR2597503	gut metagenome	MG	Faecal_samples
Danish VETII project	Pig	2015	PRJEB26961	SAMEA4689018	ERR2597504	gut metagenome	MG	Faecal_samples
Danish VETII project	Pig	2015	PRJEB26961	SAMEA4689019	ERR2597505	gut metagenome	MG	Faecal_samples
Danish VETII project	Pig	2015	PRJEB26961	SAMEA4689020	ERR2597506	gut metagenome	MG	Faecal_samples
Danish VETII project	Pig	2015	PRJEB26961	SAMEA4689021	ERR2597507	gut metagenome	MG	Faecal_samples
Danish VETII project	Pig	2015	PRJEB26961	SAMEA4689022	ERR2597508	gut metagenome	MG	Faecal_samples
Danish VETII project	Pig	2015	PRJEB26961	SAMEA4689023	ERR2597509	gut metagenome	MG	Faecal_samples
Danish VETII project	Pig	2015	PRJEB26961	SAMEA4689024	ERR2597510	gut metagenome	MG	Faecal_samples
Danish VETII project	Pig	2015	PRJEB26961	SAMEA4689025	ERR2597511	gut metagenome	MG	Faecal_samples
Danish VETII project	Pig	2015	PRJEB26961	SAMEA4689026	ERR2597512	gut metagenome	MG	Faecal_samples
Danish VETII project	Pig	2015	PRJEB26961	SAMEA4689027	ERR2597513	gut metagenome	MG	Faecal_samples
Danish VETII project	Pig	2015	PRJEB26961	SAMEA4689028	ERR2597514	gut metagenome	MG	Faecal_samples
Danish VETII project	Pig	2015	PRJEB26961	SAMEA4689029	ERR2597515	gut metagenome	MG	Faecal_samples
Danish VETII project	Pig	2015	PRJEB26961	SAMEA4689030	ERR2597516	gut metagenome	MG	Faecal_samples
Danish VETII project	Pig	2015	PRJEB26961	SAMEA4689031	ERR2597517	gut metagenome	MG	Faecal_samples
Danish VETII project	Pig	2015	PRJEB26961	SAMEA4689032	ERR2597518	gut metagenome	MG	Faecal_samples
Danish VETII project	Pig	2015	PRJEB26961	SAMEA4689033	ERR2597519	gut metagenome	MG	Faecal_samples
Danish VETII project	Pig	2015	PRJEB26961	SAMEA4689034	ERR2597520	gut metagenome	MG	Faecal_samples

Danish VETII project	Pig	2015	PRJEB26961	SAMEA4689035	ERR2597521	gut metagenome	MG	Faecal_samples
Danish VETII project	Pig	2015	PRJEB26961	SAMEA4689036	ERR2597522	gut metagenome	MG	Faecal_samples
Danish VETII project	Pig	2015	PRJEB26961	SAMEA4689038	ERR2597524	gut metagenome	MG	Faecal_samples
Danish VETII project	Pig	2015	PRJEB26961	SAMEA4689039	ERR2597525	gut metagenome	MG	Faecal_samples
Danish VETII project	Pig	2015	PRJEB26961	SAMEA4689040	ERR2597526	gut metagenome	MG	Faecal_samples
Danish VETII project	Pig	2015	PRJEB26961	SAMEA4689041	ERR2597527	gut metagenome	MG	Faecal_samples
Danish VETII project	Pig	2015	PRJEB26961	SAMEA4689042	ERR2597528	gut metagenome	MG	Faecal_samples
Danish VETII project	Pig	2015	PRJEB26961	SAMEA4689043	ERR2597529	gut metagenome	MG	Faecal_samples
Danish VETII project	Pig	2015	PRJEB26961	SAMEA4689044	ERR2597530	gut metagenome	MG	Faecal_samples
Danish VETII project	Pig	2015	PRJEB26961	SAMEA4689045	ERR2597531	gut metagenome	MG	Faecal_samples
Danish VETII project	Pig	2015	PRJEB26961	SAMEA4689047	ERR2597533	gut metagenome	MG	Faecal_samples
Danish VETII project	Pig	2015	PRJEB26961	SAMEA4689048	ERR2597534	gut metagenome	MG	Faecal_samples
Danish VETII project	Pig	2015	PRJEB26961	SAMEA4689049	ERR2597535	gut metagenome	MG	Faecal_samples
Danish VETII project	Pig	2015	PRJEB26961	SAMEA4689050	ERR2597536	gut metagenome	MG	Faecal_samples
Danish VETII project	Pig	2015	PRJEB26961	SAMEA4689051	ERR2597537	gut metagenome	MG	Faecal_samples
Danish VETII project	Pig	2015	PRJEB26961	SAMEA4689052	ERR2597538	gut metagenome	MG	Faecal_samples
Danish VETII project	Pig	2015	PRJEB26961	SAMEA4689053	ERR2597539	gut metagenome	MG	Faecal_samples
Danish VETII project	Pig	2015	PRJEB26961	SAMEA4689054	ERR2597540	gut metagenome	MG	Faecal_samples
Danish VETII project	Pig	2015	PRJEB26961	SAMEA4689055	ERR2597541	gut metagenome	MG	Faecal_samples
Danish VETII project	Pig	2015	PRJEB26961	SAMEA4689056	ERR2597542	gut metagenome	MG	Faecal_samples
